# Word Frequency and Predictability Dissociate in Naturalistic Reading

**DOI:** 10.1162/opmi_a_00119

**Published:** 2024-03-05

**Authors:** Cory Shain

**Affiliations:** Department of Brain & Cognitive Sciences and McGovern Institute for Brain Research, Massachusetts Institute of Technology, Cambridge, MA, USA

**Keywords:** psycholinguistics, reading, surprisal, lexical retrieval, computational modeling, prediction

## Abstract

Many studies of human language processing have shown that readers slow down at less frequent or less predictable words, but there is debate about whether frequency and predictability effects reflect separable cognitive phenomena: are cognitive operations that retrieve words from the mental lexicon based on sensory cues distinct from those that predict upcoming words based on context? Previous evidence for a frequency-predictability dissociation is mostly based on small samples (both for estimating predictability and frequency and for testing their effects on human behavior), artificial materials (e.g., isolated constructed sentences), and implausible modeling assumptions (discrete-time dynamics, linearity, additivity, constant variance, and invariance over time), which raises the question: do frequency and predictability dissociate in ordinary language comprehension, such as story reading? This study leverages recent progress in open data and computational modeling to address this question at scale. A large collection of naturalistic reading data (six datasets, >2.2 M datapoints) is analyzed using nonlinear continuous-time regression, and frequency and predictability are estimated using statistical language models trained on more data than is currently typical in psycholinguistics. Despite the use of naturalistic data, strong predictability estimates, and flexible regression models, results converge with earlier experimental studies in supporting dissociable and additive frequency and predictability effects.

## INTRODUCTION

Consider the words *beer* and *cauldron* in the following sentences:

- She purchased a beer/**cauldron**.
- She stirred the boiling **beer**/cauldron.

Intuitively, the boldfaced word in each example seems more difficult to process, for reasons that likely have to do with the statistical properties of language: some words are used more frequently than others, and some words are more predictable in a specific context than others. In a less constraining context like *She purchased a*, the less frequent word *cauldron* incurs a processing cost relative to the more frequent word *beer*. However, a more constraining context like *She stirred the boiling* can reverse this pattern, since *cauldron* becomes more predictable than *beer*. These impressions have been borne out empirically by many studies that showed longer reading times for words that are less frequent (Goodkind & Bicknell, 2021; Juhasz & Rayner, 2006; Just & Carpenter, 1980; Miellet et al., 2007; Rayner, 1977; Rayner & Duffy, 1986; Rayner & Raney, 1996, Schilling et al., 1998; White et al., 2018) or less predictable (Ehrlich & Rayner, 1981; Kretzschmar et al., 2015; Miellet et al., 2007; Staub, 2011; Wilcox et al., 2020, 2023; Shain et al., in press; Zola, 1984).

While these effects may seem unsurprising, they are bound up in fundamental questions about the real-time mental processes by which we infer meaning from language. One *procedural* view of language comprehension focuses on putatively distinct cognitive operations. The goal of theorizing in this view is to understand the moment-by-moment coordination of these operations in assembling a representation of meaning: words must be recognized from visual or auditory input, retrieved from long-term memory, and integrated into a partial representation of sentence structure (Coltheart et al., 2001; Engbert et al., 2002; Gibson, 2000; Harm & Seidenberg, 2004; Just & Carpenter, 1980; Lewis & Vasishth, 2005; Morrison, 1984; Nilsson & Nivre, 2010; Reichle et al., 1998; Staub, 2015; Van Dyke & McElree, 2011). Another *inferential* view of language comprehension focuses on the problem of optimally allocating probability among possible message-level meanings given limited evidence (i.e., incomplete utterances; Bicknell & Levy, 2012; Futrell et al., 2020; Gibson et al., 2013; Hahn et al., 2022; Hale, 2001; Levy, 2008, see also Legge et al., 1997; Norris, 2006 for related perspectives on word recognition). The goal of theorizing in this view is to understand the structure and parameterization of the mental probability model that licenses these inferences.

These two views prioritize different aspects of the comprehension problem (respectively, how representations are computed vs. which representations to compute) and are thus in many ways incommensurable and possibly complementary: any implemented comprehension system must both compute meaning representations (the focus of the procedural view) and contend with uncertainty (the focus of the inferential view). A major goal of cognitive psychology is in fact to figure out how both these objectives are achieved. However, the two views tend to differ in substance with respect to their commitments about *processing difficulty*; in particular, whether processing difficulty is thought to be driven primarily by operations that recognize words and integrate them into sentence representations in working memory (procedural view) or primarily by the informativity of a word in context, irrespective of how that information is represented in working memory (inferential view). This difference can lead to divergent interpretations of the aforementioned relationship between word frequency and predictability.

Under a procedural view, frequency effects are generally thought to arise from differential encoding strength of words in the mental lexicon: more frequently encountered words will have stronger memory representations (often conceived as higher baseline activation or lower activation thresholds) and thus be easier to retrieve based on sensory (e.g., visual word form) cues (Coltheart et al., 2001; Engbert et al., 2002; Just & Carpenter, 1980; Morrison, 1984; Nilsson & Nivre, 2010; Reichle et al., 1998). This construal of frequency effects as reflecting memory retrieval (as opposed to e.g., lower-level perceptual processes like visual word recognition) is motivated by their relatively long timecourse: frequency effects have been shown to extend into “late” eye movement measures like go-past duration (e.g., Slattery et al., 2007, this study) and “spill over” into the processing of subsequent words (e.g., Rayner & Duffy, 1986, this study), suggesting that word frequency continues to influence processing even when the word is no longer in the fovea. Such delayed effects seem unlikely to be driven primarily by visual word recognition, although visual processing may of course contribute to them at early stages. Prediction is thought to serve a distinct role in processing by preactivating words based on context, thereby facilitating their integration into the meaning representation (Brothers & Kuperberg, 2021; Ehrlich & Rayner, 1981; Staub, 2015). Although some procedural models implement predictability effects as a multiplier on frequency-based retrieval processes and thus predict a frequency-predictability interaction (e.g., Reichle et al., 1998), little empirical evidence of such an interaction has emerged (see below). For these and other reasons, recent statements of the procedural view tend to construe frequency and predictability effects as reflecting independent types or stages of processing (e.g., Staub, 2015).

Under an inferential view, the core determinant of processing difficulty is how much the probability distribution over possible interpretations must change in response to observing a word (Hale, 2001; Levy, 2008). Using information theory, this change can be quantified as *surprisal*, the negative log probability of a word in context (Levy, 2008). On this view, frequency effects are merely a species of predictability effect that arises when context is absent or unconstraining, causing the processor to fall back to the prior probability of the word (a function of lexical frequency; Bicknell & Levy, 2012; Levy, 2008; Norris, 2006). Returning to the example above, because *cauldron* is less frequent than *beer*, it is also less predictable in an unconstraining context like *She purchased a*. Thus, the inferential view construes predictability as the root cause of apparent frequency effects, and frequency effects should not emerge when predictability is taken into account.

The frequency-related and predictability-related components of these two views can treated separately. For example, an inferential view of predictability effects (whereby processing cost is driven by the size of the update to the interpretation distribution) is logically compatible with a procedural view of frequency effects (whereby processing cost is additionally driven by the difficulty of lexical retrieval). Indeed, because considerable prior evidence favors an inferential (Bayesian) rather than a procedural (preactivation-based) interpretation of predictability effects in language comprehension (e.g., Shain et al., in press; Smith & Levy, 2013; Szewczyk & Federmeier, 2022; Wilcox et al., 2020, cf. Brothers & Kuperberg, 2021) an inferential view of predictability effects is assumed in the present study. As a result, unless otherwise noted, the terms *procedural* and *inferential* are used throughout this work to refer specifically to the implications of these views for frequency effects, setting aside their implications for predictability effects, which have been studied elsewhere (see Discussion for elaboration on this point). Thus, the key question at issue in this study is which view (procedural or inferential) better predicts the relationship between frequency and predictability specifically: are frequency and predictability effects dissociable (as predicted by the procedural view), or does predictability explain frequency effects away (as predicted by the inferential view)? This question has been addressed in three ways.

The first line of research uses a factorial frequency-predictability manipulation in which sentence frames that provide neutral (LoPred) or constraining (HiPred) cues are crossed with target words from low (LoFreq) and high (HiFreq) frequency bins (Altarriba et al., 1996; Ashby et al., 2005; Bélanger & Rayner, 2013; Gollan et al., 2011; Hand et al., 2010; Kretzschmar et al., 2015; Lavigne et al., 2000; Miellet et al., 2007; Rayner et al., 2001, 2004, see Staub, 2015, for review). In this design, both views predict an increase in reading speed from the LoPred-LoFreq condition to the HiPred-LoFreq condition due to the increase in predictability. Perhaps surprisingly, both views also predict an increase in reading speed from the LoPred-LoFreq condition to the LoPred-HiFreq condition. The procedural view predicts this because frequency is thought to be a prediction-independent driver of processing difficulty. But the inferential view also predicts this because the LoPred condition does not in fact match items on predictability: high-frequency words are also more predictable in neutral contexts, such that the difference from LoPred-LoFreq to LoPred-HiFreq is really a matter of predictability (rather than frequency). The relative difficulty of matching LoPred vs. HiPred items on predictability in the inferential view (vs. the procedural view) falls out from differences in the assumed role played by prediction (facilitation vs. cost), and thus in the assumed relationship between predictability and processing difficulty (linear vs. logarithmic; Smith & Levy, 2013; Shain et al., in press, see Discussion). Nonetheless, the two views differ regarding the HiPred-HiFreq condition. Unlike the procedural view, the inferential view predicts no difference in reading time between the HiPred-HiFreq and HiPred-LoFreq conditions, since, in both cases, context has rendered the word highly predictable, regardless of its base frequency. Thus, in this design, the inferential view predicts a frequency-predictability interaction that attenuates the frequency effect for highly predictable words, whereas the procedural view predicts additive effects with no interaction (Kretzschmar et al., 2015; Staub, 2015). Thus far, most such studies find no significant interaction, which has been taken to support the procedural view (Staub, 2015), although some significant frequency-predictability interactions have been reported in eye movements (Hand et al., 2010) and event-related potentials (ERPs; Dambacher et al., 2006; Sereno et al., 2020). See Discussion for elaboration on this point.

However, there are reasons to treat this evidence with caution. First, it is based on null findings (non-significance of interactions) in studies with generally small samples (typically, 20–40 participants with 8–10 items per condition). Second, it derives from isolated sentence reading in which the rich contexts and communicative goals that characterize ordinary language use (and may aid prediction) are absent (Hamilton & Huth, 2020; Hasson et al., 2018; Hasson & Honey, 2012; Jain et al., 2023). Third, by binning on predictability, it collapses continuous differences in predictability that can be large on the logarithmic surprisal scale which is predicted by the inferential view and supported by empirical studies (Hoover et al., 2023; Shain et al., in press; Smith & Levy, 2013; Wilcox et al., 2020, 2023). This mismatch could be detrimental to effects (including interactions) estimated by linear models. Fourth, statistical inferences are generally based on analyses of variance (ANOVAs), which cannot simultaneously account for random variation due to the particular sample of participants and items in a study (Barr et al., 2013; Clark, 1973).

The second line of research uses the same basic design as above but looks for differences in how frequency and predictability affect the parameters of the exGaussian distribution (Staub, 2011; Staub et al., 2010). The exGaussian is the convolution of a normal distribution (with location *μ* and dispersion *σ* parameters) with an exponential distribution, thereby introducing a rightward skewness parameter (*τ*). This property makes the exGaussian a popular choice for modeling reaction times, which often have heavy right tails (Balota & Yap, 2011; Heathcote et al., 1991; Hohle, 1965; Ratcliff, 1979). Staub et al. (2010) found that frequency generally modulates both location and skewness, whereas Staub (2011) found predictability to only modulate location (see also Sheridan & Reingold, 2012). Staub (2011) reasoned that frequency and predictability effects derive from distinct mechanisms, since they have distinct distributional effects.

The concerns about this second line of evidence overlap substantially with the concerns about the first (small sample sizes, artificial tasks, and analyses that do not jointly account for participant and item effects). In addition, a difference in significance does not entail a significant difference (Nieuwenhuis et al., 2011). Since the effects of predictability and frequency on skewness have not been directly compared within a single study, it may be premature to conclude a difference.

The third line of research attempts to address some of these concerns by applying more expressive mixed-effects models to word-by-word naturalistic reading (e.g., of stories or newspaper articles) using statistically derived frequency and predictability estimates (Goodkind & Bicknell, 2021; Shain, 2019). This approach improves ecological validity as well as the resolution of the predictability estimate (since controlled studies tend to use low-precision cloze predictability estimates), at the expense of experimental control. Under this design, the empirical predictions of the two views at issue here are straightforward: the procedural view predicts separable frequency and predictability effects, whereas the inferential view predicts that predictability (represented as surprisal) will explain away any frequency effects. Results have been mixed. Goodkind and Bicknell (2021) analyzed the Dundee eye-tracking dataset (Kennedy & Pynte, 2005) using linear mixed-effects models (LMEMs; Bates et al., 2015) and found evidence from likelihood ratio tests (LRTs; Wilks, 1938) of unigram (log-frequency) effects that were not explained by *n*-gram models of word predictability. However, Shain (2019) analyzed the Dundee, Natural Stories (Futrell et al., 2021), and UCL (Frank et al., 2013) datasets using continuous-time deconvolutional regression (CDR) models (Shain & Schuler, 2021) and found that frequency effects did not improve models’ generalization to unseen data, over and above predictability. There is thus an apparent discrepancy between the results of the controlled studies outlined above and at least one replication attempt (Shain, 2019) in a more naturalistic design, which raises concerns about potential task or modeling artifacts.

However, because naturalistic studies rely on large, complex observational datasets, results are highly sensitive to statistical design. LMEMs make a number of simplifying assumptions, including discrete-time dynamics, linear and additive effects, homoscedasticity (constant error), and stationarity (time-invariance). These assumptions are cognitively implausible and can critically affect the outcome of statistical tests (Shain & Schuler, in press). The CDR models of Shain (2019) relax the assumption of discrete-time dynamics but leave the remaining assumptions intact. To the extent that these assumptions are violated by the data-generating process, this may affect results in unpredictable ways (Shain & Schuler, in press). Moreover, both extant naturalistic studies used *n*-gram predictability models with highly constrained (1–4 word) contexts that likely under-represent the amount of contextual information available to humans.

In summary, despite a sizeable body of prior work, questions remain about the dissociability of frequency and predictability effects in reading (especially in naturalistic settings), and thus about their implications for the cognitive architecture of human language comprehension. The present study therefore leverages recent progress in computational modeling and open data to revisit this question in naturalistic reading at scale. The core methodological contributions are as follows.

First, unlike prior work, predictability is estimated using GPT-2, an incremental neural network language model (Radford et al., 2019) based on the transformer architecture (Vaswani et al., 2017). GPT-2 has been shown to strongly correlate with human measures of language processing, both in behavior (Hoover et al., 2023; Shain et al., in press; Wilcox et al., 2020) and the brain (Jain et al., 2023; Schrimpf et al., 2021; Shain et al., 2022; Tuckute et al., 2024). In fact, one recent study showed that GPT-2 surprisal significantly outperformed cloze surprisal in predicting eye movements in the Provo eye-tracking dataset (Shain et al., in press). This result converges with recent evidence (Hofmann et al., 2022; Michaelov et al., 2023) suggesting that large-scale statistical language models may be at or beyond parity with cloze (Taylor, 1953), the established gold-standard measure of human subjective predictability in psycholinguistics.

Second, analyses cover the largest collection of naturalistic reading data brought to bear on this question to-date, consisting of six datasets representing three reading modalities (eye-tracking during reading, self-paced reading, and the Maze task), with a combined total of over 2.2 million reading events. All statistical tests are based on the generalization performance of pre-trained statistical models to unseen data, thus grounding results directly in the generalizability of findings.

Third, analyses use the continuous-time deconvolutional regressive neural network (CDRNN), an interpretable approach to analyzing observational time series that generalizes continuous-time deconvolutional regression (Shain & Schuler, 2018, 2021) using artificial neural networks (Shain & Schuler, 2021, in press). These continuous-time deconvolutional models have been used in other recent studies of language processing effects in human reading behavior (Shain, 2019; Shain et al., in press) and brain activity (Shain et al., 2020, 2022). CDRNNs simultaneously relax all the problematic simplifying assumptions enumerated above (discrete-time dynamics, linearity, additivity, homoscedasticity, and stationarity) and dramatically improve fit to human reading data over standard approaches (Shain & Schuler, in press). In addition to reducing reliance on implausible modeling assumptions, CDRNNs offer an additional key advantage for this research question: they simultaneously estimate how variables influence all parameters of the predictive distribution (i.e., the location, dispersion, and skewness parameters of the exGaussian) in a fully mixed-effects design, and they can therefore be used to revisit prior claims of dissociable effects of frequency and predictability on the shape of the distribution over reading times.

To anticipate the main finding, CDRNN models show strong and dissociable effects of frequency and predictability across datasets, with little evidence of an interaction. However, results do not support previous claims of dissociable distributional effects. On the whole, both frequency and predictability primarily modulate the skewness parameter of the distribution over reading times, with weaker effects on the location and scale parameters. Thus, although frequency and predictability dissociate in their effects on overall reading times, they affect the distribution over reading times in qualitatively similar ways.

Together with prior work (especially Shain et al., in press), these results cross-cut the common dividing lines between the procedural and inferential communities in sentence processing research and thus encourage both theoretical positions to revisit core assumptions. The results of the present study indicate that frequency effects have a life of their own, even under rigorous control for predictability as estimated by transformer language models, the current state of the art for modeling predictability effects in human reading (Shain et al., in press). This study thus aligns with the procedural view, according to which prediction and retrieval are separable processes, and presents a major challenge for the inferential view, which, under standard assumptions, affords no explanation for prediction-independent frequency effects. But recent naturalistic work on a similar scale to the present study also provides strong evidence that small absolute differences in low predictability yield large differences in processing difficulty (Shain et al., in press, cf. Brothers & Kuperberg, 2021). This outcome is difficult to account for under the standard procedural assumption that prediction is primarily a facilitatory preacivation-based process: if a word is unpredictable, it should get little facilitation, regardless of whether it had probability 0.01 or 0.001. However, this outcome falls out naturally from the inferential view, which regards predictability as reflecting the (logarithmically-scaled) update to the processor state after observing a word. Thus, both views face challenges in reconciling key assumptions with attested empirical patterns.

On the inferential side, one relevant recent innovation is the development of *resource-rational* variants of the inferential view that also predict a frequency-predictability dissociation, but for different reasons (Futrell et al., 2020; Hahn et al., 2022). Resource-rational inferential models hypothesize that humans have imperfect or “lossy” access to the linguistic context due to both perceptual and memory limitations, with a probability of forgetting that increases roughly in proportion to the length of context. This view predicts that standard language models like GPT-2, which have veridical access to arbitrarily long contexts, over-estimate contextual influences on word prediction relative to humans. Since more recently encountered words are less likely to have been forgotten than less recently encountered words, humans are expected interpolate (or *smooth*) predictions based on longer contexts (about which they are less certain) with predictions based on shorter contexts (about which they are more certain). As a consequence, frequency should dissociate from predictability by indirectly capturing the effects of this forgetting-based smoothing over context representations. Thus, frequency effects that are not explained by predictability can still be construed as a species of predictability effect (rather than a reflex of lexical retrieval) as long as the predictability estimate has implausibly direct access to context. This view is nonetheless dissociable from the procedural view because it frames frequency and (GPT-2) predictability as points along a continuum of contextual forgetting, rather than as reflexes of distinct cognitive operations. Thus, the resource-rational view predicts additional contributions from other points along this continuum (e.g., bigram and trigram context models), whereas the procedural view does not. Results in the present study provide some support for this prediction, and thus favor a resource-rational interpretation of the frequency-predictability dissociation, in line with other recent evidence (Goodkind & Bicknell, 2021).

## METHODS

Dataset selection, preprocessing, and modeling closely follow Shain et al. (in press). Details are provided below for reference. Data used in these analyses are available for download at https://osf.io/8v5qb/, and code to reproduce the analyses is available at https://github.com/coryshain/cdr.

### Data

Analyses cover six publicly available naturalistic reading datasets: the Brown self-paced reading (SPR) dataset (Smith & Levy, 2013), the Dundee eye-tracking (ET) dataset (Kennedy & Pynte, 2005), the GECO self-paced reading dataset (English version; Cop et al., 2017), the Natural Stories self-paced reading dataset (Futrell et al., 2021), the Natural Stories Maze dataset (Boyce & Levy, 2023), and the Provo eye-tracking dataset (Luke & Christianson, 2018). Per Shain and Schuler (2021), to enable valid deconvolution, all data filtering and partitioning described below are applied only to the response vectors (the modeled reading times). The entire predictor matrix (sequence of word fixation features) is retained in all models.

In a self-paced reading task, participants are presented with texts in which words or characters are occluded until the participant reveals them one-by-one in left-to-right order by pressing a button. In a Maze task (Freedman & Forster, 1985), like in a self-paced reading task, participants press buttons to progress word-by-word through a text. However, at each word position in the text, participants are presented with a forced choice between the true next word and a distractor, and they are tasked with selecting the correct continuation. In an eye-tracking during reading task, texts are presented on a screen to participants while their eye movements are recorded, and their sequence of fixations to words in the text are computed.

The self-paced reading and Maze tasks yield a single word-by-word response variable: *reading time* (or *reaction time*, RT), that is, the time elapsed between stimulus presentation (a word in self-paced reading or a forced-choice decision in Maze) and pressing a button to indicate a decision (to reveal the next word in self-paced reading or to choose the continuation in Maze). Modeling eye movements during free reading is more challenging because the eyes do not progress linearly through the textual sequence of words. Studies of eye-tracking during reading have used a variety of measures derived from the reading record, each with a somewhat different cognitive interpretation (see e.g., Rayner, 1998, for review). This study considers three different measures of fixation duration:

- **Scan path duration**. Time elapsed between entering a word region (from either direction) and entering a different word region (in either direction).
- **First path duration**. Time elapsed between entering a word region from the left and entering a different word region (in either direction).
- **Go-past duration**. Time elapsed between entering a word region from the left and entering a word region to its right (including all intervening regressive fixations).

Scan path and first pass durations are both early measures, restricted to the fixation duration of a single word (Rayner, 1998). They differ only in whether regressive eye movements are included (scan path) or discarded (first pass). Go-past duration is a late measure designed to capture all processing (including regressive eye movements) involved in moving beyond the current “frontier” in progressing through the text.

In all eye tracking datasets except the GECO dataset (see below), a stimulus “event” is considered to be any fixation to a word region in the text. Thus, the full sequence of fixations before entering a target word region, regressive or non-regressive, is used to predict all three types of fixation duration at that region. Note that this differs from standard regression analyses of first-pass and go-past durations in eye-tracking data, which typically discard the full sequence of fixations and only consider the linear sequence of words. The ability to recruit the full scan path record to predict all response variables is an advantage of the deconvolutional regression approach described below.

In all datasets, following prior analyses of the Dundee and Natural Stories SPR datasets (Shain & Schuler, 2021), the data are partitioned into training, validation, and test splits (approximately 50%, 25%, and 25%, respectively) using modular arithmetic on a split variable *i*, defined as a function of participant index *p* and sentence index *s*:

$$i=\left(s+p\right)\;\mathrm{mod}\;4$$ (1)

where datapoints are cycled into training if *i* ∈ {0, 1}, validation if *i* = 2, and test if *i* = 3. Models are only fitted to data from the training set. Validation data is used for tuning and early stopping, following Shain and Schuler (in press). Test data is only used for statistical comparisons between fitted models.

#### Brown SPR.

The Brown SPR dataset (Smith & Levy, 2013) contains self-paced reading data from 35 participants reading short (292–902 word) passages from the Brown dataset of American English (Francis & Kucera, 1979). The data can be accessed online at https://github.com/wilcoxeg/neural-networks-read-times. Disclosure: these dataset descriptions are identical to those used in Shain et al. (in press, *PNAS*), which used the same data and preprocessing.

The dataset contains a total of 450 sentences, 7,188 words, and 136,907 responses. Following established protocol for Natural Stories SPR (another self-paced reading dataset, described below), responses are removed if they fall at sentence boundaries or if their RTs were less than 100 ms or greater than 3,000 ms.

#### Dundee ET.

The Dundee ET dataset (Kennedy & Pynte, 2005) contains eye-tracking data from 10 participants who read newspaper articles from *The Independent* on a computer monitor. The data can be accessed online at https://github.com/wilcoxeg/neural-networks-read-times.

The dataset contains a total of 2,388 sentences, 51,501 words, and 408,439 distinct fixations to word regions on the screen. The responses in the Dundee dataset are filtered to exclude fixations following large outlier saccades (>20 words in either direction), based on the assumption that such outliers reflect track loss or inattention, rather than language processing. Following prior work (e.g., Shain & Schuler, 2021), fixations to words adjacent to a screen, line, or sentence boundary are removed, as well as fixations interrupted by blinks.

#### GECO ET.

The GECO ET dataset (Cop et al., 2017) contains eye-tracking data from participants who read *The Mysterious Affair at Styles* by Agatha Christie on a computer monitor. The full dataset contains data from 19 Dutch-English bilinguals who read the first half of the novel in either Dutch or English and the second half in the other language, along with data from 14 English monolinguals who read the entire novel in English. Because the computational language models used in this study are English-specific, here we only used the data from the 14 monolingual English readers. Unlike the other ET datasets analyzed in this study, the GECO dataset does not provide the full scan path record, but only a distilled format that contains first pass and go-past times by word. Thus, in the case of GECO, scan path durations are not analyzed, and each fixated word in textual order is treated as a stimulus “event” (rather than individual fixations) for the purposes of deconvolution. The data can be accessed online at https://expsy.ugent.be/downloads/geco/.

The portion of the dataset analyzed here contains a total of 5,300 sentences, 56,440 words, and 374,179 events. Following the Dundee protocol (above), the responses in the GECO dataset are filtered to exclude fixations following large outlier saccades (>20 words in either direction) and fixations to sentence boundaries (screen and line boundaries were not annotated).

#### Natural Stories SPR.

The Natural Stories SPR dataset (Futrell et al., 2021) contains crowd-sourced self-paced reading responses from 178 participants to 10 naturally-occuring narrative or non-fiction pieces modified in order to over-represent rare words and syntactic constructions without compromising perceived naturalness. The stimuli are thus designed to reflect the typical conditions of story comprehension, while subtly taxing the language processing system. The data can be accessed online at https://github.com/languageMIT/naturalstories.

The dataset contains a total of 485 sentences, 10,256 words, and 1,013,377 responses. Following previous work (e.g., Shain & Schuler, 2021), RTs are removed if they are less than 100ms or greater than 3,000ms, if they are to words adjacent to a sentence boundary, if participants answered less than 5/8 comprehension questions correctly, or if, subject to the aforementioned constraints, participants have fewer than 100 RTs.

#### Natural Stories Maze.

The Natural Stories Maze dataset (Boyce & Levy, 2023) contains crowd-sourced Maze task responses from 95 participants to the same materials as in the Natural Stories SPR dataset above, using a recently developed technique (A-Maze) to generate high quality forced-choice alternatives for long naturalistic passages (Boyce et al., 2020). The data can be accessed online at https://github.com/vboyce/amaze-natural-stories.

The dataset contains a total of 97,527 responses (the textual statistics are the same as Natural Stories SPR above). Following Boyce and Levy (2023), RTs are removed if they are less than 100 ms or greater than 5,000 ms, if they are to words adjacent to a sentence boundary, or if the subject responded incorrectly (i.e., selected the wrong continuation). Inattentive subjects (defined as subjects with lower than 80% accuracy) are also removed.

#### Provo ET.

The Provo ET dataset (Luke & Christianson, 2018) contains eye-tracking data from 84 participants who read 55 short (39–62 word) passages from various online sources on a computer monitor. The data can be accessed online at https://osf.io/sjefs/.

The dataset contains a total of 134 sentences, 2,745 words, and 213,224 distinct fixations to word regions on the screen. Following the Dundee protocol (above), responses are filtered to exclude fixations following large outlier saccades (>20 words in either direction), fixations to words adjacent to a sentence boundary (screen and line boundaries were not annotated), and fixations interrupted by blinks.

### Critical Predictors

Broad-coverage estimates of words’ predictability in context across the naturalistic stimuli considered here are computed using the default GPT-2(-small) model (Radford et al., 2019) provided by the HuggingFace library (Wolf et al., 2020). GPT-2 is a 175 M parameter deep neural network language model that computes a nonlinear transformation of long contexts (up to 1024 preceding words) using the Transformer architecture (Vaswani et al., 2017) to generate a probability distribution over the next word. GPT-2 has been shown to correlate strongly with human measures of language processing, both behavioral (Wilcox et al., 2020) and neural (Schrimpf et al., 2021), and GPT-2 surprisal outperforms cloze estimates as a predictor of human reading latencies (Shain et al., in press). Based on strong prior evidence that predictability effects in reading are at least logarithmic (Hoover et al., 2023; Shain et al., in press; Smith & Levy, 2013; Wilcox et al., 2020, 2023; though cf. Brothers & Kuperberg, 2021), predictability is represented on a surprisal (negative log probability) scale. GPT-2 sometimes breaks words into subtokens, but word-level surprisals are recovered by applying the chain rule (i.e., summing subtoken surprisals within the word). GPT-2-small is chosen over larger variants of GPT-2 (e.g., GPT-2-XL) or larger variants of later GPT models (e.g., GPT-3; Brown et al., 2020) based on evidence that GPT-2-small provides substantially better fit to reading times than these other models (Kuribayashi et al., 2023; Oh et al., 2021; Shain et al., in press). In particular, although numerous more advanced language models have been released since GPT-2-small, these models are ubiquitously *instruction-tuned* (e.g., by reinforcement learning with human feedback) in order to improve their alignment with the preferences of human chatbot users. Instruction tuning may therefore compromise the interpretation of language models as models of next-word predictability, and recent evidence suggests that it frequently degrades the alignment between surprisal and human reading times (Kuribayashi et al., 2023). Among models trained strictly as language models (without instruction tuning), the GPT-3-davinci-002 model remains as of this writing the most advanced in the GPT family and among the most performant available, and prior work indicates that it substantially underperforms GPT-2-small as a model of human reading times (Shain et al., in press). Thus, available psychometric evidence favors GPT-2-small for modeling human reading behavior (even if larger models perform better for other measures, such as brain activity, e.g., Antonello et al., 2023; Schrimpf et al., 2021). Further details about the procedure used to compute surprisal from the GPT-2 model can be found in Shain et al. (in press).

Although the precise training dataset used by GPT-2 is proprietary, it is known that its training data is much larger than datasets typically used to estimate word frequency in psycholinguistics (e.g., CELEX, estimated from 18 M words; Baayen et al., 1995). To minimize effects of training size, word frequencies are therefore estimated from OpenWebText (Gokaslan & Cohen, 2019), a 6.5 B-word open replication of GPT-2’s WebText training corpus. To facilitate comparison with surprisal above, frequency effects are represented on a *unigram surprisal* scale, that is, the negative log prior probability of the word. This measure is equivalent (modulo an additive constant) to the negation of the log frequency scale regularly used in psycholinguistics (Baayen & Lieber, 1996; Baayen et al., 2016; Balota & Chumbley, 1984; Carrol, 1967; Demberg & Keller, 2008; Norris, 2006; Rumelhart & Siple, 1974). Unigram surprisals are estimated using KenLM (Heafield et al., 2013) with default hyperparameters.

In addition to the critical predictability and frequency predictors above, all models contain a number of control predictors, described in SI A.1. All models include full by-participant random intercepts, slopes, and neural network parameters, as well as random intercepts by token position in each text.

### Analysis

#### Continuous-Time Deconvolutional Regression.

All analyses use continuous-time deconvolutional regressive neural networks (CDRNNs; Shain, 2021; Shain & Schuler, in press). In brief, CDRNNs use artificial neural networks to estimate a nonlinear function relating a stimulus event (i.e., a fixated word with its attendant features) to its effect on the parameters of the predictive distribution over the response (here, the location, dispersion, and skewness parameters of the exGaussian distribution) at some continuous delay. Unlike standard approaches to time series regression like linear mixed-effects models (LMEMs; Bates et al., 2015) and generalized additive models (GAMs; Wood, 2006), CDRNNs simultaneously relax assumptions that the IRF is discrete-time, linear, stationary (time-invariant), and heteroscedastic (constant error), all of which are implausible in the context of naturalistic reading (Shain & Schuler, in press). Full description of the CDRNN approach can be found in Shain and Schuler (in press). CDRNN implementation details used in this study are described in SI A.

#### Statistical Procedure.

Statistical testing within this continuous-time deconvolutional framework relies on out-of-sample model comparison: models instantiating the null vs. alternative hypotheses are trained on a portion of the data (training set), and conditional likelihoods from these models on an unseen portion of the data (test set) are statistically compared in order to determine whether the model instantiating the alternative hypothesis generalizes better than the model instantiating the null hypothesis (Shain & Schuler, 2021). All tests aggregate over ensembles of 10 models, which reduces variability due to stochastic optimization. Following Shain et al. (in press), ensembles are compared using paired permutation tests of out-of-sample conditional likelihood. Full details of the testing protocol are described in SI A.

In this study, the main model is a CDRNN in which all frequency, predictability, and control predictors are permitted to interact and to influence all parameters of the exGaussian predictive distribution. This model is used for all visualizations. Null models testing individual effects or interactions are constructed as follows.

To test overall frequency or predictability effects, null variants of the main model were fitted with frequency and/or predictability ablated. To test an overall frequency-predictability interaction, a null model is constructed containing two distinct neural networks, one convolving unigram surprisal and all control predictors, and another convolving GPT-2 surprisal and all control predictors. This design allows unigram surprisal and GPT-2 surprisal to interact with control variables, but not with each other. To ensure a matched architecture for fair comparison, the alternative model also contains two (redundant) neural networks, with each network convolving all predictors. To test effects of frequency or predictability on specific distributional parameters, models are constructed containing three distinct neural networks, one for each of the three distributional parameters (location, dispersion, and skewness). Null models involve an ablation of a predictor from one of these networks, and alternative models include all predictors in all networks. For example, to test unigram effects on skewness, unigram surprisal is removed from the neural network that generates the skewness parameter, holding everything else constant. For additional details and justification, see SI A.2.

## RESULTS

**Figure 1.**
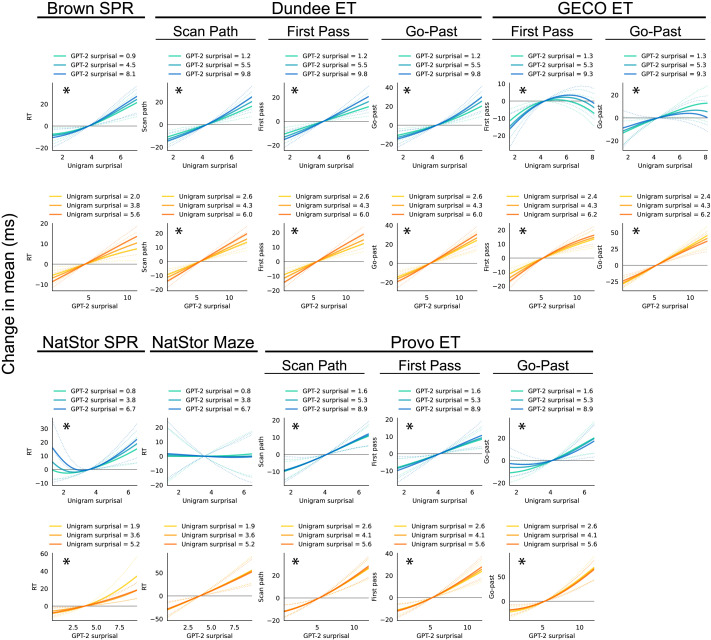
Estimated effects of frequency (Unigram surprisal) and predictability (GPT-2 surprisal) on mean word-by-word reading time across datasets. Plots show how the instantaneous response (i.e., at no delay) deviates from its mean as a function of one predictor (*x* axis) at three different values (line colors) of the other predictor (mean, ±1 standard deviation), thus revealing how the frequency effect changes as a function of predictability (and *vice versa*). Dotted lines show 95% variational Bayesian credible intervals. Plots showing effects that make a significant unique contribution to generalization likelihood are marked with *. In all datasets, effects of frequency are similar across the range of predictability values and *vice versa*, supporting a lack of interaction (i.e., the two predictors modulate reading times mostly independently; interactions are not significant overall or in any but 1/11 comparisons, see Tables S2–S6). The individual parameters of the exGaussian distribution show a similar pattern (SI H). More detailed visualizations of these effects over time are given in Figures S1–S4, and visualizations that also include the control predictors are given in Figure S6.

The key CDRNN model estimates are shown in Figure 1 as plots representing the expected influence of frequency and predictability on mean reading latency in milliseconds (ms). Because CDRNN models simultaneously estimate a nonlinear multivariate response to all predictors over time, visualizations show average case estimates of the instantaneous effects (i.e., at no delay) of predictor values on the response, holding all other model inputs at their expected values (for details, see SI B). Detailed visualizations of the estimated response over time for both the mean response and all parameters of the exGaussian distribution are provided in SI C and D. The CDRNN models generalize well across datasets (SI E), and similar effects appear in model-free visualizations (SI F). Detailed results of all statistical tests in each dataset, as well as aggregate tests across all datasets, are given in SI G.

### Are Frequency and Predictability Effects Dissociable in Naturalistic Reading?

As shown in Figure 1, CDRNN models find generalized positive effects for both frequency (unigram surprisal) and predictability (GPT-2 surprisal) on reading time. These effects contribute significantly to test set likelihood (in aggregate tests, as well as in all individual comparisons for GPT-2 surprisal and in 10/11 individual comparisons for unigram surprisal). Moreover, these effects are dissociable: unigram surprisal is significant over GPT-2 surprisal alone (both in aggregate and in 10/11 individual comparisons), and GPT-2 surprisal is significant over unigram surprisal alone (both in aggregate and in 9/11 comparisons). Thus, neither effect is reducible to the other, consistent with the procedural view of frequency effects and contrary to inferential view.

### Do Frequency and Predictability Interact?

Results show little evidence that frequency and predictability interact: the estimated effect of either variable (Unigram or GPT-2 surprisal) in Figure 1 changes little as a function of the other, supporting an additive relationship. Moreover, the interaction is not significant in aggregate tests, and is significant in only 1/11 individual comparisons (and the estimated interaction for the one exception—Provo scan path pass durations—is faint). This result supports additive contributions of these two effects, consistent with the procedural view of frequency effects and contrary to the inferential view. Because interactions do not emerge in overall reading times, interactions in individual distributional parameters (location, dispersion, and skewness) are not tested (the visual estimates in Figures S10–S12 generally suggest an absence of interactions at the parameter level as well).

### Do Frequency and Predictability Have Differential Effects on the Distribution Over Reading Times?

The distributional findings (isolated effects on location, dispersion, and skewness parameters) do not support a systematic dissociation in the contributions of frequency (unigram surprisal) vs. predictability (GPT-2 surprisal) to different distributional parameters. In fact, no single distributional effect is significant in aggregate tests (Table S6), although effects on the skewness parameter are significant in the majority of individual comparisons for both unigram surprisal (6/11 comparisons) and GPT-2 surprisal (7/11 comparisons). The failure of effects on the skewness parameter to reach significance in aggregate tests appears to be largely due to the Natural Stories SPR dataset, in which modeling frequency and predictability effects on distributional parameters did not improve generalization likelihood. Thus, although Natural Stories SPR provides strong evidence of overall effects of frequency and predictability, it appears to be ambivalent as to their distributional source, with clearer distributional patterns emerging in other datasets.

By contrast, the location parameter *μ* and the dispersion parameter *σ* show little evidence of generalized modulation by either frequency or predictability. No effect on the location parameter is significant in aggregate tests, and effects on location are significant in only a small number of individual comparisons (1/11 comparisons each for both unigram and GPT-2 surprisal). Effects on the dispersion parameter *σ* are likewise weak (non-significant overall, and significant in only 1/11 comparisons each for both unigram surprisal GPT-2 surprisal). Supplementary visualizations of model-estimated frequency and predictability effects on these three distributional parameters (SI H) converge with this general impression. For both unigram and GPT-2 surprisal, the overall effects appear to be primarily driven by a modulation of the skewness parameter *τ*, whose response profile (Figure S12) is similar in shape to that of the overall mean (Figure 1; note that the mean of the exGaussian distribution is linear in its skewness parameter). By contrast, effects on the location parameter *μ* (Figure S10) and the dispersion parameter *σ* (Figure S11) are generally estimated to be weak, with high uncertainty. Thus the distributional results support similar contributions to reading latency from both word frequency and word predictability: both variables primarily drive the skewness (*τ*) parameter.

### Do Frequency and Predictability Effects Differ in Their Timecourses?

Given this evidence for dissociable frequency and predictability effects, one possible recourse for the inferential view could be to posit delays in the timecourse with which contextual information becomes available. In particular, if contextual information becomes available more slowly than lexical information via sensory cues, then the predictive processor may initially rely only or primarily on frequency information, and only later revise its expectations as context is taken into account. This account predicts the frequency-predictability dissociation attested above, but additionally makes the prediction that there should be temporal asymmetries: frequency effects should register primarily in earlier measures of processing and peak relatively quickly (since contextual information comes online to displace the base frequency in conditioning the probability distribution), whereas predictability effects should register primarily in later measures of processing and peak relatively slowly (since it takes time to bring context to bear on the inference process).

**Figure 2.**
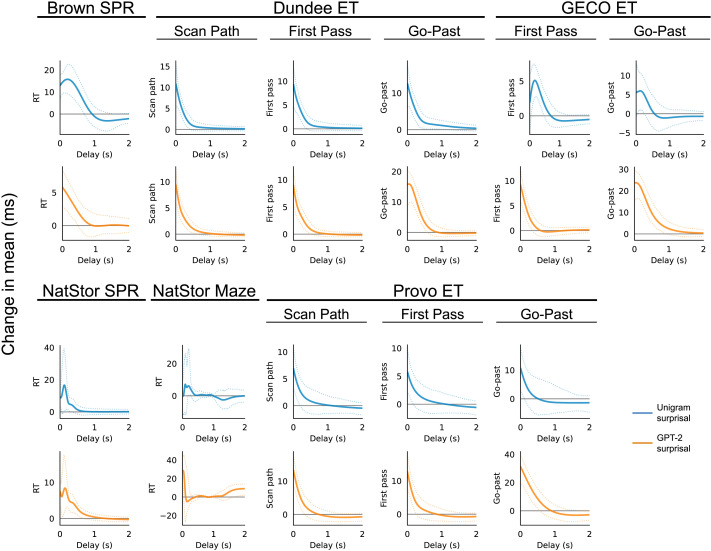
Estimated timecourses of frequency and predictability effects on mean word-by-word reading time across datasets. Plots show the expected change in response (*y* axis) from observing an increase above the mean of one standard deviation of unigram surprisal (frequency) or GPT-2 surprisal (predictability) as a function of delay (*x* axis) from initial fixation, from 0 s (the immediate effect on the current word) to 2 s (the effect on a word fixated 2 s in the future). The plots thus represent a continuous-time version of the “spillover” effects commonly included in word-by-word psycholinguistic analyses. More detailed visualizations of effects over time on all three distributional parameters (*μ*, *σ*, and *τ*) over a continuous range of frequency and predictability values are given in Figures S1–S4.

CDRNN models directly estimate effect timecourses and can therefore be used to address this question. Visualizations of these timecourse estimates are provided in Figure 2, which shows little evidence for either of the predictions above. Instead of quickly-peaking frequency effects and slowly peaking predictability effects, frequency and predictability effects are both primarily concentrated on the current word and decay at a similar, roughly monotonic rate lasting about 500 ms–1000 ms depending on the response measure, including in both early (scan path) and late (go-past) durations in eye-tracking data and in slower self-paced experiments (Natural Stories SPR) in which the measure depends on a manual motor response (button pressing). Likewise, both frequency and predictability exhibit similar effect timecourses within a given experiment, with no visual indication that frequency effects have faster dynamics than predictability effects. Instead, frequency and predictability effects arise (and dissociate) ubiquitously across online measures of comprehension difficulty, with qualitatively similar dynamics of influence on reading behavior.

### Do Frequency Effects Reflect Lexical Access or Lossy Memory for Context?

Although results so far support the frequency-predictability dissocation predicted by the procedural view, rather than the unity predicted by the inferential view, they are nonetheless consistent with recently-developed *resource-rational* variants of the inferential view (Futrell et al., 2020; Hahn et al., 2022), also known as “lossy-context surprisal”, in which past context is also a latent variable over which the comprehender must perform inference, due to imperfect memory for preceding input. Thus, according to the resource-rational view, human comprehenders condition their predictions on an uncertain representation of context, whereas computational language models like GPT-2 condition their predictions on a veridical representation of context. This difference could give rise to apparent frequency effects, since frequency information can serve as a form of error correction on surprisal from veridical language models, which might over-rely on context relative to humans (Goodkind & Bicknell, 2021).

Although the resource-rational version of the inferential view makes the same empirical prediction about the frequency-predictability relationship as the procedural view, the two views diverge in their predictions about the effect of context length. In particular, the resource-rational view construes frequency and (model-derived) predictability effects as merely imperfect approximations to the same underlying process (lossy-context surprisal) that respectively underestimate and overestimate the amount of context available to humans. Thus, the resource-rational view predicts additional contributions of other predictability measures along the continuum from no context to full context (e.g., bigram or trigram predictability), since such measures should incrementally improve the approximation to the true predictability function. By contrast, the procedural view construes frequency and predictability as reflecting qualitatively different processes (lexical retrieval and prediction respectively) and therefore does not predict an additional contribution from e.g., bigram or trigram estimates of predictability. The question then becomes whether predictability estimates derived from intermediate context lengths improve model fit, as e.g., Goodkind and Bicknell (2021) have argued based on the Dundee dataset.

**Figure 3.**
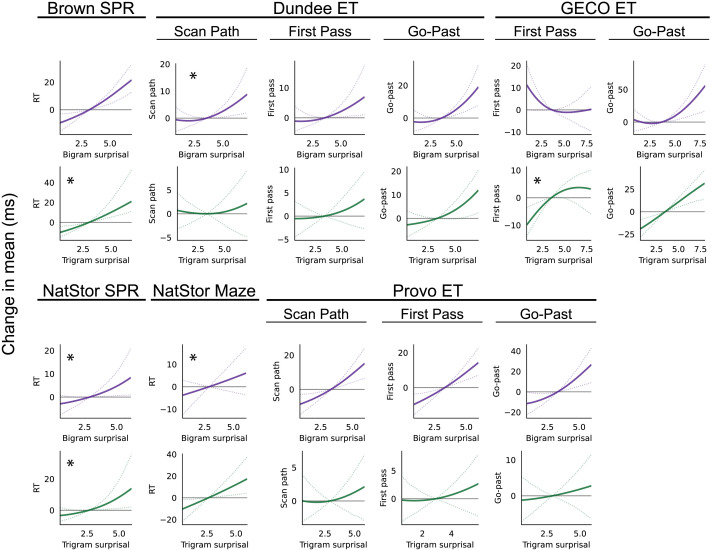
Model estimates across datasets of the expected influence of bigram and trigram surprisal on reading times. Plots show the estimated instantaneous change (i.e., at a delay of 0 s) in the response (with 95% variational credible intervals) as a function of bigram and trigram surprisal. Estimates of bigram and trigram effects over time are plotted in Figure S5. Plots showing effects that make a significant unique contribution to generalization likelihood are marked with *.

To answer this question, KenLM bigram and trigram surprisals are estimated (using the same procedure as for the unigram surprisal estimates) and added to the CDRNN models for each dataset. Resulting estimates are plotted in Figure 3. As shown, across datasets, both bigram and trigram surprisal are systematically associated with numerical increases in reading time even in the presence of unigram and GPT-2 surprisal estimates. These effects do not significantly improve model fit for most individual datasets, but the bigram effects emerge as significant in overall comparisons across datasets. The consistency of positive estimates across datasets and the significance of bigram effects in overall comparisons are evidence (albeit somewhat weak given the lack of confirmation in most individual datasets) that bigram models capture patterns of reading behavior that are not fully captured either by frequency or full-context GPT-2 predictability. The bigram estimate is also not merely a better predictor than unigram or GPT-2 surprisal: in aggregate tests, both unigram and GPT-2 surprisal contribute significantly to model fit over bigram surprisal. Thus, unigram, bigram, and GPT-2 surprisal each contribute uniquely to generalization likelihood. Although this finding does not contradict the procedural view’s framing of frequency effects as reflecting lexical retrieval, it is also not a principled prediction of (and therefore not explained by) the procedural view. By contrast, this outcome is a clear prediction of the resource-rational inferential view, according to which frequency effects stand at one pole in a continuum of approximation (which also includes bigram effects) to the effects of lossy memory on human subjective surprisal. Results thus offer some reason to favor a resource-rational interpretation of the frequency-predictability dissociation found in this and other studies, although follow-up work (e.g., implementing lossy-context surprisal as in Hahn et al., 2022) is needed to evaluate this interpretation more directly.

## DISCUSSION

This work revisits a longstanding question about the mental computations involved in human language comprehension and the degree to which they are sensitive to context: do word frequency and word predictability exert dissociable effects on reading behavior? Such a dissociation is predicted by a *procedural* view of language comprehension which construes processing as an intricate coordination of distinct cognitive operations, including lexical retrieval (with difficulty indexed by lexical frequency) and next-word prediction (with difficulty indexed by predictability). However, an alternative *inferential* view of language comprehension construes processing as probabilistic inference over possible meanings given evidence (strings of words) and predicts that there should be no independent effect of word frequency once word predictability is taken into account. Prior controlled comparisons between frequency and predictability have generally reported the dissociation predicted by the procedural view—both on mean reading time (e.g., Altarriba et al., 1996; Ashby et al., 2005; Hand et al., 2010; Kretzschmar et al., 2015) and on the distribution over reading times (Staub, 2011; Staub et al., 2010)—and have thus been taken to support lexical retrieval and prediction as distinct cognitive operations. However, this pattern has yet to be convincingly validated in naturalistic reading, where mixed results have emerged (Goodkind & Bicknell, 2021; Shain, 2019) using statistical models with implicit simplifying assumptions that are known to be problematic for the reading domain (Shain & Schuler, in press).

The contribution of the present study is to improve both the scale and modeling sophistication of frequency and predictability effects in naturalistic reading. To this end, analyses use flexible time series analysis (Shain & Schuler, in press) and high-performance frequency (Gokaslan & Cohen, 2019) and predictability (Radford et al., 2019) estimates to quantify reading patterns from six large-scale publicly available reading datasets, yielding a combined total of over 2.2 M fixation events collected by diverse research groups. This approach enables (i) discovery of arbitrarily complex frequency-predictability interactions, (ii) stringent evaluation based on generalization to unseen data, and (iii) analysis of effects on the full distribution over reading times, not just their mean. Despite this flexibility and scale, the pattern that emerges is both simple and strikingly consistent with both prior controlled experiments and the predictions of the procedural view of language comprehension: word frequency and word predictability strongly dissociate in naturalistic reading and contribute additively to the time participants take to process words. This result stands in contrast to Shain (2019), who failed to find a frequency-predictability dissociation in a subset of dataset used in the present study. However, as acknowledged in Shain (2019), this lack of dissociation was based on a null result (despite positive frequency estimates, frequency did not statistically improve model fit over predictability) and did not fully conform to the predictions of the inferential view (in the Dundee dataset, predictability also failed to improve over frequency, resulting in an ambiguity as to which effect was primary). The present study improves upon the Shain (2019) approach in almost every way (more datasets, fewer assumptions, more powerful statistical models, more thorough evaluations) and should therefore be considered more trustworthy. For detailed discussion of the relationship between the present study and Shain (2019), see SI I. Results also clarify the nature of this dissociation: despite prior claims to the contrary (Staub, 2011, 2015; Staub et al., 2010), the present study provides no evidence that frequency and predictability differentially affect the parameters of an exGaussian distribution over reading times. Instead, both frequency and predictability modulate the distribution in qualitatively similar ways (primarily by modulating the skewness of the distribution, rather than its location or scale). The frequency-predictability dissociation is furthermore not explained by temporal asymmetries in the availability of lexical vs. contextual information: frequency and predictability dissociate across both fast (scan path durations in eye-tracking) and slow (self-paced reading) experimental modalities, and frequency and predictability show qualitatively similar dynamics of influence on reading behavior. The existence of frequency effects therefore does not appear to be explained by delayed access to contextual information.

On one interpretation, these findings support a procedural view of frequency effects as driven by lexical retrieval operations that are independent of predictive processing. Under this interpretation, retrieval processes experience difficulty in proportion to the strength of memory encoding in the mental lexicon, which is assumed to be well approximated by (log) frequency (Staub, 2015). As stressed in the Introduction, this result does not necessarily favor wholesale adoption of the procedural view that variation in word-by-word processing demand is driven primarily by representation-building rather than probabilistic inference. Indeed, considerable evidence has accumulated that probabilistic inference over sentence interpretations is a major component of language comprehension and is primarily responsible for effects of predictability on processing demand (Hoover et al., 2023; Meister et al., 2021; Shain et al., in press; Smith & Levy, 2013; Szewczyk & Federmeier, 2022; Wilcox et al., 2020, 2023). The frequency-predictability dissociation reported this this study may therefore highlight an important determinant of processing demand that is not explained by inference over sentence interpretations, but is consistent with distinct processes of memory retrieval. In other words, although prior evidence favors an inferential (rather than a procedural preactivation-based) interpretation of predictability effects and thus implicates inference as a key “causal bottleneck” on processing demand, the present finding of surprisal-independent frequency effects could suggest limits on the scope of this bottleneck: frequency (and thus plausibly lexical retrieval) also plays a large and surprisal-independent role in determining how long participants spend reading words. Given the remarkable success of surprisal in accounting for a range of language processing phenomena across diverse experimental measures (Demberg & Keller, 2008; Frank & Bod, 2011; Frank et al., 2015; Heilbron et al., 2022; Hoover et al., 2023; Lopopolo et al., 2017; Roark et al., 2009; Shain et al., 2020, in press; Smith & Levy, 2013; van Schijndel & Schuler, 2015; Wilcox et al., 2020), discoveries highlighting the explanatory limits of surprisal offer opportunities for new insights into the mechanisms and representational format of incremental meaning construction during language comprehension (e.g., Huang et al., 2023; van Schijndel & Linzen, 2021).

Nonetheless, retrieval difficulty is not the only possible interpretation of dissociable frequency and predictability effects in reading. An alternative interpretation draws on the notion of *resource-rationality* to revise the inferential view in such a way that it also predicts the existence of prediction-independent frequency effects. In particular, under the assumption that humans predict based on imperfect memory of the context, the resource-rational perspective predicts that frequency effects will emerge as a form of error correction on predictability estimates (like GPT-2 surprisal) that use veridical context representations (and thus overestimate the influence of context on human predictions). Note that this prediction holds regardless of whether predictability is estimated using computational language models (as was done here) or human cloze experiments (in which participants’ next-word predictions given a context are repeatedly sampled to construct an empirical distribution; Taylor, 1953), as in most constructed experiments that have also shown frequency-predictability dissociations (e.g., Altarriba et al., 1996; Ashby et al., 2005; Hand et al., 2010; Kretzschmar et al., 2015). This is because cloze is typically an offline task in which participants have full access to the written context when making predictions, thus removing the bottlenecks imposed by lossy memory during online processing. Under the resource-rational inferential view, it is therefore misguided to search for frequency-predictability dissociations in the first place, since such dissociations are ambiguous between (i) distinct cognitive processes for retrieval and prediction and (ii) one errorfully-estimated prediction process. Instead, the resource-rational inferential view motivates a focus on the additional prediction (not made by the procedural view) that intermediate forms of context truncation (e.g., bigram and trigram predictability) will further refine the approximation of human subjective surprisal and thus contribute to fit to reading times over frequency and full-context predictability alone. The present study provides tentative support for this interpretation by showing that bigram and trigram predictability are positively associated with reading time over and above frequency and GPT-2 predictability, reaching significance for the case of bigrams in overall comparisons. Nevertheless, to convincingly substantiate this interpretation, investigation is needed using computational models designed to directly simulate prediction from lossy memory, which according the resource-rational inferential view should entirely explain both frequency and (full-context) predictability effects (e.g., Hahn et al., 2022).

Results also bear on the role of frequency and predictability in shaping the distribution over reading times. Prior work has established that the exGaussian distribution is a strong descriptive model of reaction times in general (Balota & Yap, 2011; Heathcote et al., 1991; Hohle, 1965; Staub et al., 2010)—and of naturalistic reading specifically (Shain et al., in press)—relative to the normal distribution. The present study finds the strongest evidence of frequency and predictability effects on the skewness parameter of the exGuassian distribution, with no evidence that either frequency or predictability systematically influence either the location or dispersion of the distribution. In other words, low frequency and low predictability words increase mean reading time primarily by pushing probability mass into the tail (thus favoring longer reading times), rather than by shifting the probability distribution toward longer reading times. There is thus no evidence in the present study for a prior claim that frequency and predictability dissociate not only with respect to mean reading time but also with respect to which parameters of the exGaussian distribution they act on (Staub, 2011, 2015; Staub et al., 2010); instead, frequency and predictability appear to influence the distribution over reading times in qualitatively similar ways, at least during naturalistic reading. The implications of this result for cognitive processes are not yet clear given current debates about the theoretical interpretation of the exGaussian distribution: the classical interpretation of the skewness and location parameters as respectively reflecting perceptual and motor processes (Hohle, 1965) is now in question (Matzke & Wagenmakers, 2009). Nonetheless, this distributional finding provides a bridge to the reaction time literature whereby future refinements to our understanding of the cognitive underpinnings of reaction time distributions may transfer directly to our understanding of sentence processing.

The present study focuses on frequency and predictability effects on reading behavior, but there are two related lines of prior research that bear mentioning in this context. *First*, studies of lexical decision times (i.e., the time taken for participants to decide whether a stimulus is a real word in their language) that use single-word prime contexts tend to find frequency-priming interactions whereby priming effects are larger for low frequency words (Becker, 1979; Borowsky & Besner, 1993; Forster & Davis, 1984; Norris, 1984). As a contextual effect, priming is conceptually similar to prediction and under certain assumptions (i.e., prediction as preactivation-based facilitation, Brothers & Kuperberg, 2021) may operate on the same cognitive mechanisms (lexical activation levels). However, this is not the case under the inferential view of predictability effects assumed here (Hale, 2001; Levy, 2008) based on prior evidence (Hoover et al., 2023; Shain et al., in press; Smith & Levy, 2013; Wilcox et al., 2020, 2023), whereby predictability effects reflect the difficulty of updating inferences over sentence interpretations. On this view, single-word priming effects in lexical decision are less relevant to predictability: not only is there no necessary influence of priming on the probability assigned to sentence interpretations, but there are no sentences to interpret in this task at all. Thus, in order for priming effects in lexical decision to challenge the key claims of this study, assumptions must be made about the mechanisms that underlie predictability effects, and these assumptions are at odds with a substantial body of evidence. By the same token, the fact that priming effects arise in non-sentential tasks like lexical decision is difficult for an inferential account to explain (since, again, there are no sentence interpretations over which to perform inference). Thus, the present finding of prediction-independent frequency effects in reading aligns with the priming literature in lexical decision to identify plausible signatures of activation-based memory processes (frequency, priming) that can be teased apart from inferential processes. The relationship between prediction and priming is thus an important open question (Metusalem et al., 2012) that may further illuminate the computations that enable human language comprehension.

*Second*, studies of word frequency and predictability effects in ERPs during sentence processing tend to find frequency-predictability interactions whereby predictability effects are stronger for low vs. high frequency words (Dambacher et al., 2006; Sereno et al., 2003, 2020; Van Petten & Kutas, 1990), unlike similar studies using eye-tracking (Staub, 2015). The reasons for this apparent discrepancy between eye-tracking and ERP results are not well understood and cannot be satisfactorily resolved here. However, as discussed in the Introduction, under the view advocated here that frequency effects are additive with *surprisal scale* predictability effects, empirical predictions about the presence or absence of interactions in factorial experiments (i.e., experiments that cross high and low predictability with high and low frequency) are murky. This is because surprisal is much harder to match within low-predictability vs. high-predictability conditions (due to its logarithmic scaling), potentially giving rise to differences in the level of confounding between frequency and surprisal between these two conditions in a given experiment. These differences may in turn vary in degree between experiments as a function of their design, which could drive variation between experiments as to whether frequency-predictability interactions emerge as significant. Importantly, the word-by-word regression modeling used in the present study simultaneously sidesteps this interpretational issue and simplifies the empirical question: do surprisal-independent frequency effects exist in naturalistic reading? Results support an affirmative answer.

In conclusion, although additional work is needed to adjudicate between interpretations of the frequency-predictability dissociation, one thing is clear from these results: frequency effects are not explained by surprisal theory in its standard form, even during continuous naturalistic reading. We therefore now have a striking accumulation of evidence for this conclusion from both experimental and naturalistic reading studies (Altarriba et al., 1996; Ashby et al., 2005; Bélanger & Rayner, 2013; Gollan et al., 2011; Goodkind & Bicknell, 2021; Hand et al., 2010; Kretzschmar et al., 2015; Lavigne et al., 2000; Miellet et al., 2007; Rayner et al., 2001, 2004, this work). The frequency-predictability dissociation is thus a multiply-replicated empirical pattern that should be treated as a key explanandum in theories of language comprehension; such signposts are rare in cognitive science and essential for theoretical progress. The frequency-predictability dissociation also helpfully eliminates an *a priori* plausible region of the hypothesis space, namely, that frequency effects are merely a species of predictability effects under standard assumptions (veridical context) of the inferential view. This hypothesis is not tenable in the face of these results, and additional theoretical commitments about the inferential process (e.g., lossy context memory) are needed in order to bring the inferential view into conformity with the evidence. The findings of this work thus narrow the space of plausible theories of language comprehension, and suggest clear paths toward further theoretical refinement.

## ACKNOWLEDGMENTS

This research was supported by a postdoctoral fellowship from the Simons Center for the Social Brain. Thanks to two anonymous reviewers, as well as Roger Levy, Evelina Fedorenko, William Schuler, and Edward Gibson, for invaluable feedback on the initial drafts.

## FUNDING INFORMATION

C.S. was supported by a postdoctoral fellowship from the Simons Center for the Social Brain at MIT (via the Simons Foundation).

## DATA AVAILABILITY STATEMENT

All data and code are publicly available. Data: https://osf.io/8v5qb/. Code: https://github.com/coryshain/cdr.

## Supplementary Material


