# Supplementary material for: Word Frequency and Predictability Dissociate in Naturalistic Reading
Source: Open Mind (Camb). 2024 Mar 5;8:177–201. doi: 10.1162/opmi_a_00119 (PMC10932590; doi:10.1162/opmi_a_00119)
Supplement: Supplementary file 1 [file opmi-08-177-s001.pdf]

# Supplementary Information for *Word Frequency and Predictability Dissociate in Naturalistic Reading*

## A CDRNN implementation and statistical procedure

Unless otherwise indicated, all CDRNNs were implemented as described in Shain and Schuler (2022) with the following parameter settings:

- Feedforward IRF with two hidden layers of 32 units each.
- Full random effects (zero-centered deviations in the model intercepts, linear coefficients, and layerwise bias terms) by subject and random intercepts by token.
- Dropout rate (Srivastava et al., 2014) of 0.1 on (a) all hidden layers and (b) random grouping factor variables.
- L2 weight regularization constant of 5.
- L2 random effects regularization constant of 10.
- To speed convergence, prior to fitting, response variables are z-scored and predictor variables are rescaled by their standard deviations.
- Fixed intercepts and coefficients assume a standard normal prior, and, following Shain and Schuler (2021), random intercepts and coefficients assume a normal prior with mean 0 and standard deviation 0.1. Variational posteriors over these parameters are estimated using variational expectation maximization.
- For computational efficiency, histories are truncated at 32 words or 60s into the past, whichever is shorter.
- Convergence is diagnosed based on a time-loss correlation criterion, where the loss is the validation set likelihood evaluated every 10 epochs and the correlation is computed over a window of 250 consecutive epochs. Thus, convergence is declared whenever the validation set likelihood is statistically non-increasing at $\alpha = 0.5$ for at least 13 of the preceding 25 evaluations. For full details about this procedure, see Shain and Schuler (2021). Following convergence, the model state with the best validation set performance is used for all evaluation and visualization.

Full code and model configuration files needed for reproduction are provided at `https://github.com/coryshain/cdr`.

The validation set is used to define an early stopping criterion for model training: validation set likelihood was assessed every 10 training epochs, and convergence is diagnosed whenever a sequence of 10 of these evaluation checkpoints was statistically uncorrelated with training time, following the statistical testing procedure defined in Shain and Schuler (2021). Following convergence, the training state corresponding to the maximum validation performance is selected as the final state. Hypotheses are statistically evaluated on the test set, with separate tests for each response variable (scan path, first pass, and go-past durations in Dundee and reading time in Natural Stories).

## A.1 Control predictors

We included the following control predictors in all models:

- **Rate.** A "deconvolutional intercept" (Shain and Schuler, 2021) describing the average response to a word, independent of its properties. *Rate* is so named because its influence on the response depends solely on stimulus timing.
- **Word length.** The length of the word (in characters).
- **End of sentence.** Whether a word ends a sentence (binary indicator), designed to capture diffuse effects of sentence boundaries (e.g., Breen, 2014; Nelson et al., 2017), even though the final words of sentences themselves are excluded from analysis (see above).

Models of eye-tracking datasets additionally contained the following control predictors that are specifically relevant to the eye-tracking modality:

- **Saccade length.** Length in words of incoming saccade (eye movement).
- **Regression.** Whether the fixation is part of a regressive (backward) eye movement (binary indicator).

Since the Dundee corpus additionally provides annotations for screen and line boundaries, we included these as regressors in Dundee models only:

- **End of line.** Whether a word ends a line of text on the display (binary indicator).
- **End of screen.** Whether a word ends a screen on the display (binary indicator).

Finally, the Maze task used in Natural Stories Maze involves a potentially errorful word-by-word forced choice task. Therefore, for this dataset alone, we modeled the possibility of effects from task errors using the following regressor:

- **Incorrect.** Whether the incorrect continuation was chosen in the A-Maze task (binary indicator).

The CDRNN models used here flexibly capture interactions between any combinations of these variables (Shain and Schuler, 2022). Thus, by including e.g., the *regression* predictor, the models can learn not only overall differences in response to regressive vs. non-regressive fixations, but also e.g., differences in surprisal or word length effects between regressive vs. non-regressive fixations. This ability is important because fixations during regressive eye movements plausibly differ in their processing demands, since they involve material that was likely already viewed either foveally or parafoveally.

## A.2 Null models

A CDRNN model that takes the full word-by-word predictor vector as input and generates a parameterization of the predictive distribution as output simultaneously models the influence of frequency and predictability (along with their interactions both with each other and with the control variables) on all parameters of the predictive distribution. However, for the purposes of ablative hypothesis testing of the kind performed here, it is important to be able to isolate the unique contributions of specific predictors or interactions. Thus, the nature of the hypothesis being tested guides the definition of the null model used in comparisons. This work uses three distinct types of null models corresponding to the three classes of research question at hand. These null models are described and motivated at a high level below; for detailed model formulae, see **SI L**.

### A.2.1 No overall effect

One class of question concerns whether a predictor (e.g., frequency or predictability) exerts an effect of any kind on the response. The null model for this kind of question is simply one in which the critical predictor is removed, but the rest of the model specification remains the same.

### A.2.2 No interaction

A second class of question concerns whether two predictors interact in their effects on the response. Selectively ablating a specific interaction in a neural network (while preserving all other interactions) is challenging because the network defines a nonlinear function over the entire predictor space. To address this limitation, null models for tests of the interaction of predictor A and predictor B define the impulse response function by summing the outputs of two subnetworks: one that applies to predictor A and all control variables, and another that applies to predictor B and all control variables. This ensures that predictors A and B can interact arbitrarily with all other variables in the model, but not with each other. The same architecture is enforced on the alternative model in these comparisons. In other words, in the alternative model, both subnetworks have the same inputs (A, B, and all other variables), resulting in an IRF that is the sum of two feedforward neural networks with the same inputs. This (redundant) architecture for the alternative model makes little sense on its own, but has in principle the same solution space as a single-network model, and it is needed in this case for fair comparison to the null model (otherwise, differences could be attributable to architectural differences, in addition to any differences due to the presence or absence of an interaction).

### A.2.3 No effect on a specific distributional parameter

A third class of questions concerns whether a predictor has a unique effect on a specific parameter of the predictive distribution. For identical reasons to those described above for tests of interactions, to test such questions, it is necessary to deviate from the default behavior of simultaneously generating all predictive distribution parameters from a single network and instead generate each parameter using a separate subnetwork. Thus, in this case, the location, dispersion, and skewness parameters of the exGaussian distribution are each assigned their own subnetwork, and the critical predictor is ablated from the relevant subnetwork in the null model. For example, to test the unique effect of frequency on the location parameter, the frequency predictor is removed from the subnetwork that generates the location parameter (but not from the subnetworks that generate the dispersion and skewness parameters). As in the case of interactions, the alternative models in these comparisons use a matched architecture (distinct subnetworks for the distinct predictive distribution parameters), except that each subnetwork has access to every predictor in the model.

## A.3 Testing protocol

Following Shain and Schuler (2022), in order to account for optimization noise, statistical tests compare *ensembles* of 10 model replicates per hypothesis, using a hierarchical paired permutation test inspired by Winkler et al. (2014). Tests use the following procedure, in which $A$ is the ensemble size for hypothesis $\mathcal{A}$ and $B$ is the ensemble size for hypothesis $\mathcal{B}$:

1. For each of the $N$ evaluation items $1 \leq n \leq N$, repartition the $A + B$ log-likelihood statistics into two random sets of likelihoods $\hat{\mathcal{A}}_n \in \mathbb{R}^A$, $\hat{\mathcal{B}}_n \in \mathbb{R}^B$.

2. Compute the resampled dataset likelihood as the median of summed likelihoods within the resampled partition:

   $\mathcal{L}_{\hat{\mathcal{A}}} = \underset{1\leq a\leq A}{\text{med}} \left[\sum_{n=1}^{N} \hat{\mathcal{A}}_{n,a}\right], \mathcal{L}_{\hat{\mathcal{B}}} = \underset{1\leq b\leq B}{\text{med}} \left[\sum_{n=1}^{N} \hat{\mathcal{B}}_{n,b}\right]$

3. Compute and store the absolute difference $|\mathcal{L}_{\mathcal{A}} - \mathcal{L}_{\mathcal{B}}|$.

This process is repeated many times to construct an empirical null distribution over the likelihood differences between ensembles, which is then compared to the observed difference in mean likelihood between ensembles in order to compute a $p$ value. Cases where test set likelihood *degrades* are assigned a default $p$ value of 1.

Some tests also combine all response variables from all datasets in order to test comparisons across the entire set. To do so, given $D$ dataset-response pairs (in this study, $D = 11$) with $M$ total datapoints

between them, the item-wise likelihood matrices are vertically concatenated into joint likelihood matrices $\mathcal{A}^{(\text{all})} \in \mathbb{R}^{M \times A}, \mathcal{B}^{(\text{all})} \in \mathbb{R}^{M \times B}$ as follows:

$$\mathcal{A}^{(\text{all})} = \begin{bmatrix} \mathcal{A}^{(1)} \\ \vdots \\ \mathcal{A}^{(M)} \end{bmatrix}, \mathcal{B}^{(\text{all})} = \begin{bmatrix} \mathcal{B}^{(1)} \\ \vdots \\ \mathcal{B}^{(M)} \end{bmatrix}$$

These combined likelihood matrices serve as inputs to the testing procedure outlined above.

# B Visualization protocol

Because the IRF in CDRNN models is implemented as a feedforward neural network, it implicitly constructs a non-linear manifold over the vector space defined by the predictors, the timestamp, and the temporal offset (difference in timestamp between the predictor and the response). This manifold is hard to interpret directly given the difficulty of visualizing functions in more than two variables. Therefore, following Shain and Schuler (2022), visualizations in this study represent an *average case* analysis in which all variables are clamped at their expected values (empirical means in the training data) *except* the independent variable (or variables in the case of surface plots) on the horizontal plot axis, which is systematically manipulated in order to compute the predicted deviation in the response as a result of the manipulation. Uncertainty intervals are computed by resampling the model from the variational posterior under different dropout masks (Gal and Ghahramani, 2016). For additional details on CDRNN visualization and uncertainty estimation, see (Shain and Schuler, 2022).

All visualizations aggregate across the entire ensemble of CDRNN models for a given model configuration, using 1000 bootstrap resampling iterations. In each iteration, an ensemble component (i.e., a CDRNN fit) is sampled uniformly, then a model is sampled from that component's variational posterior, then the sampled model is queried with respect to the estimate of interest. This procedure jointly takes into account uncertainty in the posterior of each CDRNN fit as well as uncertainty across the ensemble.

# C Bivariate impulse response functions

**Figures S1–S5** show three-dimensional surface plots representing the *impulse response function* (IRF) over the empirical interdecile range of values for the frequency and predictability predictors. As shown, processing difficulty increases smoothly on each predictor, with similar IRF shape across the range of predictor values.

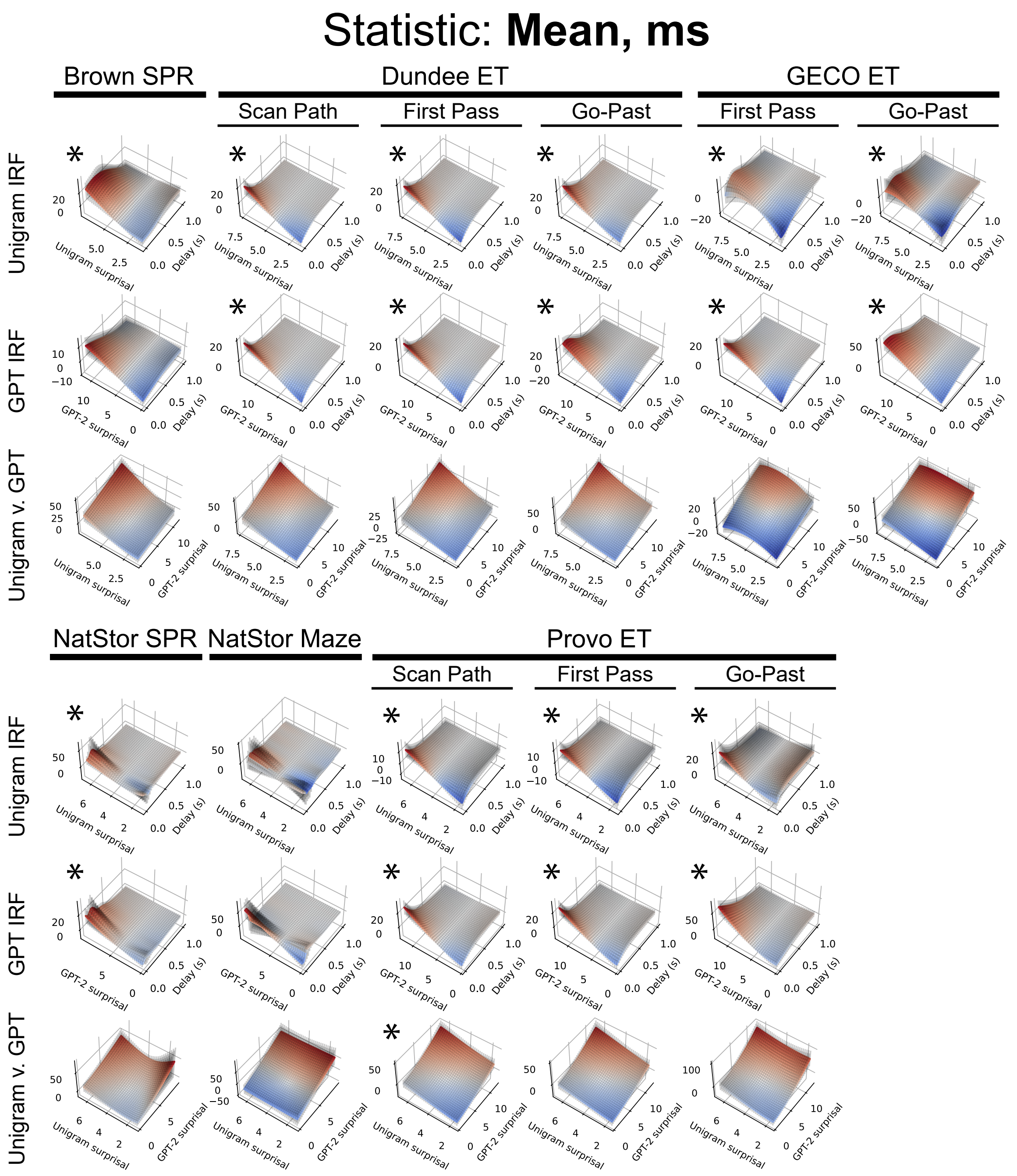


**Figure S1:** Model estimates across datasets for the effect of predictability and frequency on the mean of the exGaussian distribution. Plots show 3D surfaces representing the estimated change in the mean (vertical axis) as a function of two key variables (horizontal axes), with 95% variational credible intervals plotted as gray bars. For rows labeled *Unigram IRF* or *GPT IRF*, the two independent variables are respectively unigram or GPT-2 surprisal (left axis) and delay over a 1s interval following stimulus onset (right axis). These plots thus show *impulse response functions*, that is, the evolution of the statistic over time in response to a word as the reading process unfolds. For rows labeled *Unigram v. GPT* (reproduced from **Figure 1** for convenience), the two independent variables are unigram surprisal (left) and GPT-2 surprisal (right). These plots show the *interaction* between these two variables at a delay of 0s (i.e., at the current word). Non-interactions manifest in these plots as additive surfaces, e.g., a flat plane. Plots showing effects that make a significant unique contribution to generalization likelihood are marked with $*$.

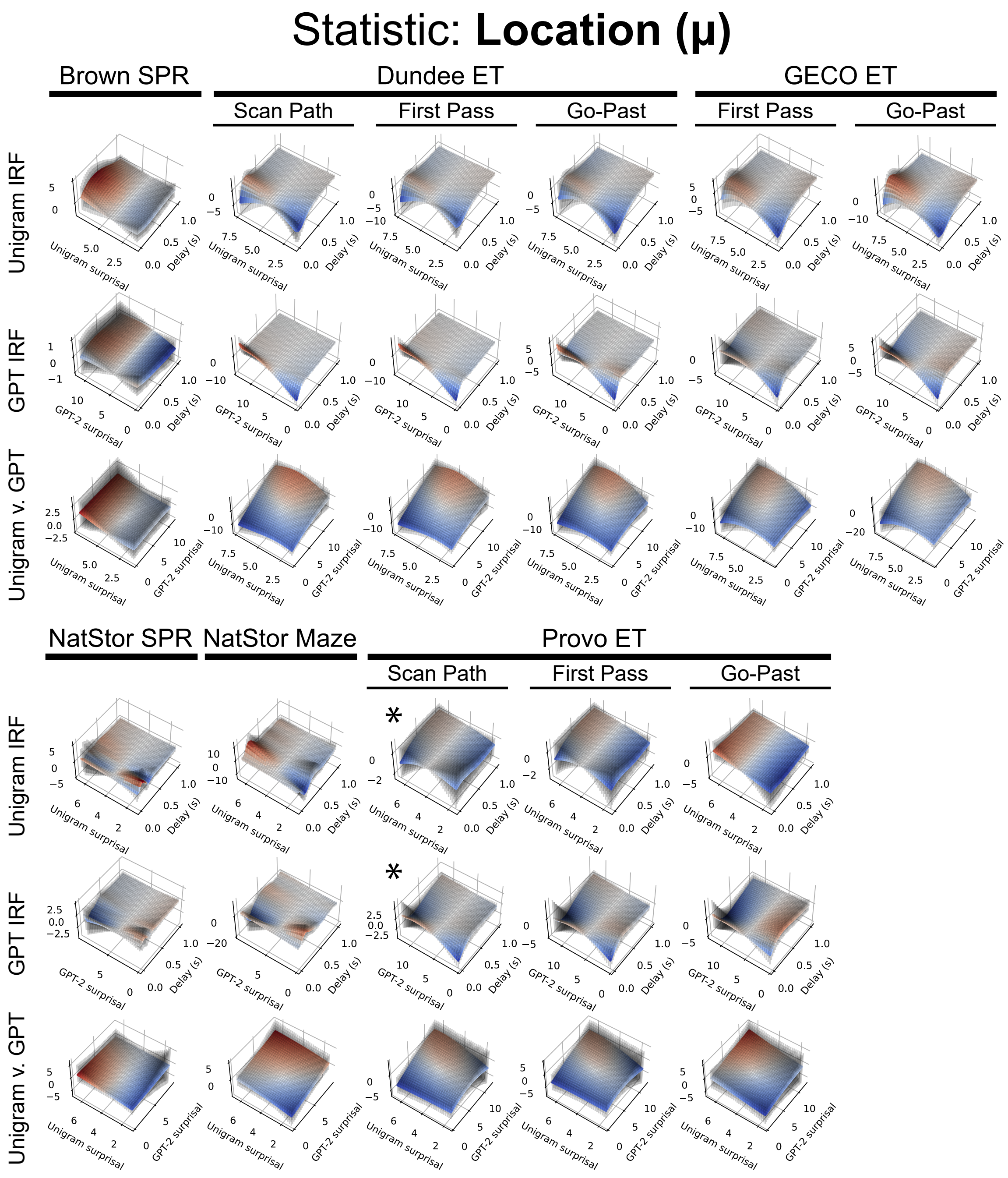


**Figure S2:** Model estimates across datasets for the effect of predictability and frequency on the location parameter ($\mu$) of the exGaussian distribution. Plots show 3D surfaces representing the estimated change in $\mu$ (vertical axis) as a function of two key variables (horizontal axes), with 95% variational credible intervals plotted as gray bars. For rows labeled *Unigram IRF* or *GPT IRF*, the two independent variables are respectively unigram or GPT-2 surprisal (left axis) and delay over a 1s interval following stimulus onset (right axis). These plots thus show *impulse response functions*, that is, the evolution of the statistic over time in response to a word as the reading process unfolds. For rows labeled *Unigram v. GPT* (reproduced from **Figure 1** for convenience), the two independent variables are unigram surprisal (left) and GPT-2 surprisal (right). These plots show the *interaction* between these two variables at a delay of 0s (i.e., at the current word). Non-interactions manifest in these plots as additive surfaces, e.g., a flat plane. Plots showing effects that make a significant unique contribution to generalization likelihood are marked with $*$. Note that, because CDRNNs implicitly rescale the dependent variable for efficient training, the units of the change in $\mu$ are not interpretable in ms (unlike effects on the mean).

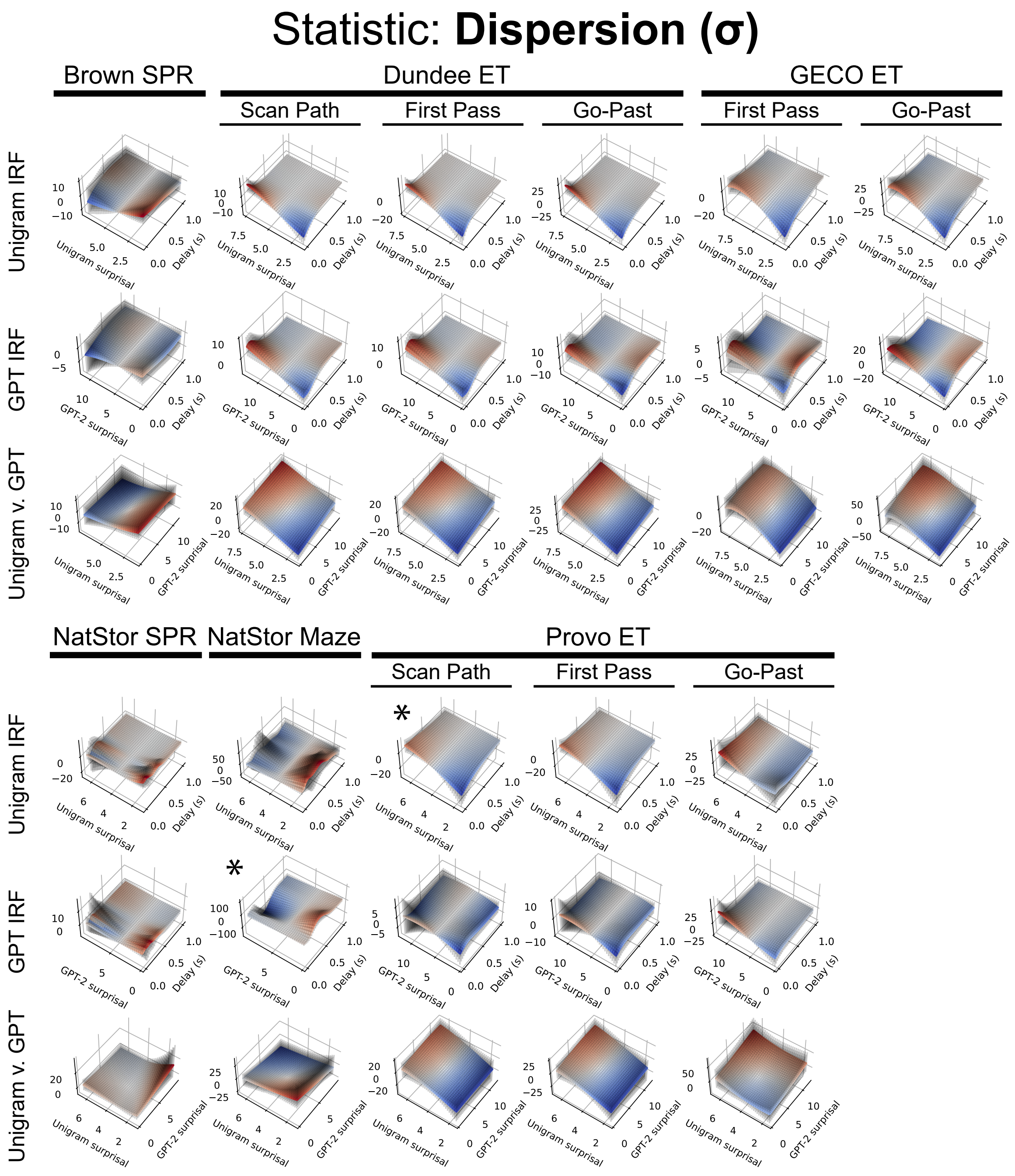


**Figure S3:** Model estimates across datasets for the effect of predictability and frequency on the dispersion parameter ($\sigma$) of the exGaussian distribution. Plots show 3D surfaces representing the estimated change in $\sigma$ (vertical axis) as a function of two key variables (horizontal axes), with 95% variational credible intervals plotted as gray bars. For rows labeled *Unigram IRF* or *GPT IRF*, the two independent variables are respectively unigram or GPT-2 surprisal (left axis) and delay over a 1s interval following stimulus onset (right axis). These plots thus show *impulse response functions*, that is, the evolution of the statistic over time in response to a word as the reading process unfolds. For rows labeled *Unigram v. GPT* (reproduced from **Figure 1** for convenience), the two independent variables are unigram surprisal (left) and GPT-2 surprisal (right). These plots show the *interaction* between these two variables at a delay of 0s (i.e., at the current word). Non-interactions manifest in these plots as additive surfaces, e.g., a flat plane. Plots showing effects that make a significant unique contribution to generalization likelihood are marked with $*$. Note that, because CDRNNs implicitly rescale the dependent variable for efficient training, the units of the change in $\sigma$ are not interpretable in ms (unlike effects on the mean).

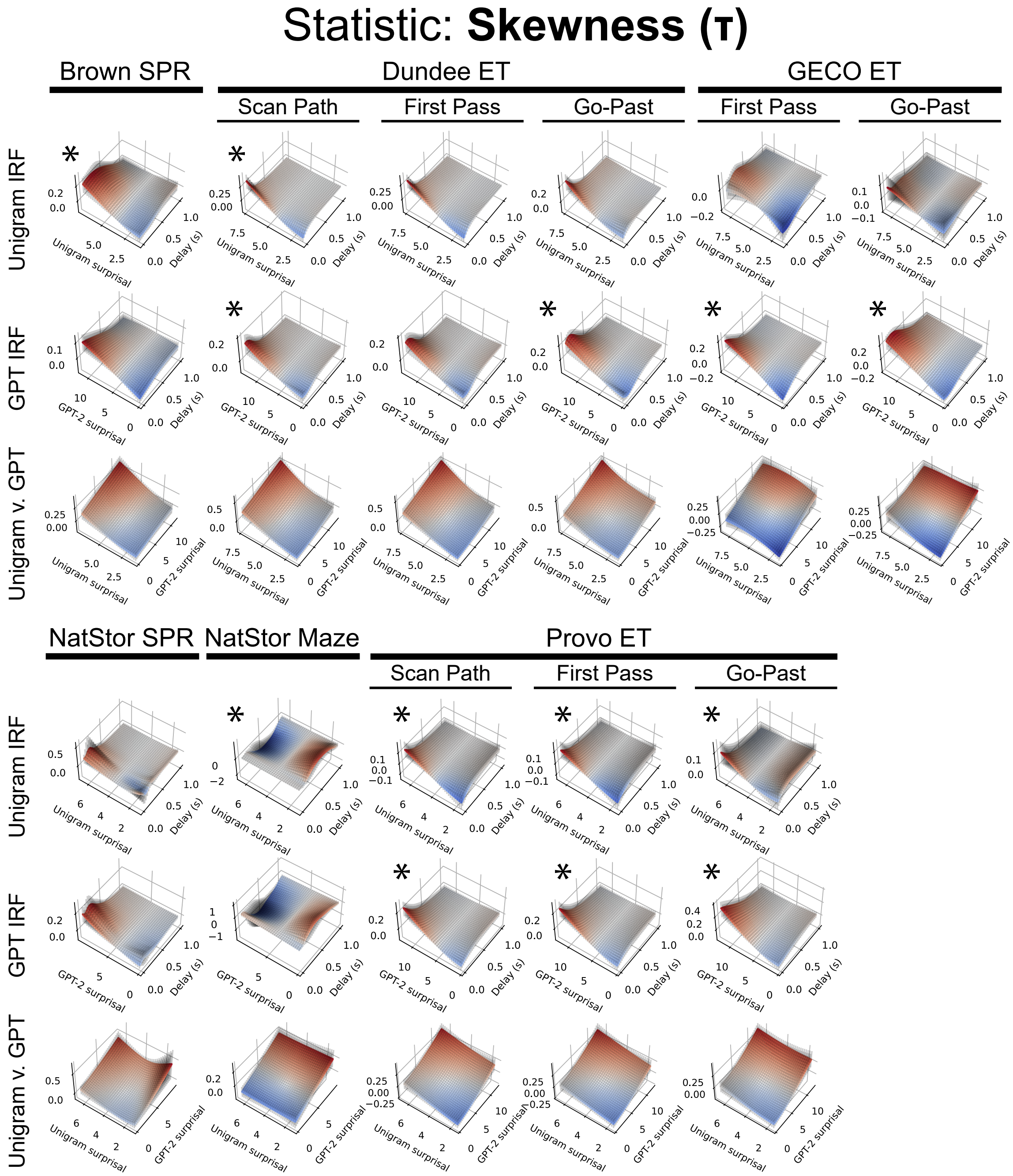


**Figure S4:** Model estimates across datasets for the effect of predictability and frequency on the skewness parameter ($\tau$) of the exGaussian distribution. Plots show 3D surfaces representing the estimated change in $\tau$ (vertical axis) as a function of two key variables (horizontal axes), with 95% variational credible intervals plotted as gray bars. For rows labeled *Unigram IRF* or *GPT IRF*, the two independent variables are respectively unigram or GPT-2 surprisal (left axis) and delay over a 1s interval following stimulus onset (right axis). These plots thus show *impulse response functions*, that is, the evolution of the statistic over time in response to a word as the reading process unfolds. For rows labeled *Unigram v. GPT* (reproduced from **Figure 1** for convenience), the two independent variables are unigram surprisal (left) and GPT-2 surprisal (right). These plots show the *interaction* between these two variables at a delay of 0s (i.e., at the current word). Non-interactions manifest in these plots as additive surfaces, e.g., a flat plane. Plots showing effects that make a significant unique contribution to generalization likelihood are marked with $*$. Note that, because CDRNNs implicitly rescale the dependent variable for efficient training, the units of the change in $\tau$ are not interpretable in ms (unlike effects on the mean).

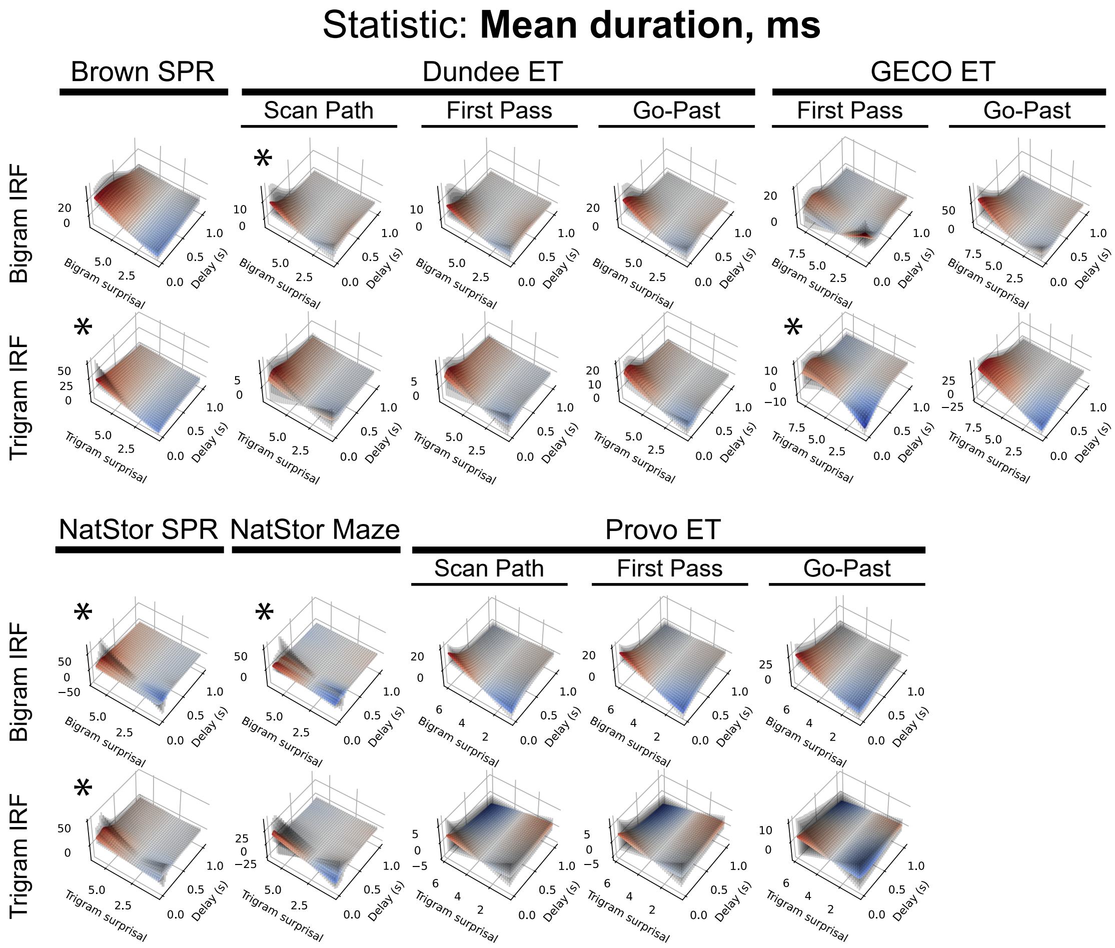


**Figure S5:** Model estimates across datasets of the expected influence of bigram and trigram surprisal on reading times. Plots show 3D surfaces representing the estimated change in the critical parameter (vertical axis) as a function of two key variables (horizonal axes), with 95% variational credible intervals plotted as gray bars. The two independent variables are respectively bigram or trigram surprisal (left axis) and delay over a 1s interval following stimulus onset (right axis). These plots thus show *impulse response functions*, that is, the evolution of reading times in response to a word as the reading process unfolds. Plots showing effects that make a significant unique contribution to generalization likelihood are marked with $*$.

# D Univariate impulse response functions for critical and control predictors

To better understand the role of frequency and predictability in the context of the diverse control predictors included in this study, univariate IRFs are plotted in **Figure S6**. These visualizations reveal potentially important patterns in the effects of control predictors. For example, consistent with prior deconvolutional analyses (Shain and Schuler, 2018, 2021, 2022), the deconvolutional intercept *rate* (which reflects the average-case effect of observing a word, independently of its properties) is generally given large-magnitude and negative estimates, especially in self-paced experiments (Brown, Natural Stories SPR, Natural Stories Maze). This *rate* effect emerges despite the fact that these nonstationary models implicitly capture non-linear changes in response over time, suggesting that *rate* is not merely being commandeered to account for task habituation (e.g., Prasad and Linzen, 2021). Reading therefore appears to have a "bursty" structure whereby faster reading in the recent past engenders faster reading now, especially in self-paced reading, which could potentially reflect attention, motor "inertia" from repeated button pressing, or other effects overlaid onto language processing.

Another effect of potential importance is the *end of sentence*, which is associated with large estimated increases in reading time over an extended delay (hundreds of ms). In other words, reaching the end of a sentence is associated with a slowdown that extends over multiple subsequent fixations (typically, to the initial words of the following sentence). Note that this effect appears despite the fact that starts and ends of sentences are removed from all response variables (standard practice in naturalistic reading modeling), suggesting that these boundary effects are not restricted to words immediately adjacent to sentence boundaries, and thus that filtering boundaries may be insufficient to remove their influence on reading behavior (as discussed in Section 2.1, all words, including sentence boundaries, are retained in the predictor matrix, allowing *end of sentence* effects to be identified despite filtering boundaries from the response).

Eye-tracking datasets show an intriguing dissociation in the *saccade length* effect (i.e., the number of words traversed on the inbound saccade) such that longer inbound saccades tend to be associated with faster scan path and first pass durations but slower go-past durations. One possible reason for this pattern could be that longer saccades may be sub-optimal for comprehension and encourage readers to quickly regress back to preceding words for missing information, resulting in shorter scan path and first pass durations (which exclude regressive eye movements) and longer go-past durations (which include them). Additional work is needed to test this speculation directly.

These comments are not exhaustive (other potentially important patterns may be present in these estimates) and are purely based on visualizations without direct testing. These estimates and speculative interpretations are simply included as potential pointers for future research.

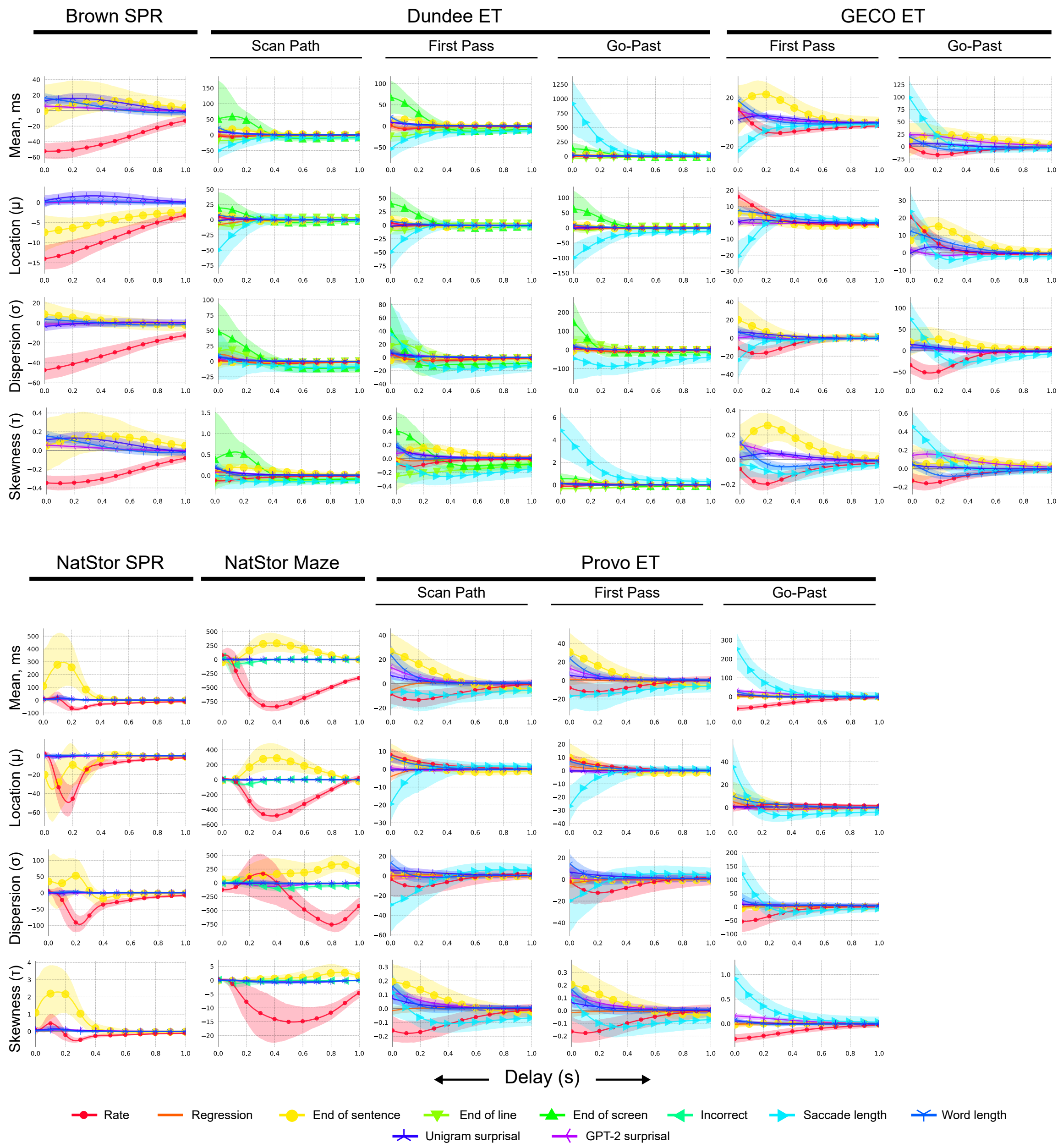


**Figure S6:** Univariate impulse response functions (IRFs) across datasets and response statistics (mean reading time in ms, as well as the location, dispersion, and skewness parameters of the exGaussian distribution) for the critical frequency (*unigram surprisal*) and predictability (*GPT-2 surprisal*) predictors, along with all control predictors. Plots show the estimated change in the response statistic over time as a result of observing a word with one standard deviation over the mean (for continuous predictors like *unigram surprisal*) or a value of one (for indicators like *end of sentence*). The $x$-axis shows delay from initial fixation, where delay 0s is the instantaneous effect (i.e., the effect on the fixated word) and delay ¿ 0s reflects effects on subsequent words as a function of continuous time. Uncertainty bands represent 95% variational credible intervals.

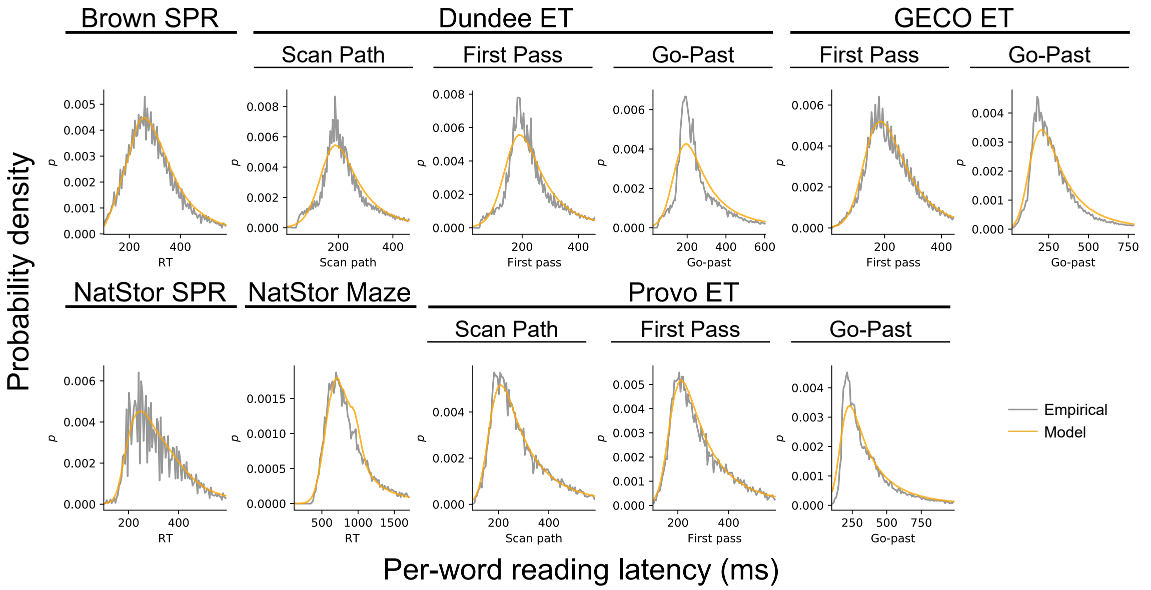


**Figure S7:** Out-of-sample variational posterior predictive checks. Plots overlay histograms of the empirical distribution (in gray) of observed test-set reading latencies (excluding the top 5% tail for legibility) and synthetic reading latencies (in orange) derived from 100 simulated datasets drawn from the main CDRNN model ensemble's variational posterior distribution. Models provide a close fit to observed distributions, even though they were not trained on these data.

# E Overall model fit

The statistical tests in **Tables S2–S6** are based on relative CDRNN model fit to the out-of-sample test set, but do these models provide a good fit to reading data in general, as indicated by prior work (Shain, 2021; Shain and Schuler, 2022)? This section addresses this question qualitatively and quantitatively.

Qualitatively, **Figure S7** presents posterior predictive checks showing histograms of human (empirical) and CDRNN-simulated (model) reading latencies in the out-of-sample test set of each dataset. The empirical histograms represent the raw data (following any preprocessing, see **Section 2.1**), with the top 5% tail of reading latencies excluded for legibility. The model histograms are computed by drawing simulated datasets from the variational posterior defined by an ensemble of 10 CDRNN models in the *Main 1* configuration (**SI L**). To do so, for a given dataset, an ensemble component is first drawn uniformly, then a model parameterization is drawn from the sampled ensemble component (for details about the variational methods used to define an approximate posterior distribution over model parameters, see Shain and Schuler, 2021, 2022). Per-word response distributions are then computed by applying the sampled model to the predictor matrix of the test set, and a per-word simulated response is then drawn from the corresponding distribution, resulting in a dataset simulated from the model's variational posterior. This procedure is repeated 100 times per dataset, resulting in 100 simulated datasets. As shown, CDRNNs provide a close fit to observed reading latencies on unseen data.

Quantitatively, two measures of out-of-sample model fit by dataset are given in **Table S1**: the Pearson correlation between observations and model predictions (defined as the mode of the model-generated exGaussian response distribution) on the test set ($\rho_{\text{pred}}$), and the conditional likelihood assigned by the fitted model to the test set (LL). Each cell in **Table S1** represents the median performance across the corresponding ensemble of 10 CDRNN models. The $\rho_{\text{pred}}$ measure is included because of its intuitive interpretation, but it is a conservative estimate of model fit because the right-skewed exGaussian models used in this study do not necessarily optimize Pearson correlation. The LL measure provides a fuller picture of fit because it directly reflects the probability density assigned by the model to the data, thereby taking into account the gains from modeling the skewed (exGaussian) distribution of responses, as well as the full distributional effects of word features like frequency and predictability. Because these are out-of-sample measures, there is no necessary

| Dataset | Response | Intercept-only model $\rho_{\text{pred}}$ | Intercept-only model LL | Control-only model $\rho_{\text{pred}}$ | Control-only model LL | Full model $\rho_{\text{pred}}$ | Full model LL |
|---|---|---|---|---|---|---|---|
| Brown | RT | — | -184097 | 0.493 | -169179 | **0.496** | **-168819** |
| Dundee | Scan path | — | -380515 | 0.320 | -372797 | **0.347** | **-372105** |
| | First pass | — | -286838 | 0.355 | -280839 | **0.382** | **-280215** |
| | Go-past | — | -300201 | 0.190 | -293129 | **0.210** | **-292445** |
| GECO | First pass | — | -445812 | 0.364 | -435066 | **0.373** | **-434643** |
| | Go-past | — | -486045 | 0.207 | -470449 | **0.212** | **-469863** |
| Natural Stories SPR | RT | — | -1169357 | 0.558 | -1053855 | **0.559** | **-1052551** |
| Natural Stories Maze | RT | — | -101590 | **0.745** | **-73709** | 0.744 | -73781 |
| Provo | Scan path | — | -222803 | 0.212 | -218870 | **0.221** | **-218690** |
| | First pass | — | -155553 | 0.232 | -152486 | **0.240** | **-152353** |
| | Go-past | — | -167867 | 0.200 | -163798 | **0.201** | **-163668** |

**Table S1:** Median out-of-sample model fit by dataset for each of three model types fitted to the training set and evaluated on the test set: *intercept-only* (a single fixed setting for the three parameters of the exGaussian distribution), *control-only* (a model with all control predictors but no frequency or predictability predictors), and *full* (the full model). Two measures of fit are provided: conditional log likelihood (LL) and $\rho_{\text{pred}}$ (Pearson correlation between observed reading latencies and the mode of the model's response distribution; undefined for the intercept-only model, which has no variance). The model with the best performance (highest $\rho_{\text{pred}}$ and LL) is **boldfaced** in each row.

relationship between model complexity and model fit: additional complexity in the model will only improve fit to the extent that the same patterns also hold in an unseen data sample.

**Table S1** evaluates three model types per dataset: an *intercept-only* model that estimates a single fixed set of three exGaussian parameters and otherwise treats reading latencies as independent and identically distributed, a *control-only* model that estimates all fixed and random effects *except* the critical frequency and predictability predictors, and the *full* model that includes all predictors (frequency, predictability, and controls). As shown, the control-only model typically improves on the intercept-only model by thousands of log likelihood points. This means that modeling effects of word features, subjects, and items on reading behavior over time leads to large improvements in generalization to unseen data. Likewise, the full model typically improves on the control-only model by hundreds of log likelihood points (except for Natural Stories Maze, where out-of-sample fit degrades, as reported in **Table S2**). This means that in almost all cases, modeling the critical frequency and predictability features at issue in this study leads to further substantial gains in model fit. Thus, evidence suggests that the CDRNN models used in this study capture generalizable patterns of effects that provide a strong fit to reading data.

# F Model-free data visualizations

To supplement the model-based visualizations used throughout this work, a model-free counterpart to **Figure 1** is provided in **Figure S8**. These visualizations aggregate the raw responses in each dataset (following any preprocessing, see **Section 2.1** of the main article) based on frequency and predictability quartiles, in order to show how responses change overall as a function of frequency and predictability. The large uncertainty intervals are unsurprising in light of known structure in the data that these model-free analyses cannot account for, including subject and item effects, delayed effects, and influences of confounding variables. The ability to control for these sources of heterogeneity is a key advantage of analyzing these datasets using CDRNNs (as in the main article). Nevertheless, visualizations are consistent with the key model-based results in the main article: reading latencies increase for less predictable words (higher GPT-2 surprisal) within each frequency (Unigram surprisal) bin and for less frequent words within each predictability bin, supporting dissociable effects.

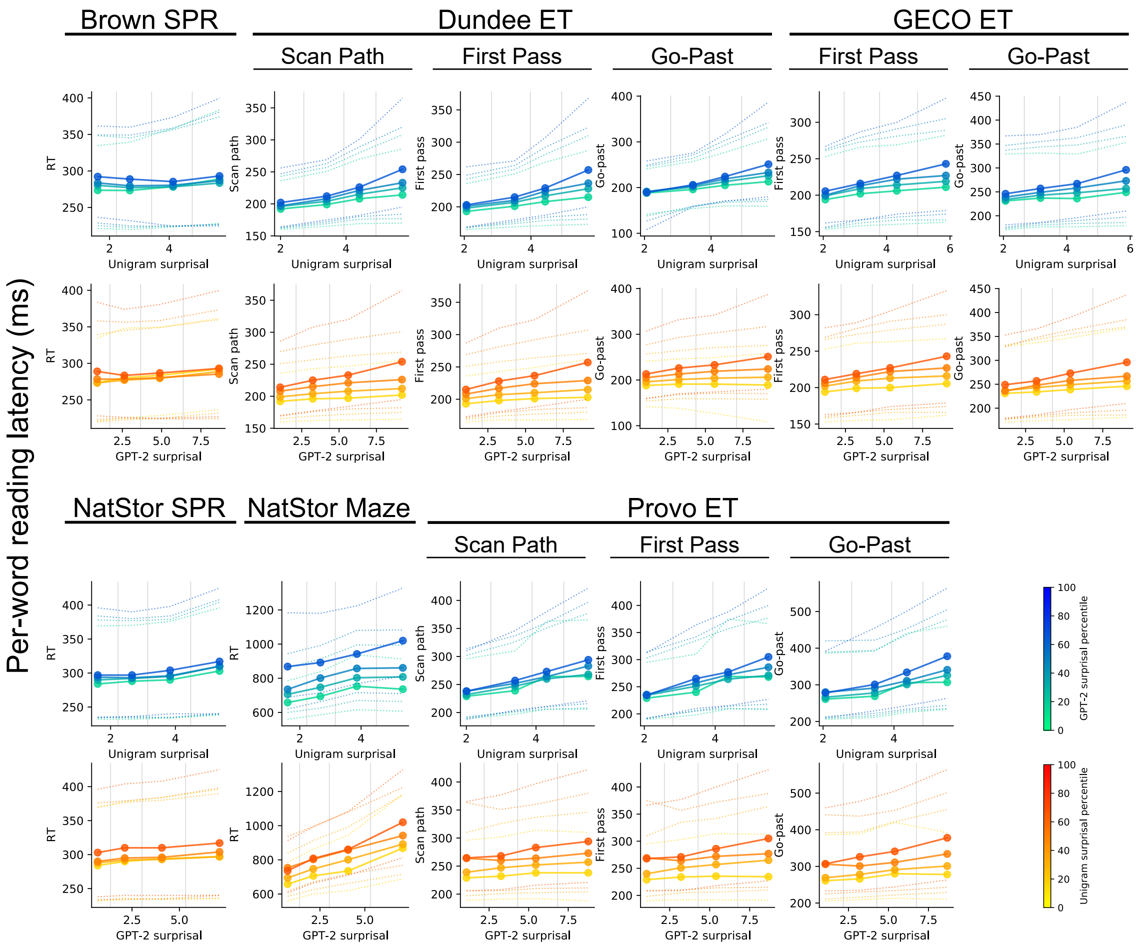


**Figure S8:** Model-free data visualizations. Plots show the median response in each quartile of one predictor ($x$-axis) within each quartile (line colors) of the other predictor. Quartile boundaries are shown as vertical lines. Dotted lines show the interquartile range. Points are placed at the median value within each $x$-axis quartile.

| Comparison | Brown (SPR) ΔLL | Brown (SPR) $p$ | NatStor (SPR) ΔLL | NatStor (SPR) $p$ | NatStor (Maze) ΔLL | NatStor (Maze) $p$ |
|---|---|---|---|---|---|---|
| Frequency | **338** | **0.0006** | **575** | **0.0005** | -8 | — |
| Predictability | **52** | **0.0006** | **923** | **0.0005** | **129** | **0.0004** |
| Frequency over predictability | **309** | **0.0006** | **381** | **0.0005** | -202 | — |
| Predictability over frequency | 23 | 0.2630 | **729** | **0.0005** | -65 | — |
| Frequency-predictability interaction | -116 | — | -573 | — | -437 | — |
| Bigram predictability | -99 | — | **258** | **0.0005** | **116** | **0.0004** |
| Frequency over bigram predictability | 28 | 0.0624 | **885** | **0.0005** | **287** | **0.0004** |
| GPT-2 predictability over bigram predictability | -68 | — | **364** | **0.0005** | 32 | 0.4322 |
| Trigram predictability | **30** | **0.0408** | **350** | **0.0005** | -486 | — |
| Frequency effect on $\mu$ | 19 | 0.2630 | 457 | 1.0000 | -281 | — |
| Frequency effect on $\sigma$ | -17 | — | -1018 | — | -385 | — |
| Frequency effect on $\tau$ | **90** | **0.0006** | -852 | — | **529** | **0.0004** |
| Predictability effect on $\mu$ | -47 | — | -138 | — | -567 | — |
| Predictability effect on $\sigma$ | -36 | — | -1612 | — | **567** | **0.0004** |
| Predictability effect on $\tau$ | 15 | 0.5740 | -317 | — | 73 | 0.5357 |

**Table S2:** Testing results on self-paced datasets. Results of key statistical comparisons based on permutation tests of the difference in median test set likelihood between the alternative and null model in each comparison (ΔLL). **Boldface** indicates statistical significance. Dashes (—) indicate failure to improve over a baseline. $p$-values are corrected for false discovery rate (Benjamini and Yekutieli, 2001) across all comparisons per response variable.

# G Full significance testing results

Tables S2–S6 provide the full results of all statistical tests conducted in this study.

| Comparison | Scan Path ΔLL | Scan Path $p$ | First Pass ΔLL | First Pass $p$ | Go Past ΔLL | Go Past $p$ |
|---|---|---|---|---|---|---|
| Frequency | **582** | **0.0007** | **504** | **0.0007** | **496** | **0.0007** |
| Predictability | **521** | **0.0007** | **467** | **0.0007** | **554** | **0.0007** |
| Frequency over predictability | **170** | **0.0007** | **157** | **0.0007** | **129** | **0.0007** |
| Predictability over frequency | **109** | **0.0007** | **120** | **0.0007** | **187** | **0.0007** |
| Frequency-predictability interaction | -45 | — | -24 | — | -21 | — |
| Bigram predictability | **30** | **0.0075** | 9 | 1.0000 | 7 | 1.0000 |
| Frequency over bigram predictability | **96** | **0.0007** | **78** | **0.0007** | **85** | **0.0007** |
| GPT-2 predictability over bigram predictability | **100** | **0.0007** | **74** | **0.0007** | **88** | **0.0007** |
| Trigram predictability | -15 | — | 1 | 1.0000 | -14 | — |
| Frequency effect on $\mu$ | -25 | — | -40 | — | -21 | — |
| Frequency effect on $\sigma$ | -18 | — | -11 | — | -41 | — |
| Frequency effect on $\tau$ | **27** | **0.0167** | -4 | — | 7 | 1.0000 |
| Predictability effect on $\mu$ | 18 | 0.0879 | 7 | 1.0000 | 16 | 0.5651 |
| Predictability effect on $\sigma$ | -6 | — | -9 | — | -9 | — |
| Predictability effect on $\tau$ | **26** | **0.0159** | 18 | 0.1040 | **125** | **0.0007** |

**Table S3:** Testing results on Dundee (eye-tracking). Results of key statistical comparisons based on permutation tests of the difference in median test set likelihood between the alternative and null model in each comparison (ΔLL). **Boldface** indicates statistical significance. Dashes (—) indicate failure to improve over a baseline. $p$-values are corrected for false discovery rate (Benjamini and Yekutieli, 2001) across all comparisons per response variable.

| Comparison | First Pass ΔLL | First Pass $p$ | Go Past ΔLL | Go Past $p$ |
|---|---|---|---|---|
| Frequency | **227** | **0.0005** | **248** | **0.0008** |
| Predictability | **325** | **0.0005** | **492** | **0.0008** |
| Frequency over predictability | **98** | **0.0005** | **94** | **0.0008** |
| Predictability over frequency | **196** | **0.0005** | **338** | **0.0008** |
| Frequency-predictability interaction | 2 | 1.0000 | -11 | — |
| Bigram predictability | 0 | 1.0000 | -12 | — |
| Frequency over bigram predictability | **73** | **0.0005** | **44** | **0.0012** |
| GPT-2 predictability over bigram predictability | **171** | **0.0005** | **249** | **0.0008** |
| Trigram predictability | **37** | **0.0005** | 8 | 1.0000 |
| Frequency effect on $\mu$ | 4 | 1.0000 | 11 | 1.0000 |
| Frequency effect on $\sigma$ | -34 | — | -30 | — |
| Frequency effect on $\tau$ | 5 | 1.0000 | 9 | 1.0000 |
| Predictability effect on $\mu$ | -4 | — | 3 | 1.0000 |
| Predictability effect on $\sigma$ | -59 | — | -38 | — |
| Predictability effect on $\tau$ | **71** | **0.0005** | **188** | **0.0008** |

**Table S4:** Testing results on GECO (eye-tracking). Results of key statistical comparisons based on permutation tests of the difference in median test set likelihood between the alternative and null model in each comparison (ΔLL). **Boldface** indicates statistical significance. Dashes (—) indicate failure to improve over a baseline. $p$-values are corrected for false discovery rate (Benjamini and Yekutieli, 2001) across all comparisons per response variable.

| Comparison | Scan Path | | First Pass | | Go Past | |
|---|---|---|---|---|---|---|
| | ΔLL | $p$ | ΔLL | $p$ | ΔLL | $p$ |
| Frequency | **120** | **0.0007** | **96** | **0.0010** | **81** | **0.0008** |
| Predictability | **123** | **0.0007** | **96** | **0.0010** | **101** | **0.0008** |
| Frequency over predictability | **57** | **0.0007** | **37** | **0.0010** | **29** | **0.0008** |
| Predictability over frequency | **61** | **0.0007** | **37** | **0.0010** | **49** | **0.0008** |
| Frequency-predictability interaction | **24** | **0.0025** | 12 | 0.2177 | -55 | — |
| Bigram predictability | -31 | — | -9 | — | 0 | 1.0000 |
| Frequency over bigram predictability | -1 | — | -2 | — | -2 | — |
| GPT-2 predictability over bigram predictability | 1 | 1.0000 | -9 | — | 19 | 0.2868 |
| Trigram predictability | 4 | 1.0000 | 8 | 0.6565 | -6 | — |
| Frequency effect on $\mu$ | **18** | **0.0033** | 10 | 0.1763 | -12 | — |
| Frequency effect on $\sigma$ | **16** | **0.0262** | 4 | 1.0000 | -13 | — |
| Frequency effect on $\tau$ | **43** | **0.0007** | **29** | **0.0010** | **43** | **0.0008** |
| Predictability effect on $\mu$ | **15** | **0.0239** | 1 | 1.0000 | 4 | 1.0000 |
| Predictability effect on $\sigma$ | 4 | 1.0000 | -2 | — | -4 | — |
| Predictability effect on $\tau$ | **29** | **0.0007** | **21** | **0.0017** | **27** | **0.0199** |

**Table S5:** Testing results on Provo (eye-tracking). Results of key statistical comparisons based on permutation tests of the difference in median test set likelihood between the alternative and null model in each comparison (ΔLL). **Boldface** indicates statistical significance. Dashes (—) indicate failure to improve over a baseline. $p$-values are corrected for false discovery rate (Benjamini and Yekutieli, 2001) across all comparisons per response variable.

| Comparison | Combined Datasets | |
|---|---|---|
| | ΔLL | $p$ |
| Frequency | **3370** | **0.0005** |
| Predictability | **3879** | **0.0005** |
| Frequency over predictability | **1321** | **0.0005** |
| Predictability over frequency | **1830** | **0.0005** |
| Frequency-predictability interaction | -1257 | — |
| Bigram predictability | **521** | **0.0005** |
| Frequency over bigram predictability | **1928** | **0.0005** |
| GPT-2 predictability over bigram predictability | **1046** | **0.0005** |
| Trigram predictability | -713 | — |
| Frequency effect on $\mu$ | 59 | 1.0000 |
| Frequency effect on $\sigma$ | -1417 | — |
| Frequency effect on $\tau$ | 546 | 0.4165 |
| Predictability effect on $\mu$ | -758 | — |
| Predictability effect on $\sigma$ | -1092 | — |
| Predictability effect on $\tau$ | 364 | 1.0000 |

**Table S6:** Testing results across all datasets. Results of key statistical comparisons based on permutation tests of the difference in median test set likelihood between the alternative and null model in each comparison (ΔLL). **Boldface** indicates statistical significance. Dashes (—) indicate failure to improve over a baseline. $p$-values are corrected for false discovery rate (Benjamini and Yekutieli, 2001) across all comparisons.

# H Effects of frequency and predictability on the parameters of the exGaussian

**Figure 1** of the main article focuses on frequency and predictability effects on mean reading time. Here, similar visualizations are presented for each of the three parameters of the exGaussian distribution assumed throughout this study: location ($\mu$, **Figure S10**), dispersion ($\sigma$, **Figure S11**), and skewness ($\tau$, **Figure S12**), with **Figure 1** reproduced as **Figure S9** for convenient reference. Three key patterns are apparent in these results. *First*, as was the case for the mean response (**Figure S9**), there is little evidence of a frequency-predictability interaction in any of the distributional parameters $\mu$, $\sigma$, or $\tau$: for all three parameters, frequency effects are similar across the predictability continuum and *vice versa*. *Second*, frequency and predictability primarily modulate the response via the $\tau$ parameter: (1) effects on $\tau$ (**Figure S12**) are similar in shape to effects on the mean (**Figure S9**); (2) effects on $\mu$ and $\sigma$ are weak and uncertain relative to effects on $\tau$, and (3) effects on $\tau$ are significant with greater frequency than effects on either $\mu$ or $\sigma$. *Third*, effects of frequency and predictability are similar for a given dataset and distributional parameter, suggesting little difference between frequency and predictability in how they modulate the different parameters of the response distribution. These visual impressions are supported by statistical tests (**Tables S2–S6**), and their theoretical implications are elaborated in the **Discussion** section of the main article.

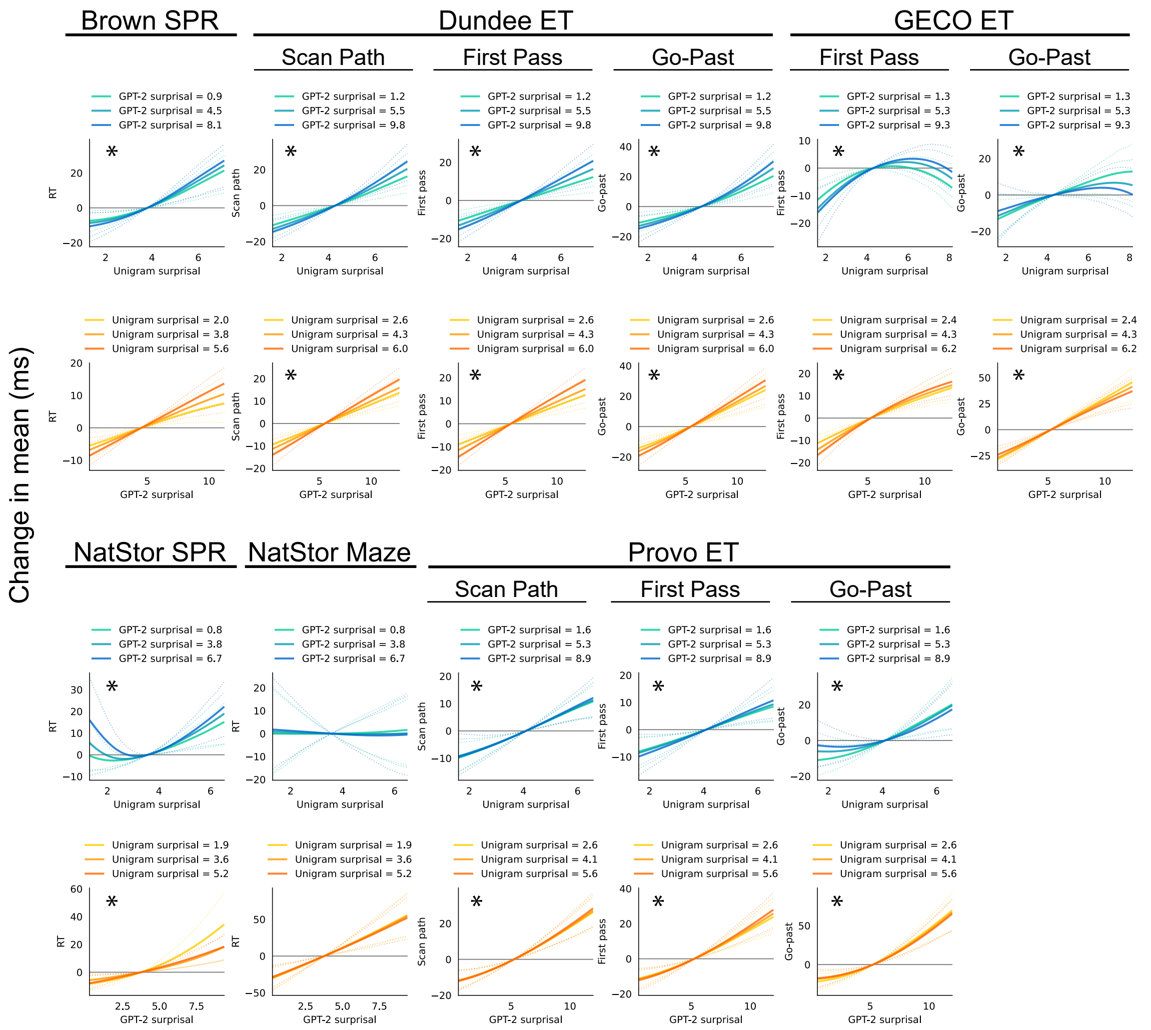


**Figure S9: (Figure 1, reproduced for convenient comparison)** Estimated effects of frequency (Unigram surprisal) and predictability (GPT-2 surprisal) on mean reading time (in ms) across datasets. Plots show how the instantaneous response (i.e., at no delay) deviates from its mean as a function of one predictor ($x$-axis) at three different values (line colors) of the other predictor (mean, $\pm 1$ standard deviation), thus revealing how the frequency effect changes as a function of predictability (and vice versa). Dotted lines show 95% variational Bayesian credible intervals. Plots showing effects that make a significant unique contribution to generalization likelihood are marked with $*$. In all datasets, effects of frequency are similar across the range of predictability values and vice versa, supporting a lack of interaction.

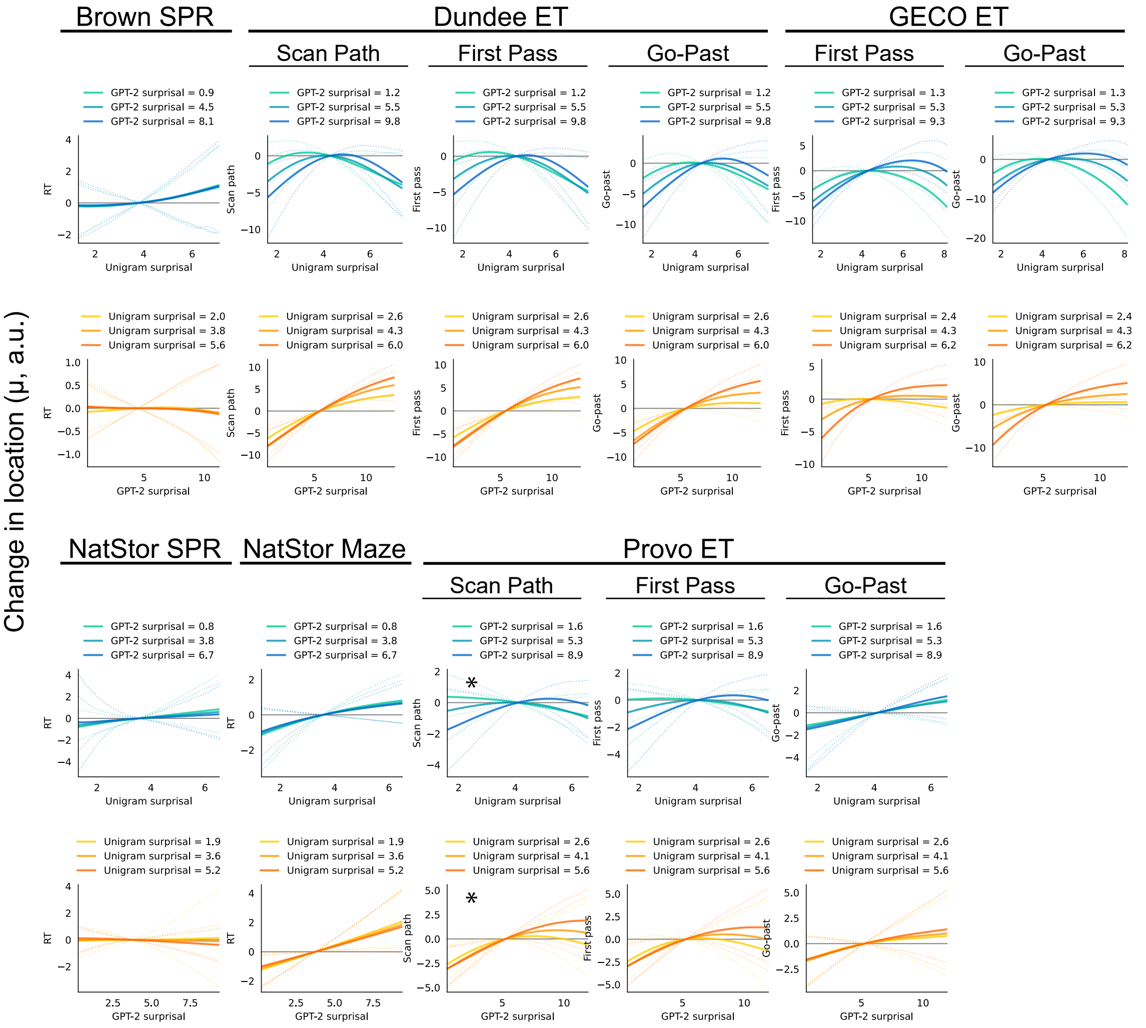


**Figure S10:** Estimated effects of frequency (Unigram surprisal) and predictability (GPT-2 surprisal) on the exGaussian location parameter $\mu$ across datasets. Plots show how the instantaneous response (i.e., at no delay) deviates from its mean as a function of one predictor ($x$-axis) at three different values (line colors) of the other predictor (mean, $\pm 1$ standard deviation), thus revealing how the frequency effect changes as a function of predictability (and *vice versa*). Dotted lines show 95% variational Bayesian credible intervals. Plots showing effects that make a significant unique contribution to generalization likelihood are marked with $*$. Because CDRNNs implicitly rescale the dependent variable for efficient training, the units of $\mu$ are not straightforwardly interpretable (unlike effects on the mean) and are therefore notated as *a.u.* (arbitrary unit). In all datasets, effects of frequency are similar across the range of predictability values and *vice versa*, supporting a lack of interaction.

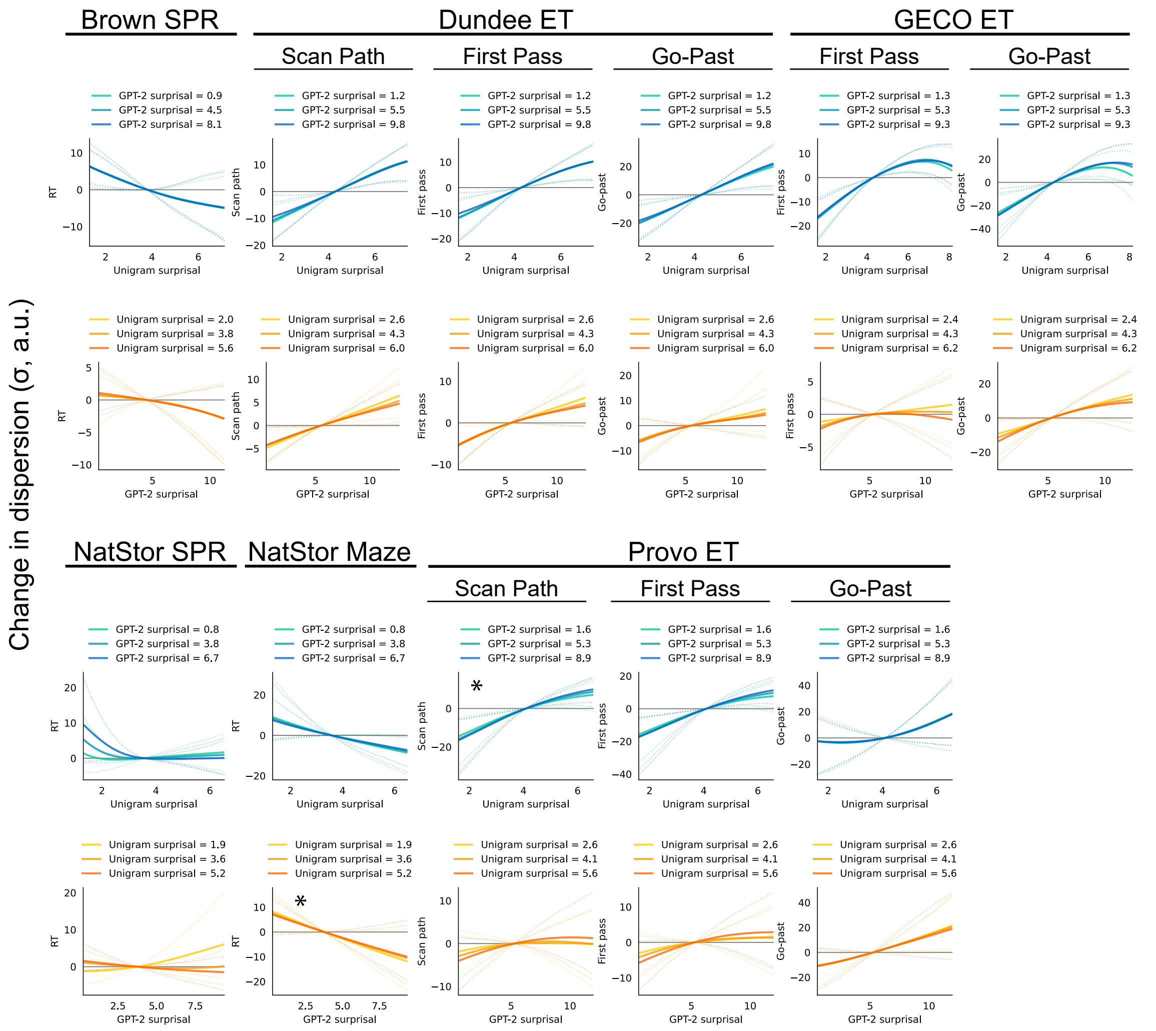


**Figure S11:** Estimated effects of frequency (Unigram surprisal) and predictability (GPT-2 surprisal) on the exGaussian dispersion parameter $\sigma$ across datasets. Plots show how the instantaneous response (i.e., at no delay) deviates from its mean as a function of one predictor ($x$-axis) at three different values (line colors) of the other predictor (mean, $\pm 1$ standard deviation), thus revealing how the frequency effect changes as a function of predictability (and *vice versa*). Dotted lines show 95% variational Bayesian credible intervals. Plots showing effects that make a significant unique contribution to generalization likelihood are marked with $*$. Because CDRNNs implicitly rescale the dependent variable for efficient training, the units of $\sigma$ are not straightforwardly interpretable (unlike effects on the mean) and are therefore notated as *a.u.* (arbitrary unit). In all datasets, effects of frequency are similar across the range of predictability values and *vice versa*, supporting a lack of interaction.

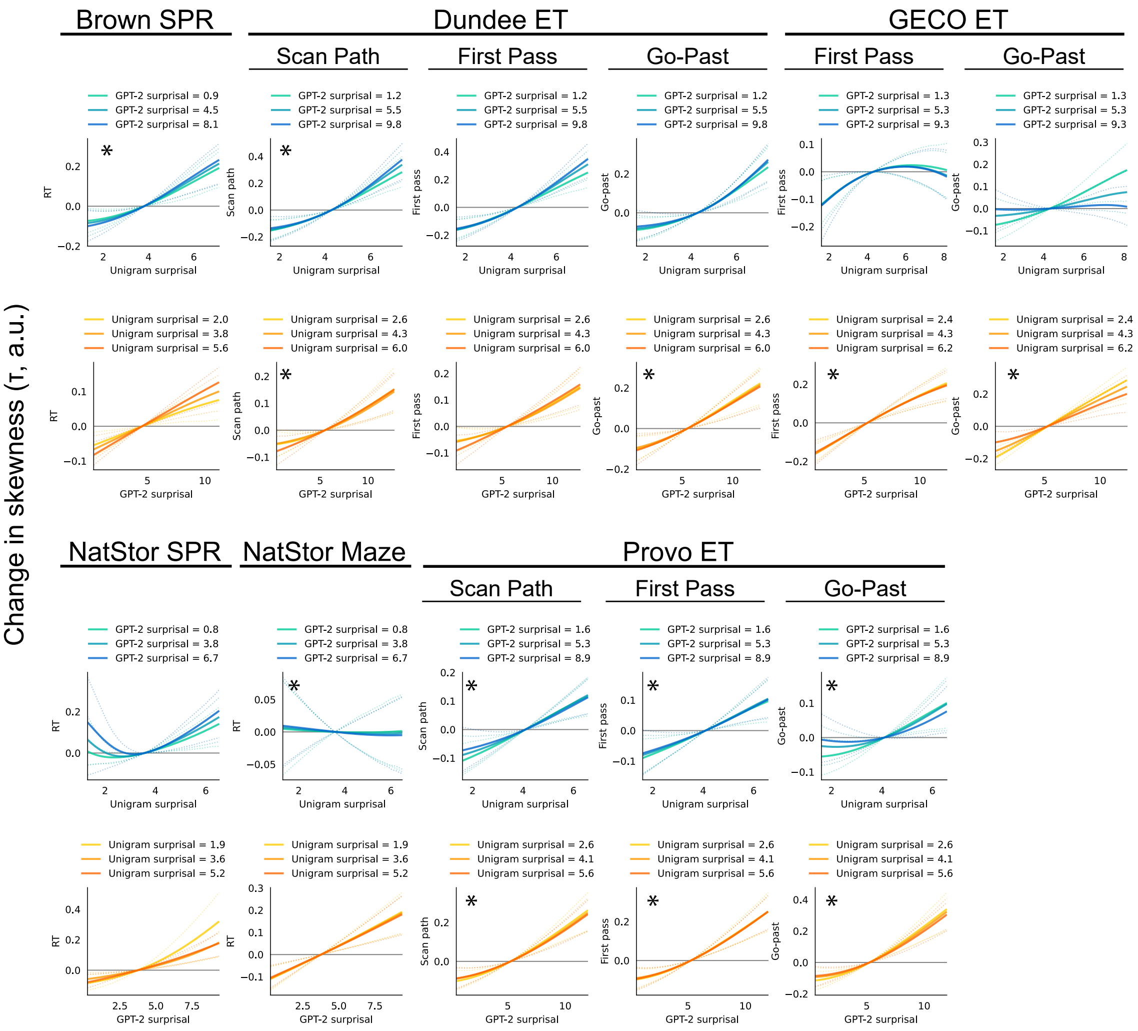


**Figure S12:** Estimated effects of frequency (Unigram surprisal) and predictability (GPT-2 surprisal) on the exGaussian skewness parameter $\tau$ across datasets. Plots show how the instantaneous response (i.e., at no delay) deviates from its mean as a function of one predictor ($x$-axis) at three different values (line colors) of the other predictor (mean, $\pm 1$ standard deviation), thus revealing how the frequency effect changes as a function of predictability (and *vice versa*). Dotted lines show 95% variational Bayesian credible intervals. Plots showing effects that make a significant unique contribution to generalization likelihood are marked with $*$. Because CDRNNs implicitly rescale the dependent variable for efficient training, the units of the $\tau$ are not straightforwardly interpretable (unlike effects on the mean) and are therefore notated as *a.u.* (arbitrary unit). In all datasets, effects of frequency are similar across the range of predictability values and *vice versa*, supporting a lack of interaction.

# I Detailed comparison to Shain (2019)

The results of the present study are in tension with those reported in Shain (2019), where similar methods were used to analyze a subset of the present dataset: the Natural Stories SPR and Dundee datasets, along with the UCL eye-tracking dataset (Frank et al., 2013), which is not analyzed here because short isolated sentences were used and no interstimulus intervals are provided, making IRF identification difficult (see discussion in Shain and Schuler, 2021). Unlike the present study, Shain (2019) did not find evidence of predictability-independent frequency effects: although frequency effects were given small positive estimates by the models, these estimates did not lead to significantly improved generalization to unseen data. Thus, a key prediction of the procedural view was not borne out statistically. Shain (2019) is therefore a salient exception in an empirical landscape otherwise full of evidence of dissociable frequency and predictability effects, both in controlled studies (Altarriba et al., 1996; Lavigne et al., 2000; Rayner et al., 2001, 2004; Ashby et al., 2005; Miellet et al., 2007; Hand et al., 2010; Gollan et al., 2011; Bélanger and Rayner, 2013; Kretzschmar et al., 2015) and in naturalistic reading (Goodkind and Bicknell, 2021, this work). The need to clarify this discrepancy helped motivate the current study.

As acknowledged in Shain (2019), there are limitations to the inferences that are licensed by the findings in Shain (2019). An absence of independent frequency effect is consistent with the inferential view, which predicts such an absence. However, this interpretation makes much of a null result, and absence of evidence is not necessarily evidence of absence, especially under stringent statistical evaluations on held-out data, as Shain (2019) used. Furthermore, the Shain (2019) results are not wholly consistent with the predictions of the inferential view, since Shain (2019) also failed to find a frequency-independent predictability effect in the Dundee corpus, contrary to the hypothesis that predictability is the fundamental driver of frequency effects. Thus, although Shain (2019) found strong evidence that *either* frequency or predictability modulated reading times in Dundee, the results were ambiguous as to which of these was primary.

There are many differences between in Shain (2019) and the present study that could plausibly give rise to divergent results. The present study considers both more datasets (the Brown, GECO, Natural Stories Maze, and Provo datasets were not analyzed in Shain, 2019) and more response variables per dataset (only go-past durations were considered for the Dundee dataset in Shain, 2019). The present study leverages the substantial increases in modeling flexibility afforded by CDRNNs (Shain and Schuler, 2022) over the kernel-based CDR models (Shain and Schuler, 2021) used by Shain (2019): Shain (2019) assumed a narrow family of IRF shapes (the probability density function of a shifted gamma distribution) and an additive, stationary, and homoscedastic reading process (despite the cognitive implausibility of these assumptions, see Shain and Schuler, 2022), not out of principle but simply because the analytical tools needed to relax these assumptions in a continuous-time framework did not yet exist. The present study uses more control predictors (e.g., the *end of sentence* predictor gets large-magnitude estimates, as shown in **Figure S6**, and was not used in Shain, 2019). The present study models raw reading times as the dependent variable, whereas Shain (2019) log-transformed reading times; although the log transform better matches the normality assumption of the Shain (2019) regression models, it indirectly enforces a linear relationship between frequency/predictability and reading times (by modeling log reading times using logarithmic surprisal scale predictors), despite evidence for logarithmic effects of both frequency (e.g., Norris, 2006) and predictability (e.g., Shain et al., to appear) on reading times. And the present study uses an ensembling approach (10 replicates per hypothesis) to address non-convexity and stochastic optimization as potential sources of variation in model performance, whereas the comparisons in Shain (2019) were between pairs of individual models. These considerations recommend the results of the present study as more trustworthy than those of Shain (2019) on first principles.

Nonetheless, in an effort to clarify why the present study reaches different conclusions than Shain (2019) did, this section explores the influence of modeling assumptions on key findings in the Natural Stories SPR and Dundee go-past reading times, which were also analyzed in Shain (2019). Analyses target three classes of difference between the present study and Shain (2019) that could plausibly drive differences in results. *First*, analyses vary whether reading times are raw (present study) or log-transformed (Shain, 2019). *Second*, analyses vary whether predictors are allowed to interact arbitrarily (present study) or are constrained to be linear and additive (Shain, 2019). Linearity and additivity are enforced by removing the predictors from the inputs

| Log | Homoscedastic | Additive | Comparison | Dundee go-past ΔLL | Dundee go-past $p$ | Natural Stories SPR ΔLL | Natural Stories SPR $p$ |
|---|---|---|---|---|---|---|---|
| | | | Predictability over frequency | **187** | **0.0002** | **729** | **0.0002** |
| | | | Frequency over predictability | **129** | **0.0002** | **381** | **0.0002** |
| | | | Frequency | **496** | **0.0002** | **575** | **0.0002** |
| | | | Predictability | **554** | **0.0002** | **923** | **0.0002** |
| | | ✓ | Predictability over frequency | **161** | **0.0002** | **273** | **0.0029** |
| | | | Frequency over predictability | **58** | **0.0002** | **1104** | **0.0004** |
| | | | Frequency | **342** | **0.0002** | -236 | — |
| | | | Predictability | **445** | **0.0002** | -1067 | — |
| | ✓ | | Predictability over frequency | **150** | **0.0342** | **623** | **0.0004** |
| | | | Frequency over predictability | 93 | 0.1775 | **133** | **0.0004** |
| | | | Frequency | **175** | **0.0004** | -163 | — |
| | | | Predictability | **232** | **0.0004** | **328** | **0.0004** |
| | ✓ | ✓ | Predictability over frequency | 1383 | 0.2605 | **1520** | **0.0002** |
| | | | Frequency over predictability | -974 | — | **218** | **0.0002** |
| | | | Frequency | -558 | — | -1552 | — |
| | | | Predictability | **1799** | **0.0200** | -250 | — |
| ✓ | | | Predictability over frequency | **199** | **0.0002** | -313 | — |
| | | | Frequency over predictability | **162** | **0.0002** | **430** | **0.0002** |
| | | | Frequency | **366** | **0.0002** | **1543** | **0.0002** |
| | | | Predictability | **403** | **0.0002** | **800** | **0.0002** |
| ✓ | | ✓ | Predictability over frequency | **169** | **0.0002** | **760** | **0.0003** |
| | | | Frequency over predictability | **49** | **0.0002** | **1098** | **0.0003** |
| | | | Frequency | **273** | **0.0002** | 7 | 1.0000 |
| | | | Predictability | **393** | **0.0002** | -331 | — |
| ✓ | ✓ | | Predictability over frequency | **189** | **0.0002** | **507** | **0.0002** |
| | | | Frequency over predictability | **120** | **0.0002** | **141** | **0.0002** |
| | | | Frequency | **373** | **0.0002** | **747** | **0.0002** |
| | | | Predictability | **442** | **0.0002** | **1113** | **0.0002** |
| ✓ | ✓ | ✓ | Predictability over frequency | 65 | 1.0000 | -9 | — |
| | | | Frequency over predictability | -1 | — | -501 | — |
| | | | Frequency | 43 | 1.0000 | -64 | — |
| | | | Predictability | 109 | 1.0000 | **428** | **0.0004** |

**Table S7: Effect of model design on the frequency-predictability dissociation.** The first three columns index manipulations that move the design away from that used in the present study (which found a frequency-predictability dissociation) towards that of Shain (2019), which did not: *Log* (whether reading times were log-transformed), *Homoscedastic* (whether models assumed a homoscedastic normal predictive distribution), and *Additive* (whether models enforced linear, additive effects of each predictor). For each model type, the table presents the outcomes of the four key statistical tests of dissociation (frequency and predictability both alone and over and above one another). The top-most model is the main one used in the present study and re-presented here for convenience. The bottom-most model is the most unlike the present study and the most like the Shain (2019) study (albeit still with potentially important differences, see **SI I**). $p$-values are corrected for false discovery rate (Benjamini and Yekutieli, 2001) within each family of four comparisons. The main finding is that models based on the (less cognitively plausible) assumptions of Shain (2019) also show lower sensitivity (i.e., they yield fewer significant generalization improvements from adding the critical frequency and predictability variables).

| Comparison | Brown (SPR) | | NatStor (SPR) | | NatStor (Maze) | |
|---|---|---|---|---|---|---|
| | $\Delta$LL | $p$ | $\Delta$LL | $p$ | $\Delta$LL | $p$ |
| Frequency | **107** | **0.0005** | **474** | **0.0004** | **399** | **0.0003** |
| Predictability | **24** | **0.0138** | **832** | **0.0004** | **393** | **0.0003** |
| Frequency over predictability | **88** | **0.0005** | **157** | **0.0004** | -120 | — |
| Predictability over frequency | 5 | 1.0000 | **515** | **0.0004** | -126 | — |
| Frequency-predictability interaction | -32 | — | -1082 | — | -221 | — |
| Bigram predictability | **57** | **0.0005** | -363 | — | **272** | **0.0003** |
| Frequency over bigram predictability | **80** | **0.0005** | -122 | — | **335** | **0.0003** |
| GPT-2 predictability over bigram predictability | **64** | **0.0005** | -370 | — | -231 | — |
| Trigram predictability | -160 | — | **492** | **0.0004** | -274 | — |
| Frequency effect on $\mu$ | **35** | **0.0009** | -348 | — | **212** | **0.0003** |
| Frequency effect on $\sigma$ | **50** | **0.0005** | -156 | — | **444** | **0.0003** |
| Frequency effect on $\beta$ | **182** | **0.0005** | -28 | — | **867** | **0.0003** |
| Predictability effect on $\mu$ | **40** | **0.0005** | **872** | **0.0004** | **1317** | **0.0003** |
| Predictability effect on $\sigma$ | -6 | — | -298 | — | **1779** | **0.0003** |
| Predictability effect on $\beta$ | **86** | **0.0005** | -48 | — | **1379** | **0.0003** |

**Table S8:** Testing results on self-paced datasets using a 95% cutoff (parallels **Table S2**). Results of key statistical comparisons based on permutation tests of the difference in median test set likelihood between the alternative and null model in each comparison ($\Delta$LL). **Boldface** indicates statistical significance. Dashes (—) indicate failure to improve over a baseline. $p$-values are corrected for false discovery rate (Benjamini and Yekutieli, 2001) across all comparisons per response variable.

to the deep neural IRF, while retaining them in the outputs. *Third*, analyses vary whether models assume a heteroscedastic exGaussian predictive distribution (present study) or a homoscedastic normal predictive distribution (Shain, 2019, note that a homoscedastic exGaussian distribution is not explored here because such a model would force an arbitrary choice as to whether the predictors modulate the location parameter or the skewness parameter). This setup gives rise to seven possible deviations from the design used in the present study (in which frequency and predictability effects dissociate) towards the design used in Shain (2019, in which they did not). For minimal comparison, unless otherwise specified, all models in this analysis use the implementation from the present study (e.g., the same ensembling approach, stopping criteria, neural network IRF, and software versions), rather than the implementation details from Shain (2019). Thus, no model is an exact reproduction attempt of Shain (2019), since code and data for reproduction are already publicly available.

Results for all seven of these configurations are reported in **Table S7**, along with the results from the main models used in the present study, re-presented for ease of comparison. As shown, modeling assumptions affect results. The more flexible models used in the present study are also more sensitive, and enforcing the stricter and less plausible assumptions made by Shain (2019) leads to a decrease in sensitivity to the critical effects. In the models that are most similar to Shain (2019, bottom four rows of **Table S7**), frequency and predictability do not dissociate, replicating the key null result from Shain (2019). No single modeling dimension appears to be responsible for the difference in results between the two studies: assuming log-scale responses, a homoscedastic normal distribution, or linear and additive effects all tend to decrease the sensitivity of the test. This outcome, together with the fact that these stricter assumptions also greatly harm generalization performance (e.g., the homoscedastic additive model is over 60,000 test set log likehood points worse on the Dundee dataset and over 150,000 test set log likelihood points words on the Natural Stories SPR dataset, relative to the main model), favors the model design (and, by extension, the results) of the present study. Nonetheless, it should be stressed that the capacity to relax these assumptions did not exist at the time of the Shain (2019) study. The implausibility of these assumptions for some research questions played a key role in motivating the development of CDRNNs (Shain and Schuler, 2022).

# J Reanalysis excluding the largest responses

Reading time distributions have long right tails (e.g., Frank et al., 2013), and standard models that assume symmetric (e.g., normally-distributed) error can highly sensitive to large positive values for the dependent variable. As a consequence, such analyses risk finding effects that are driven by a small number of outlier

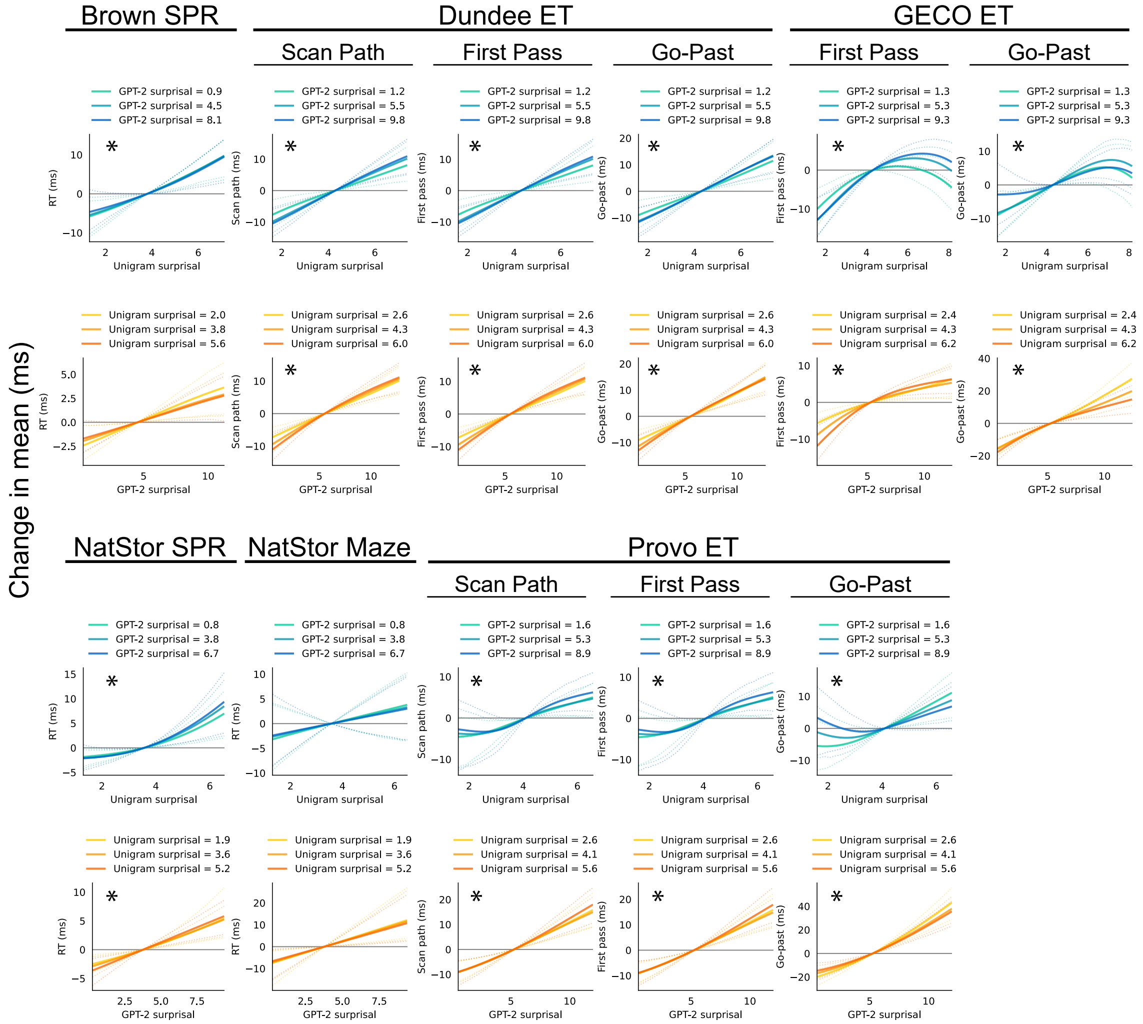


**Figure S13:** Estimated effects of frequency (*unigram surprisal*) and predictability (*GPT-2 surprisal*) on mean reading time (in ms) across datasets using a 95% cutoff (parallels **Figure 1**). Plots show how the instantaneous response (i.e., at no delay) deviates from its mean as a function of one predictor ($x$-axis) at three different values (line colors) of the other predictor (mean, $\pm 1$ standard deviation), thus revealing how the frequency effect changes as a function of predictability (and vice versa). Dotted lines show 95% variational Bayesian credible intervals. Plots showing effects that make a significant unique contribution to generalization likelihood are marked with $*$. In all datasets, effects of frequency are similar across the range of predictability values and vice versa, supporting a lack of interaction.

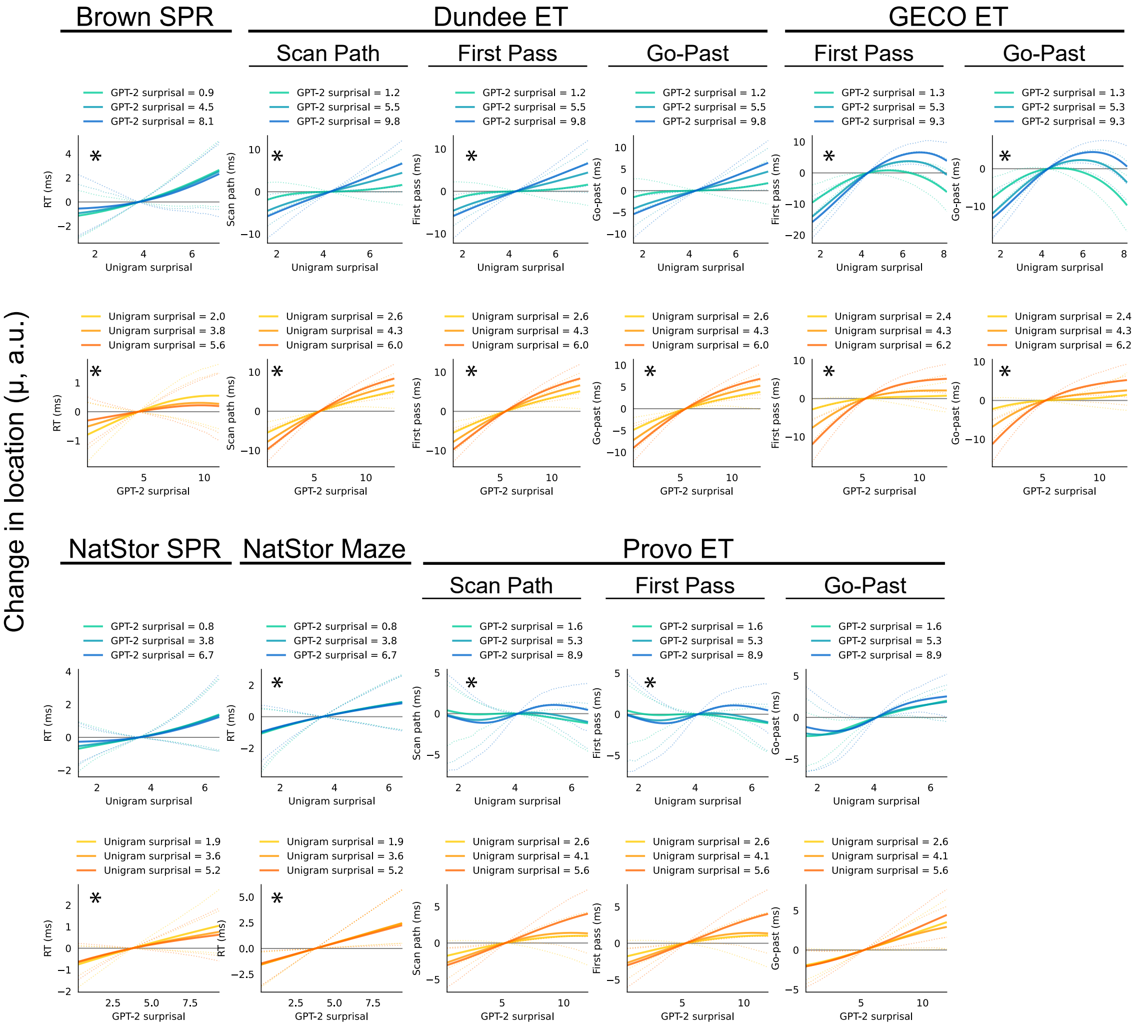


**Figure S14:** Estimated effects of frequency (*unigram surprisal*) and predictability (*GPT-2 surprisal*) on the exGaussian location parameter $\mu$ across datasets using a 95% cutoff (parallels **Figure 1**). Plots show how the instantaneous response (i.e., at no delay) deviates from its mean as a function of one predictor ($x$-axis) at three different values (line colors) of the other predictor (mean, $\pm 1$ standard deviation), thus revealing how the frequency effect changes as a function of predictability (and *vice versa*). Dotted lines show 95% variational Bayesian credible intervals. Plots showing effects that make a significant unique contribution to generalization likelihood are marked with $*$. Because CDRNNs implicitly rescale the dependent variable for efficient training, the units of $\mu$ are not straightforwardly interpretable (unlike effects on the mean) and are therefore notated as *a.u.* (arbitrary unit). In all datasets, effects of frequency are similar across the range of predictability values and *vice versa*, supporting a lack of interaction.

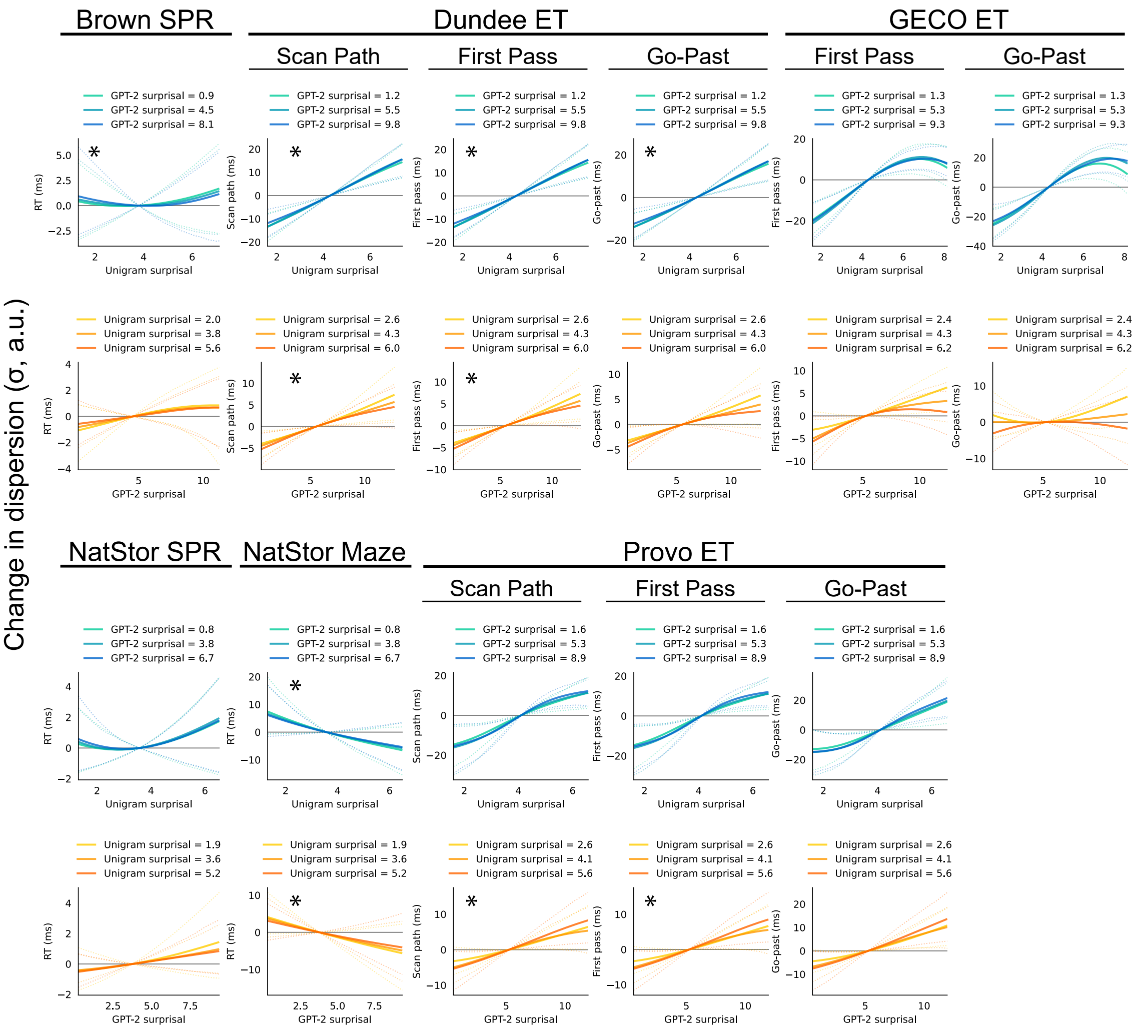


**Figure S15:** Estimated effects of frequency (*unigram surprisal*) and predictability (*GPT-2 surprisal*) on the exGaussian dispersion parameter $\sigma$ across datasets using a 95% cutoff (parallels **Figure 1**). Plots show how the instantaneous response (i.e., at no delay) deviates from its mean as a function of one predictor ($x$-axis) at three different values (line colors) of the other predictor (mean, $\pm$1 standard deviation), thus revealing how the frequency effect changes as a function of predictability (and *vice versa*). Dotted lines show 95% variational Bayesian credible intervals. Plots showing effects that make a significant unique contribution to generalization likelihood are marked with $*$. Because CDRNNs implicitly rescale the dependent variable for efficient training, the units of $\sigma$ are not straightforwardly interpretable (unlike effects on the mean) and are therefore notated as *a.u.* (arbitrary unit). In all datasets, effects of frequency are similar across the range of predictability values and *vice versa*, supporting a lack of interaction.

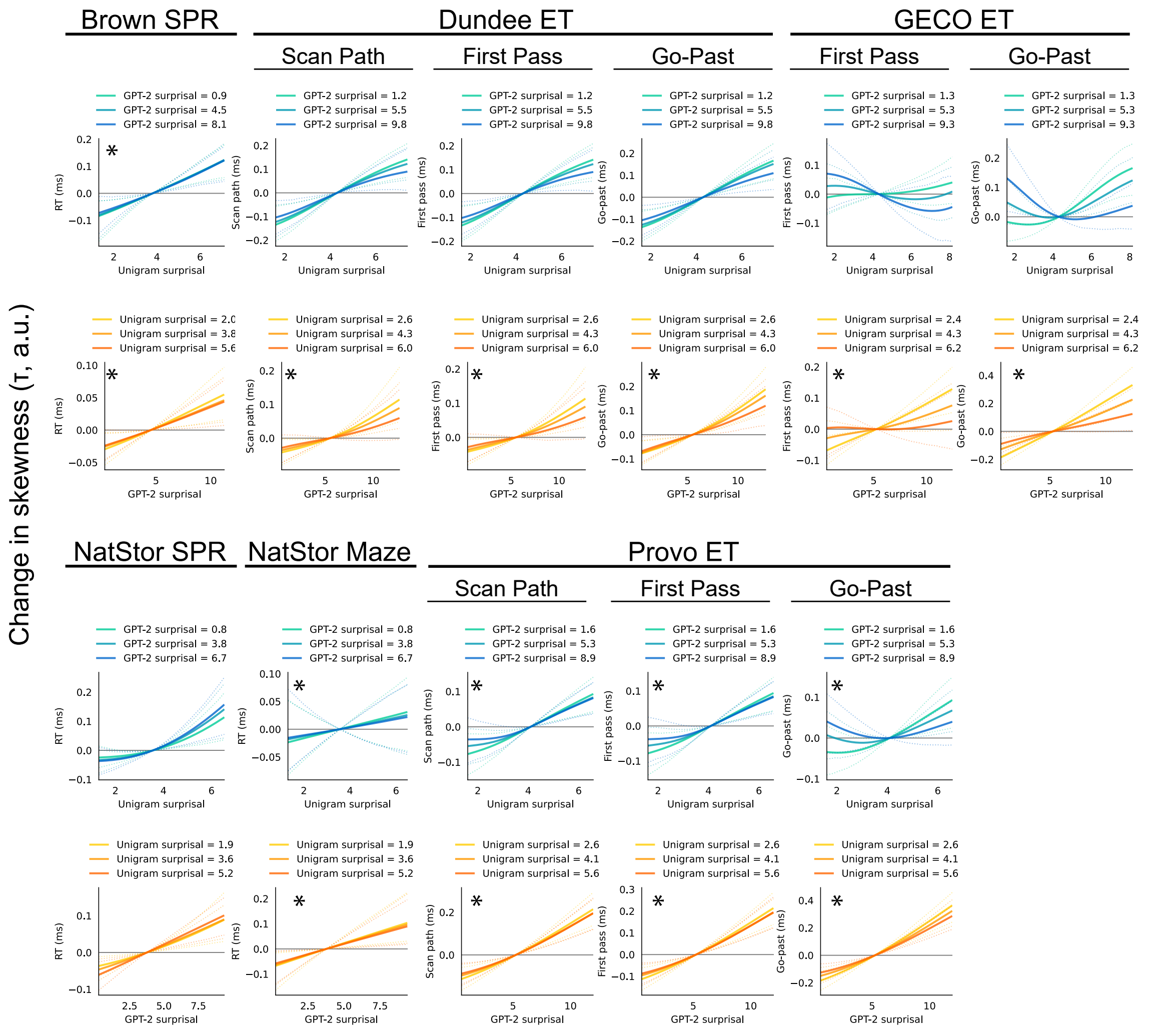


**Figure S16:** Estimated effects of frequency (*unigram surprisal*) and predictability (*GPT-2 surprisal*) on the exGaussian skewness parameter $\tau$ across datasets using a 95% cutoff (parallels **Figure 1**). Plots show how the instantaneous response (i.e., at no delay) deviates from its mean as a function of one predictor ($x$-axis) at three different values (line colors) of the other predictor (mean, $\pm$1 standard deviation), thus revealing how the frequency effect changes as a function of predictability (and *vice versa*). Dotted lines show 95% variational Bayesian credible intervals. Plots showing effects that make a significant unique contribution to generalization likelihood are marked with $*$. Because CDRNNs implicitly rescale the dependent variable for efficient training, the units of the $\tau$ are not straightforwardly interpretable (unlike effects on the mean) and are therefore notated as *a.u.* (arbitrary unit). In all datasets, effects of frequency are similar across the range of predictability values and *vice versa*, supporting a lack of interaction.

| Comparison | Scan Path ΔLL | Scan Path $p$ | First Pass ΔLL | First Pass $p$ | Go Past ΔLL | Go Past $p$ |
|---|---|---|---|---|---|---|
| Frequency | **431** | **0.0005** | **433** | **0.0005** | **435** | **0.0005** |
| Predictability | **337** | **0.0005** | **340** | **0.0005** | **375** | **0.0005** |
| Frequency over predictability | **194** | **0.0005** | **192** | **0.0005** | **192** | **0.0005** |
| Predictability over frequency | **101** | **0.0005** | **98** | **0.0005** | **132** | **0.0005** |
| Frequency-predictability interaction | -30 | — | -30 | — | -33 | — |
| Bigram predictability | -55 | — | -51 | — | -35 | — |
| Frequency over bigram predictability | **87** | **0.0005** | **90** | **0.0005** | **102** | **0.0005** |
| GPT-2 predictability over bigram predictability | -7 | — | -4 | — | **28** | **0.0100** |
| Trigram predictability | -59 | — | -60 | — | -46 | — |
| Frequency effect on $\mu$ | **29** | **0.0012** | **30** | **0.0014** | 14 | 0.2449 |
| Frequency effect on $\sigma$ | **90** | **0.0005** | **91** | **0.0005** | **56** | **0.0005** |
| Frequency effect on $\beta$ | 16 | 0.0889 | 15 | 0.1042 | -8 | — |
| Predictability effect on $\mu$ | **73** | **0.0005** | **74** | **0.0005** | **49** | **0.0005** |
| Predictability effect on $\sigma$ | **20** | **0.0199** | **20** | **0.0180** | -17 | — |
| Predictability effect on $\beta$ | **67** | **0.0005** | **67** | **0.0005** | **58** | **0.0005** |

**Table S9:** Testing results on Dundee (eye-tracking) using a 95% cutoff (parallels **Table S3**). Results of key statistical comparisons based on permutation tests of the difference in median test set likelihood between the alternative and null model in each comparison (ΔLL). **Boldface** indicates statistical significance. Dashes (—) indicate failure to improve over a baseline. $p$-values are corrected for false discovery rate (Benjamini and Yekutieli, 2001) across all comparisons per response variable.

| Comparison | First Pass ΔLL | First Pass $p$ | Go Past ΔLL | Go Past $p$ |
|---|---|---|---|---|
| Frequency | **162** | **0.0005** | **143** | **0.0005** |
| Predictability | **211** | **0.0005** | **283** | **0.0005** |
| Frequency over predictability | **85** | **0.0005** | **105** | **0.0005** |
| Predictability over frequency | **134** | **0.0005** | **245** | **0.0005** |
| Frequency-predictability interaction | **54** | **0.0005** | **37** | **0.0005** |
| Bigram predictability | 10 | 0.4147 | -15 | — |
| Frequency over bigram predictability | **60** | **0.0005** | **35** | **0.0005** |
| GPT-2 predictability over bigram predictability | **88** | **0.0005** | **141** | **0.0005** |
| Trigram predictability | -21 | — | **19** | **0.0095** |
| Frequency effect on $\mu$ | **41** | **0.0005** | **55** | **0.0005** |
| Frequency effect on $\sigma$ | 6 | 0.6818 | 9 | 0.1765 |
| Frequency effect on $\beta$ | 4 | 1.0000 | 9 | 0.2414 |
| Predictability effect on $\mu$ | **40** | **0.0005** | **34** | **0.0005** |
| Predictability effect on $\sigma$ | 1 | 1.0000 | -1 | — |
| Predictability effect on $\beta$ | **17** | **0.0005** | **94** | **0.0005** |

**Table S10:** Testing results on GECO (eye-tracking) using a 95% cutoff (parallels **Table S4**). Results of key statistical comparisons based on permutation tests of the difference in median test set likelihood between the alternative and null model in each comparison (ΔLL). **Boldface** indicates statistical significance. Dashes (—) indicate failure to improve over a baseline. $p$-values are corrected for false discovery rate (Benjamini and Yekutieli, 2001) across all comparisons per response variable.

| Comparison | Scan Path | | First Pass | | Go Past | |
|---|---|---|---|---|---|---|
| | ΔLL | $p$ | ΔLL | $p$ | ΔLL | $p$ |
| Frequency | **91** | **0.0006** | **90** | **0.0006** | **67** | **0.0006** |
| Predictability | **104** | **0.0006** | **104** | **0.0006** | **128** | **0.0006** |
| Frequency over predictability | **33** | **0.0006** | **32** | **0.0006** | **18** | **0.0006** |
| Predictability over frequency | **46** | **0.0006** | **46** | **0.0006** | **79** | **0.0006** |
| Frequency-predictability interaction | **36** | **0.0006** | **36** | **0.0006** | 11 | 0.1139 |
| Bigram predictability | 0 | 1.0000 | -1 | — | 7 | 0.4606 |
| Frequency over bigram predictability | **12** | **0.0328** | 9 | 0.1593 | **26** | **0.0006** |
| GPT-2 predictability over bigram predictability | 11 | 0.0846 | 9 | 0.2592 | **47** | **0.0006** |
| Trigram predictability | 7 | 0.5603 | 8 | 0.2592 | 7 | 0.5591 |
| Frequency effect on $\mu$ | **13** | **0.0017** | **13** | **0.0033** | -3 | — |
| Frequency effect on $\sigma$ | 2 | 1.0000 | -1 | — | 7 | 0.1662 |
| Frequency effect on $\beta$ | **22** | **0.0006** | **20** | **0.0006** | **15** | **0.0006** |
| Predictability effect on $\mu$ | 2 | 1.0000 | 0 | 1.0000 | 4 | 1.0000 |
| Predictability effect on $\sigma$ | **23** | **0.0006** | **17** | **0.0006** | 1 | 1.0000 |
| Predictability effect on $\beta$ | **23** | **0.0006** | **19** | **0.0006** | **59** | **0.0006** |

**Table S11:** Testing results on Provo (eye-tracking) using a 95% cutoff (parallels **Table S5**). Results of key statistical comparisons based on permutation tests of the difference in median test set likelihood between the alternative and null model in each comparison (ΔLL). **Boldface** indicates statistical significance. Dashes (—) indicate failure to improve over a baseline. $p$-values are corrected for false discovery rate (Benjamini and Yekutieli, 2001) across all comparisons per response variable.

| Comparison | Combined Datasets | |
|---|---|---|
| | ΔLL | $p$ |
| Frequency | **2064** | **0.0003** |
| Predictability | **2488** | **0.0003** |
| Frequency over predictability | **1087** | **0.0003** |
| Predictability over frequency | **1511** | **0.0003** |
| Frequency-predictability interaction | -561 | — |
| Bigram predictability | **413** | **0.0003** |
| Frequency over bigram predictability | **1237** | **0.0003** |
| GPT-2 predictability over bigram predictability | **305** | **0.0003** |
| Trigram predictability | -225 | — |
| Frequency effect on $\mu$ | -368 | — |
| Frequency effect on $\sigma$ | **704** | **0.0003** |
| Frequency effect on $\beta$ | **538** | **0.0003** |
| Predictability effect on $\mu$ | **2137** | **0.0003** |
| Predictability effect on $\sigma$ | **1401** | **0.0003** |
| Predictability effect on $\beta$ | **1078** | **0.0003** |

**Table S12:** Testing results across all datasets using a 95% cutoff (parallels **Table S6**). Results of key statistical comparisons based on permutation tests of the difference in median test set likelihood between the alternative and null model in each comparison (ΔLL). **Boldface** indicates statistical significance. Dashes (—) indicate failure to improve over a baseline. $p$-values are corrected for false discovery rate (Benjamini and Yekutieli, 2001) across all comparisons.

datapoints. In the present study, this concern is substantially mitigated by a combination of outlier removal for many datasets, following prior precedent (**Section 2.1**), and exGaussian regression, which captures rightward skew in the distribution over reading times and thus provides a close fit to the skewed empirical distributions of responses in these datasets (**SI E**). Nonetheless, all models are fitted and evaluated on data that retain a substantial chunk of the right tail (this is by design, since effects on the tail, captured by the skewness parameter $\tau$, are of scientific interest here). To assess the sensitivity of findings to datapoints in the tail, this section presents results obtained by first removing the top 5% of reading times from each dataset prior to applying any other preprocessing and then re-running preprocessing and analyses that are otherwise identical to those described in the main article.

Estimated frequency and predictability effects on the mean response under a 95% cutoff are plotted in **Figure S13** (which parallels **Figure 1** of the main article), and estimated effects on the $\mu$, $\sigma$, and $\tau$ parameters of the exGaussian distribution are plotted in **Figures S14–S16** (which respectively parallel **Figures S10–S12**). Estimates from models under a 95% frequency cutoff are similar to estimates from the models in the main analysis: (i) frequency and predictability both modulate mean reading time as predicted, (ii) frequency and predictability each show similar effects across values of the other variable, suggesting little interaction, and (iii) frequency and predictability effects are generally strongest on the $\tau$ parameter. The main qualitative difference from the estimates in the main analyses is that effects on the remaining two parameters ($\mu$ and $\sigma$) appear to be larger, with more consistent positive modulation of each parameter. These visualizations thus suggest that frequency and predictability not only increase skewness, but also shift the reading time distribution upward ($\mu$) and increase its spread ($\tau$).

Results of statistical tests under a 95% cutoff are reported in **Tables S8–S12** (parallel to **Tables S2–S6**). Tests of the key main effects (frequency over predictability and frequency over predictability) produce identical results across all datasets to those in the main analyses. There are some dataset-level differences in other tests that do not lead to differences in aggregate tests: for example, the GECO dataset did not show a frequency-predictability interaction in the main analyses, but does show one when a 95% cutoff is applied. As suggested by the visualizations in **Figures S14–S16**, the key overall difference from applying a 95% cutoff is that the tests become more sensitive to frequency and predictability effects on individual parameters of the exGaussian distribution: whereas no distributional effects are significant overall in the main analyses (**Table S6**), all distributional effects except the frequency effect on the location parameter $\mu$ are significant in the analyses that use a 95% cutoff (**Table S12**). It is thus possible that predictability and frequency differ in their effect on $\mu$ in ways that are less evident when more of the tail is included in the analyses, but it is also possible that removing more of the tail impairs the model's ability to estimate its shape. In any case, the distributional finding in these follow-up analyses (i.e., that both frequency and predictability modulate $\mu$, $\sigma$, and that predictability modulates $\tau$, with no evidence that frequency modulates $\tau$) is substantially different from that advocated in prior work (that frequency and predictability modulate $\mu$ and that only frequency modulates $\tau$, with neither modulating $\sigma$ Staub, 2015). This issue warrants further investigation.

Differences in distributional results aside, the critical findings from the main analyses are reproduced under a 95% cutoff: in aggregate tests, there are significant unique effects of both frequency and predictability, and no significant interaction between them (**Table S12**). Thus, results using a 95% cutoff support an additive dissociation between frequency and predictability effects in reading, which reinforces the core theoretical claims of this study.

# K Reanalysis using linear mixed-effects models

A key motivation for the present study is the recent development of CDRNNs, which (as reviewed in the **Introduction** and discussed at length in Shain and Schuler, 2018, 2021; Shain, 2021; Shain and Schuler, 2022) offer important advantages for modeling continuous reading relative to standard approaches in psycholinguistics like linear mixed-effects models (LMEMs; Bates et al., 2015). Since CDRNNs are comparatively novel, this section presents parallel analyses of the frequency-predictability relationship in the same reading datasets using more familiar LMEMs.

| | Dataset | Response | Test LL | Freq $\beta$ | Freq $p$ | Pred $\beta$ | Pred $p$ | Freq over Pred $\beta$ | Freq over Pred $p$ | Pred over Freq $\beta$ | Pred over Freq $p$ | Interaction $\beta$ | Interaction $p$ |
|---|---|---|---|---|---|---|---|---|---|---|---|---|---|
| In-sample (LRT) | Brown SPR | RT | -189598 | 6.28 | < **0.0001** | 7.75 | < **0.0001** | 4.12 | < **0.0001** | 7.16 | < **0.0001** | 1.56 | < **0.0001** |
| | Dundee ET | Scan path | -389901 | 13.72 | < **0.0001** | 15.17 | < **0.0001** | 5.91 | < **0.0001** | 12.68 | < **0.0001** | 6.22 | < **0.0001** |
| | | First pass | -292968 | 14.22 | < **0.0001** | 15.70 | < **0.0001** | 6.22 | < **0.0001** | 13.11 | < **0.0001** | 6.20 | < **0.0001** |
| | | Go-past | -330375 | 18.04 | < **0.0001** | 20.34 | < **0.0001** | 7.53 | < **0.0001** | 17.21 | < **0.0001** | 8.31 | < **0.0001** |
| | GECO ET | First pass | -450096 | 5.18 | < **0.0001** | 11.23 | < **0.0001** | -0.58 | < **0.0001** | 11.43 | < **0.0001** | 0.91 | < **0.0001** |
| | | Go-past | -536680 | 11.53 | < **0.0001** | 22.81 | < **0.0001** | 0.06 | 0.1204 | 22.81 | < **0.0001** | 2.15 | < **0.0001** |
| | Natural Stories SPR | RT | -1210605 | 8.15 | < **0.0001** | 10.02 | < **0.0001** | 4.47 | < **0.0001** | 8.92 | < **0.0001** | 4.43 | < **0.0001** |
| | Natural Stories Maze | RT | -102159 | 60.51 | < **0.0001** | 132.37 | < **0.0001** | 8.67 | < **0.0001** | 130.38 | < **0.0001** | -6.70 | < **0.0001** |
| | Provo ET | Scan path | -238827 | 9.90 | < **0.0001** | 12.34 | < **0.0001** | 3.45 | **0.0001** | 11.13 | < **0.0001** | 5.79 | < **0.0001** |
| | | First pass | -165823 | 10.81 | < **0.0001** | 13.25 | < **0.0001** | 3.86 | < **0.0001** | 11.93 | < **0.0001** | 5.35 | < **0.0001** |
| | | Go-past | -189003 | 13.09 | < **0.0001** | 23.68 | < **0.0001** | -0.89 | 0.1591 | 24.02 | < **0.0001** | 10.43 | < **0.0001** |
| Out-of-sample (PT) | Brown SPR | RT | -190669 | 5.56 | **0.0028** | 7.59 | **0.0077** | 3.46 | **0.0067** | 7.09 | **0.0251** | 1.50 | **0.0431** |
| | Dundee ET | Scan path | -392714 | 13.75 | **0.0001** | 15.11 | **0.0001** | 5.94 | **0.0016** | 12.61 | **0.0001** | 6.10 | **0.0001** |
| | | First pass | -295661 | 14.46 | **0.0001** | 15.91 | **0.0001** | 6.34 | **0.0033** | 13.28 | **0.0001** | 6.35 | **0.0014** |
| | | Go-past | -333003 | 18.08 | **0.0004** | 20.24 | **0.0001** | 7.54 | 0.1551 | 17.16 | **0.0001** | 9.19 | 0.8260 |
| | GECO ET | First pass | -454179 | 4.93 | **0.0001** | 11.16 | **0.0001** | -0.85 | **0.0016** | 11.44 | **0.0001** | 0.94 | 0.0538 |
| | | Go-past | -539465 | 11.92 | 0.0675 | 22.99 | **0.0001** | 0.37 | 0.3364 | 22.89 | **0.0001** | 2.17 | **0.0483** |
| | Natural Stories SPR | RT | -1213235 | 7.94 | **0.0002** | 9.91 | **0.0001** | 4.28 | 0.0594 | 8.86 | **0.0001** | 4.39 | 0.6037 |
| | Natural Stories Maze | RT | -103702 | 58.73 | **0.0407** | 130.49 | **0.0001** | 8.06 | 0.7710 | 128.71 | **0.0001** | -6.18 | 0.4592 |
| | Provo ET | Scan path | -239395 | 10.04 | 0.8645 | 12.49 | 0.6848 | 3.48 | 0.8354 | 11.27 | 0.4656 | 5.32 | 0.0842 |
| | | First pass | -166365 | 10.65 | 0.2224 | 13.44 | 0.4842 | 3.50 | **0.0430** | 12.24 | 0.4580 | 5.19 | 0.1159 |
| | | Go-past | -189784 | 12.96 | 0.8664 | 24.03 | 0.6564 | -1.45 | 0.6081 | 24.55 | 0.5820 | 10.19 | 0.1079 |

**Table S13:** Results of statistical tests—both in-sample (likelihood ratio tests, *LRT*) and out-of-sample (permutation tests, *PT*)—comparing linear mixed-effects models (LMEMs) representing different hypotheses about frequency and predictability effects across reading datasets. Effects of frequency and predictability are tested individually against a null baseline model containing neither (tests *Freq* and *Pred*) and together against null models containing only one or the other (*Freq over Pred* and *Pred over Freq*). A full model containing a frequency-predictability interaction is also tested against an otherwise identical model that lacks the interaction term (*Interaction*). The *Test LL* column reports the likelihood assigned to the test set by the full model (i.e., the model that contains frequency, predictability, and frequency-predictability interaction terms).

Models are fitted using the `lme4` package in `R` (Bates et al., 2015) and used to test hypotheses under two different approaches to model comparison (i.e., comparing the fit of a model that contains the key effect of interest to the fit of one that lacks it). The first approach, standard in psycholinguistics and other fields, is likelihood ratio testing (LRT; Wilks, 1938). LRT is an in-sample method that compares the fit of models to the data to which they were fitted, using asymptotic properties of the likelihood ratio statistic. The second approach is the same as that used in the main CDRNN analyses (**SI A.3**): permutation testing (PT) of the conditional likelihood obtained by fitting models to part of the data (the training and validation sets used in the CDRNN analyses) and evaluating them on out-of-sample data (the test set). The advantage of PT over LRT is that it directly accounts for the degree to which the estimated contribution of a given effect generalizes to a different sample of data.

Models are designed to be as similar to the main CDRNN models as is feasible. Fixed effects include an intercept, a slope for each control predictor used in the CDRNN analyses, and a slope for each critical predictor used in a given model, namely: frequency (unigram surprisal), predictability (GPT-2 surprisal), and/or their interaction. Random effects include intercepts by participant and item (defined in these naturalistic datasets as token position within the document) and slopes by subject for any fixed slope contained in a given model. To facilitate convergence, all predictors are z-scored prior to fitting. Random effects were constrained to be uncorrelated following widespread convergence failures in models with correlated random effects (which suggests the models are overspecified and need to be simplified). Lagged regressors ("spillover" effects) led to convergence errors and were therefore not included. All models successfully converged with no warnings. Although these models resemble the CDRNN models in the types of fixed and random effects they include, they differ in several potentially important other ways. This point is revisited below.

Test results are given in **Table S13**. As shown, under in-sample tests, nearly every comparison is significant. In-sample tests thus support the key claim of the present study that frequency effects are not explained by predictability (and *vice versa*), although they do not support the claim that the two effects are additive (since the interaction is also significant). Under stricter out-of-sample tests, the main effect of predictability is significant over frequency in 8/11 comparisons, the main effect of frequency is significant over predictability in 5/11 comparisons, and the frequency-predictability interaction is significant in 4/11 comparisons. Out-of-sample tests thus also provide some support for dissociable effects, albeit considerably weaker. LMEM-based results thus align in key ways with the central claim of this study.

However, as argued in the **Introduction** and at length in prior work (Shain and Schuler, 2018, 2021;

Shain, 2021; Shain and Schuler, 2022), many likely sources of variation are left uncontrolled by these LMEMs: they do not capture delayed effects, nonlinearities, or interactions (at least, not without additional regressors like "spillover" terms that complicate these models to the point of unidentifiability, as discussed above); they do not capture change in the response function over time; and they do not capture effects on the full distribution over reading times. Existing evidence renders all of these simplifications implausible for naturalistic reading, and failure to account for them can qualitatively affect the outcomes of statistical tests (Baayen et al., 2017; Shain and Schuler, 2022). Moreover, the difference between in-sample and out-of-sample results in **Table S13** highlights a potential danger for standard hypothesis testing procedures in psycholinguistics when applied to naturalistic reading: effects found by these LMEMs (and identified as significant by in-sample tests) do not reliably generalize in many cases. The advantages of CDRNNs over LMEMs for naturalistic reading are more than conceptual: CDRNNs also substantially improve fit, and thus, the generalizability of effects (the LMEM-based test-set likelihoods in **Table S13** fall far below those of CDRNN models in **Table S1**, even for in-sample tests using LMEMs that were trained on the test data; see also Shain, 2021; Shain and Schuler, 2022). It is of course possible that frequency and predictability are distinct but interact in naturalistic reading—as suggested by the in-sample LMEM results (which most closely resemble standard practice in psycholinguistics)—and that CDRNN models simply fail to detect them. However, deep neural networks like CDRNNs excel at capturing interactions (LeCun et al., 2015) to the point that machine learning research has focused on methods to *prevent* the discovery of spurious interactions (e.g., He et al., 2016). Even the (generally nonlinear) impulse response functions (IRFs) of CDRNNs are implemented underlyingly as interactions between predictors and a *delay* variable indexing the temporal offset between predictors and responses; CDRNNs readily recover ground-truth nonlinear IRFs via these interactions (Shain, 2021; Shain and Schuler, 2022). In summary, to infer the existence of generalized frequency-predictability interactions from the results in **Table S13**, one must selectively rely on models that not only enforce implausible independence assumptions but also fit the data less well: when more expressive CDRNN models are used, evidence of an interaction is greatly attenuated (**Figure 1**). Therefore, both conceptual and empirical considerations favor the main results of the article over those presented in **Table S13** and thus additive frequency and predictability effects, although the absence of interaction is admittedly inferred from null results and could thus be challenged by more sensitive methods in future research. Such a finding would nuance but not fundamentally alter the key theoretical contribution of this study: frequency effects in naturalistic reading are not explained by standard suprisal theory and thus require theoretical revision (for example, by positing additional costs related to memory retrieval or by incorporating memory constraints into the inference model, see **Discussion**).

# L Model formulae

For ease of reference, this section provides the CDRNN model formulae used throughout this study. Variable names are changed from those used in the codebase for readability. For full software implementation details, see `https://github.com/coryshain/cdr`. Because the sets of control predictors differ across datasets, control terms (e.g., "WordLength + SaccadeLength + EndOfSentence + ...") are abbreviated as "CONTROLS". Control variables used for each dataset are described in **SI A.1**. Note that in the CDRNN implementation, the skewness parameter is referred to as $\beta$, rather than $\tau$ (as in the main article).

- **Main 1:** y $\sim$ C(CONTROLS + Unigram Surprisal + GPT-2 Surprisal, NN()) + (C(CONTROLS + Unigram Surprisal + GPT-2 Surprisal, NN(ran=T)) | Participant) + (1 | DocumentID:SentenceID:WordPositionInSentence)
- **Main 1, no frequency:** y $\sim$ C(CONTROLS + GPT-2 Surprisal, NN()) + (C(CONTROLS + GPT-2 Surprisal, NN(ran=T)) | Participant) + (1 | DocumentID:SentenceID:WordPositionInSentence)
- **Main 1 1, no predictability:** y $\sim$ C(CONTROLS + GPT-2 Surprisal, NN()) + (C(CONTROLS + GPT-2 Surprisal, NN(ran=T)) | Participant) + (1 | DocumentID:SentenceID:WordPositionInSentence)

- **Main, no frequency or predictability:** y ~ C(CONTROLS, NN()) + (C(CONTROLS, NN(ran=T)) | Participant) + (1 | DocumentID:SentenceID:WordPositionInSentence)
- **Main 2:** y ~ C(CONTROLS + Unigram Surprisal + GPT-2 Surprisal, NN()) + C(CONTROLS + Unigram Surprisal + GPT-2 Surprisal, NN()) + (C(CONTROLS + Unigram Surprisal + GPT-2 Surprisal, NN(ran=T)) + C(CONTROLS + Unigram Surprisal + GPT-2 Surprisal, NN(ran=T)) | Participant) + + (1 | DocumentID:SentenceID:WordPositionInSentence)
- **Main 2, no interaction:** y ~ C(CONTROLS + Unigram Surprisal, NN()) + C(CONTROLS + GPT-2 Surprisal, NN()) + (C(CONTROLS + Unigram Surprisal, NN()) + C(CONTROLS + GPT-2 Surprisal, NN()) | Participant) + + (1 | DocumentID:SentenceID:WordPositionInSentence)
- **Main 3:** y ~ C(CONTROLS + Unigram Surprisal + GPT-2 Surprisal, NN(pred_params=[mu])) + C(CONTROLS + Unigram Surprisal + GPT-2 Surprisal, NN(pred_params=[sigma])) + C(CONTROLS + Unigram Surprisal + GPT-2 Surprisal, NN(pred_params=[beta])) + (C(CONTROLS + Unigram Surprisal + GPT-2 Surprisal, NN(pred_params=[mu])) + C(CONTROLS + Unigram Surprisal + GPT-2 Surprisal, NN(pred_params=[sigma])) + C(CONTROLS + Unigram Surprisal + GPT-2 Surprisal, NN(pred_params=[beta])) | Participant) + + (1 | DocumentID:SentenceID:WordPositionInSentence)
- **Main 3, no frequency effect on location:** y ~ C(CONTROLS + GPT-2 Surprisal, NN(pred_params=[mu])) + C(CONTROLS + Unigram Surprisal + GPT-2 Surprisal, NN(pred_params=[sigma])) + C(CONTROLS + Unigram Surprisal + GPT-2 Surprisal, NN(pred_params=[beta])) + (C(CONTROLS + GPT-2 Surprisal, NN(pred_params=[mu])) + C(CONTROLS + Unigram Surprisal + GPT-2 Surprisal, NN(pred_params=[sigma])) + C(CONTROLS + Unigram Surprisal + GPT-2 Surprisal, NN(pred_params=[beta])) | Participant) + + (1 | DocumentID:SentenceID:WordPositionInSentence)
- **Main 3, no frequency effect on dispersion:** y ~ C(CONTROLS + Unigram Surprisal + GPT-2 Surprisal, NN(pred_params=[mu])) + C(CONTROLS + GPT-2 Surprisal, NN(pred_params=[sigma])) + C(CONTROLS + Unigram Surprisal + GPT-2 Surprisal, NN(pred_params=[beta])) + (C(CONTROLS + Unigram Surprisal + GPT-2 Surprisal, NN(pred_params=[mu])) + C(CONTROLS + GPT-2 Surprisal, NN(pred_params=[sigma])) + C(CONTROLS + Unigram Surprisal + GPT-2 Surprisal, NN(pred_params=[beta])) | Participant) + + (1 | DocumentID:SentenceID:WordPositionInSentence)
- **Main 3, no frequency effect on skewness:** y ~ C(CONTROLS + Unigram Surprisal + GPT-2 Surprisal, NN(pred_params=[mu])) + C(CONTROLS + Unigram Surprisal + GPT-2 Surprisal, NN(pred_params=[sigma])) + C(CONTROLS + GPT-2 Surprisal, NN(pred_params=[beta])) + (C(CONTROLS + Unigram Surprisal + GPT-2 Surprisal, NN(pred_params=[mu])) + C(CONTROLS + Unigram Surprisal + GPT-2 Surprisal, NN(pred_params=[sigma])) + C(CONTROLS + GPT-2 Surprisal, NN(pred_params=[beta])) | Participant) + + (1 | DocumentID:SentenceID:WordPositionInSentence)
- **Main 3, no predictability effect on location:** y ~ C(CONTROLS + Unigram Surprisal, NN(pred_params=[mu])) + C(CONTROLS + Unigram Surprisal + GPT-2 Surprisal, NN(pred_params=[sigma])) + C(CONTROLS + Unigram Surprisal + GPT-2 Surprisal, NN(pred_params=[beta])) + (C(CONTROLS + Unigram Surprisal, NN(pred_params=[mu])) + C(CONTROLS + Unigram Surprisal + GPT-2 Surprisal, NN(pred_params=[sigma])) + C(CONTROLS + Unigram Surprisal + GPT-2 Surprisal, NN(pred_params=[beta])) | Participant) + + (1 | DocumentID:SentenceID:WordPositionInSentence)
- **Main 3, no predictability effect on dispersion:** y ~ C(CONTROLS + Unigram Surprisal + GPT-2 Surprisal, NN(pred_params=[mu])) + C(CONTROLS + Unigram Surprisal,

NN(pred_params=[sigma])) + C(CONTROLS + Unigram Surprisal + GPT-2 Surprisal, NN(pred_params=[beta])) + (C(CONTROLS + Unigram Surprisal + GPT-2 Surprisal, NN(pred_params=[mu])) + C(CONTROLS + Unigram Surprisal, NN(pred_params=[sigma])) + C(CONTROLS + Unigram Surprisal + GPT-2 Surprisal, NN(pred_params=[beta])) | Participant) + + (1 | DocumentID:SentenceID:WordPositionInSentence)

- **Main 3, no predictability effect on skewness:** y ∼ C(CONTROLS + Unigram Surprisal + GPT-2 Surprisal, NN(pred_params=[mu])) + C(CONTROLS + Unigram Surprisal + GPT-2 Surprisal, NN(pred_params=[sigma])) + C(CONTROLS + Unigram Surprisal, NN(pred_params=[beta])) + (C(CONTROLS + Unigram Surprisal + GPT-2 Surprisal, NN(pred_params=[mu])) + C(CONTROLS + Unigram Surprisal + GPT-2 Surprisal, NN(pred_params=[sigma])) + C(CONTROLS + Unigram Surprisal, NN(pred_params=[beta])) | Participant) + + (1 | DocumentID:SentenceID:WordPositionInSentence)

- **Bigram:** y ∼ C(CONTROLS + Unigram Surprisal + GPT-2 Surprisal + Bigram Surprisal, NN()) + (C(CONTROLS + Unigram Surprisal + GPT-2 Surprisal + Bigram Surprisal, NN(ran=T)) | Participant) + (1 | DocumentID:SentenceID:WordPositionInSentence)

- **Bigram, no GPT-2:** y ∼ C(CONTROLS + Unigram Surprisal + Bigram Surprisal, NN()) + (C(CONTROLS + Unigram Surprisal + Bigram Surprisal, NN(ran=T)) | Participant) + (1 | DocumentID:SentenceID:WordPositionInSentence)

- **Bigram, no frequency:** y ∼ C(CONTROLS + GPT-2 Surprisal + Bigram Surprisal, NN()) + (C(CONTROLS + GPT-2 Surprisal + Bigram Surprisal, NN(ran=T)) | Participant) + (1 | DocumentID:SentenceID:WordPositionInSentence)

- **Trigram:** y ∼ C(CONTROLS + Unigram Surprisal + GPT-2 Surprisal + Bigram Surprisal + Trigram Surprisal, NN()) + (C(CONTROLS + Unigram Surprisal + GPT-2 Surprisal + Bigram Surprisal + Trigram Surprisal, NN(ran=T)) | Participant) + (1 | DocumentID:SentenceID:WordPositionInSentence)

# References


Altarriba, J., Kroll, J. F., Sholl, A., and Rayner, K. (1996). The influence of lexical and conceptual constraints on reading mixed-language sentences: Evidence from eye fixations and naming times. *Memory & Cognition*, 24(4):477–492.

Ashby, J., Rayner, K., and Clifton, C. (2005). Eye Movements of Highly Skilled and Average Readers: Differential Effects of Frequency and Predictability. *The Quarterly Journal of Experimental Psychology Section A*, 58(6):1065–1086. Publisher: SAGE Publications.

Baayen, H., Vasishth, S., Kliegl, R., and Bates, D. (2017). The cave of shadows: Addressing the human factor with generalized additive mixed models. *Journal of Memory and Language*, 94(Supplement C):206–234.

Bates, D., Mächler, M., Bolker, B., and Walker, S. (2015). Fitting linear mixed-effects models using lme4. *Journal of Statistical Software*, 67(1):1–48.

Benjamini, Y. and Yekutieli, D. (2001). The control of the false discovery rate in multiple testing under dependency. *The Annals of Statistics*, 29(4):1165–1188.

Breen, M. (2014). Empirical investigations of the role of implicit prosody in sentence processing. *Language and Linguistics Compass*, 8(2):37–50. Publisher: Wiley Online Library.

Bélanger, N. N. and Rayner, K. (2013). Frequency and predictability effects in eye fixations for skilled and less-skilled deaf readers. *Visual Cognition*, 21(4):477–497. Publisher: Routledge _eprint: https://doi.org/10.1080/13506285.2013.804016.

Frank, S. L., Fernandez Monsalve, I., Thompson, R. L., and Vigliocco, G. (2013). Reading time data for evaluating broad-coverage models of English sentence processing. *Behavior Research Methods*, 45(4):1182–1190.

Gal, Y. and Ghahramani, Z. (2016). Dropout as a Bayesian Approximation: Representing Model Uncertainty in Deep Learning. In *Proceedings of the 33rd International Conference on Machine Learning*.

Gollan, T. H., Slattery, T. J., Goldenberg, D., Van Assche, E., Duyck, W., and Rayner, K. (2011). Frequency drives lexical access in reading but not in speaking: The frequency-lag hypothesis. *Journal of Experimental Psychology: General*, 140:186–209. Place: US Publisher: American Psychological Association.

Goodkind, A. and Bicknell, K. (2021). Local word statistics affect reading times independently of surprisal, arXiv. arXiv:2103.04469 [cs], http://arxiv.org/abs/2103.04469 (Accessed: 2022-10-30).

Hand, C., Miellet, S., O'Donnell, P., and Sereno, S. (2010). The Frequency-Predictability Interaction in Reading: It Depends Where You're Coming From. *Journal of experimental psychology. Human perception and performance*, 36:1294–313.

He, K., Zhang, X., Ren, S., and Sun, J. (2016). Deep Residual Learning for Image Recognition. In *2016 IEEE Conference on Computer Vision and Pattern Recognition (CVPR)*, pages 770–778, Las Vegas, NV, USA. IEEE.

Kretzschmar, F., Schlesewsky, M., and Staub, A. (2015). Dissociating word frequency and predictability effects in reading: Evidence from coregistration of eye movements and EEG. *Journal of Experimental Psychology: Learning, Memory, and Cognition*, 41(6):1648–1662.

Lavigne, F., Vitu, F., and d'Ydewalle, G. (2000). The influence of semantic context on initial eye landing sites in words. *Acta Psychologica*, 104(2):191–214.

LeCun, Y., Bengio, Y., and Hinton, G. (2015). Deep learning. *Nature*, 521(7553):436–444. Number: 7553 Publisher: Nature Publishing Group.

Miellet, S., Sparrow, L., and Sereno, S. C. (2007). Word frequency and predictability effects in reading French: An evaluation of the E-Z Reader model. *Psychonomic Bulletin & Review*, 14(4):762–769.

Nelson, M. J., El Karoui, I., Giber, K., Yang, X., Cohen, L., Koopman, H., Cash, S. S., Naccache, L., Hale, J. T., Pallier, C., and Dehaene, S. (2017). Neurophysiological dynamics of phrase-structure building during sentence processing. *Proceedings of the National Academy of Sciences*, 114(18):E3669–E3678. Publisher: Proceedings of the National Academy of Sciences.

Norris, D. (2006). The Bayesian reader: Explaining word recognition as an optimal Bayesian decision process. *Psychological Review*, 113(2):327–357. Num Pages: 327-357 Place: Washington, US Publisher: American Psychological Association (US).

Prasad, G. and Linzen, T. (2021). Rapid syntactic adaptation in self-paced reading: Detectable, but only with many participants. *Journal of Experimental Psychology. Learning, Memory, and Cognition*, 47(7):1156–1172.

Rayner, K., Ashby, J., Pollatsek, A., and Reichle, E. D. (2004). The Effects of Frequency and Predictability on Eye Fixations in Reading: Implications for the E-Z Reader Model. *Journal of Experimental Psychology: Human Perception and Performance*, 30:720–732. Place: US Publisher: American Psychological Association.

Rayner, K., Binder, K. S., Ashby, J., and Pollatsek, A. (2001). Eye movement control in reading: word predictability has little influence on initial landing positions in words. *Vision Research*, 41(7):943–954.

Shain, C. (2019). A Large-Scale Study of the Effects of Word Frequency and Predictability in Naturalistic Reading. In *Proceedings of the 2019 Conference of the North American Chapter of the Association for Computational Linguistics: Human Language Technologies, Volume 1 (Long and Short Papers)*, pages 4086–4094.

Shain, C. (2021). CDRNN: Discovering Complex Dynamics in Human Language Processing. In *Proceedings of the 59th Annual Meeting of the Association for Computational Linguistics and the 11th International Joint Conference on Natural Language Processing (Volume 1: Long Papers)*, pages 3718–3734, Online. Association for Computational Linguistics.

Shain, C., Meister, C., Pimentel, T., Cotterell, R., and Levy, R. P. (to appear). Large-Scale Evidence for Logarithmic Effects of Word Predictability on Reading Time. *Proceedings of the National Academy of Sciences*.

Shain, C. and Schuler, W. (2018). Deconvolutional time series regression: A technique for modeling temporally diffuse effects. In *Proceedings of the 2018 Conference on Empirical Methods in Natural Language Processing*.

Shain, C. and Schuler, W. (2021). Continuous-time deconvolutional regression for psycholinguistic modeling. *Cognition*, 215:104735.

Shain, C. and Schuler, W. (2022). A Deep Learning Approach to Analyzing Continuous-Time Systems, arXiv. http://arxiv.org/abs/2209.12128 (Accessed: 2022-10-30).

Srivastava, N., Hinton, G., Krizhevsky, A., Sutskever, I., and Salakhutdinov, R. (2014). Dropout: A Simple Way to Prevent Neural Networks from Overfitting. *Journal of Machine Learning Research*, 15(56):1929–1958.

Staub, A. (2015). The effect of lexical predictability on eye movements in reading: Critical review and theoretical interpretation. *Language and Linguistics Compass*, 9(8):311–327. Publisher: Wiley Online Library.

Wilks, S. S. (1938). The Large-Sample Distribution of the Likelihood Ratio for Testing Composite Hypotheses. *The Annals of Mathematical Statistics*, 9(1):60–62. Publisher: Institute of Mathematical Statistics.

Winkler, A. M., Ridgway, G. R., Webster, M. A., Smith, S. M., and Nichols, T. E. (2014). Permutation inference for the general linear model. *NeuroImage*, 92(100):381–397.
